# Supplementary material for: A New Advanced Backcross Tomato Population Enables High Resolution Leaf QTL Mapping and Gene Identification
Source: G3 (Bethesda). 2016 Aug 10;6(10):3169–84. doi: 10.1534/g3.116.030536 (PMC5068939; doi:10.1534/g3.116.030536)
Supplement: Supplemental Material [file supp_6_10_3169__index.html]

A New Advanced Backcross Tomato Population Enables High Resolution Leaf QTL Mapping and Gene Identification — Supplemental Material 

# A New Advanced Backcross Tomato Population Enables High Resolution Leaf QTL Mapping and Gene Identification

## Supplemental Material for Fulop, *et al*, 2016

**Files in this Data Supplement:**

- Supplemental Figures - This file contains Figures S1-S73. (.pdf, 15,459 KB)
- Table S1 - Broad-sense heritability (H2) and repeatability (R2) estimates. (.pdf, 50 KB)
- Table S2 - Permutation tests for candidate gene enrichment of BIL single locus QTL using a literature-curated leaf developmental gene list together with *cis*-eQTL and radical amino acid change (PROVEAN) data. (.pdf, 48 KB)
- Table S3 - IL *trans*-eQTL enrichment among BIL epistatic QTL. (.pdf, 59 KB)
- Table S4 - Genes in the overlapping interval of the chromosome 5 ﬂowering QTL in ILs and BILs. (.pdf, 59 KB)
- File S1 - Introgressions by BIL. (.xlsx, 135 KB)
- File S2 - Introgressions by chromosome. (.xlsx, 142 KB)
- File S3 - Bin locations and genotypes. (.zip, 43 KB)
- File S4 - Annotated genes per BIN. (.csv, 3373 KB)
- File S5 - Marginal regression QTL mapping results. (.zip, 5306 KB)
- File S6 - SparseNet QTL mapping results. (.zip, 144 KB)
- File S7 - Single locus QTL candidate genes. (.zip, 22 KB)
- File S8 - Epistatic QTL candidate genes. (.zip, 656 KB)
- File S9 - IL-BIL fine-mapping tables. (.zip, 18 KB)
