# Supplementary material for: A New Advanced Backcross Tomato Population Enables High Resolution Leaf QTL Mapping and Gene Identification
Source: G3 (Bethesda). 2016 Aug 10;6(10):3169–84. doi: 10.1534/g3.116.030536 (PMC5068939; doi:10.1534/g3.116.030536)
Supplement: Supplemental Material [file supp_g3.116.030536_SupplementalFigures.pdf]

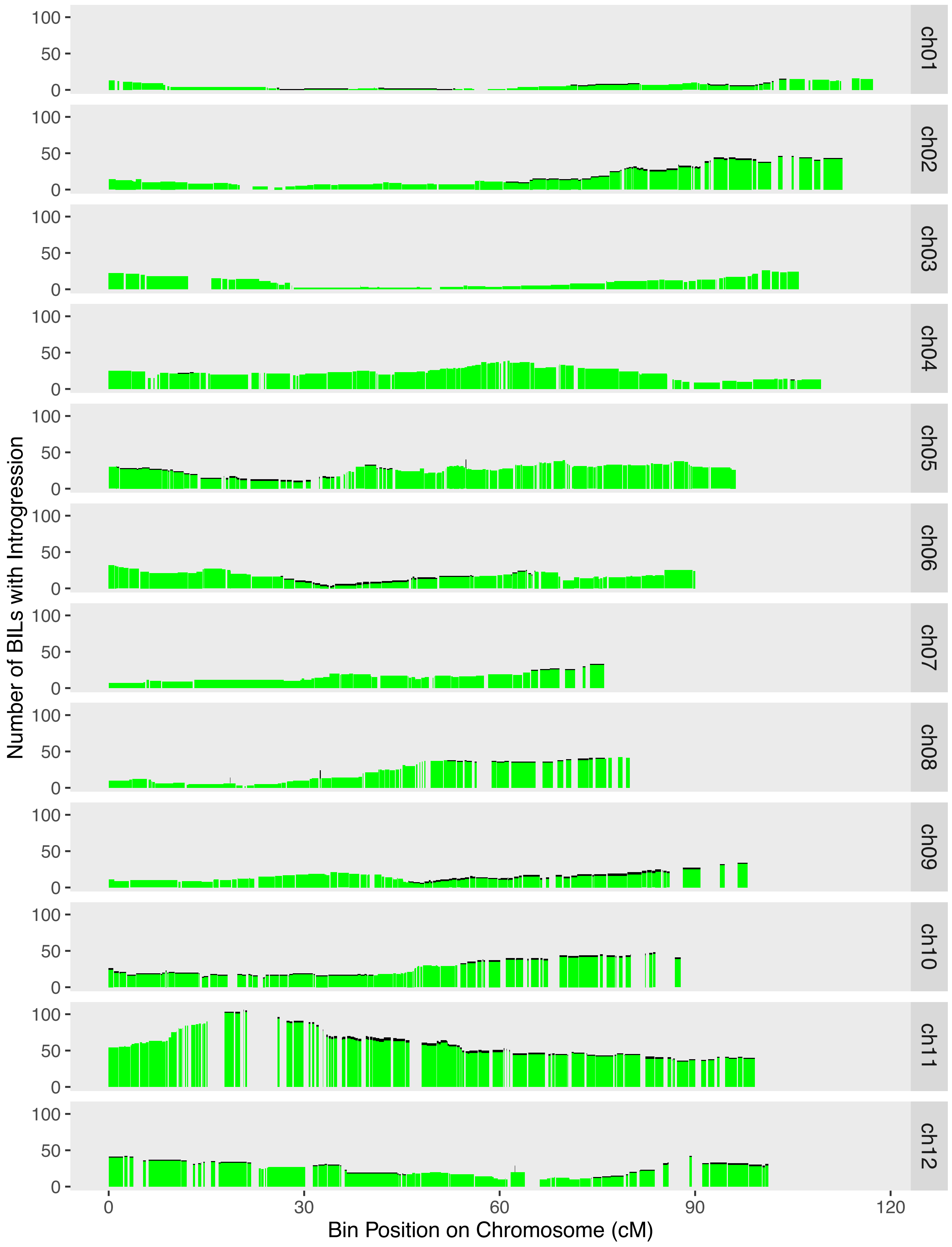

Figure S1. Distribution of introgressions across bins in genetic distance. Gaps in the bin distribution are the product of merging nearby recombination boundaries when defining bins.

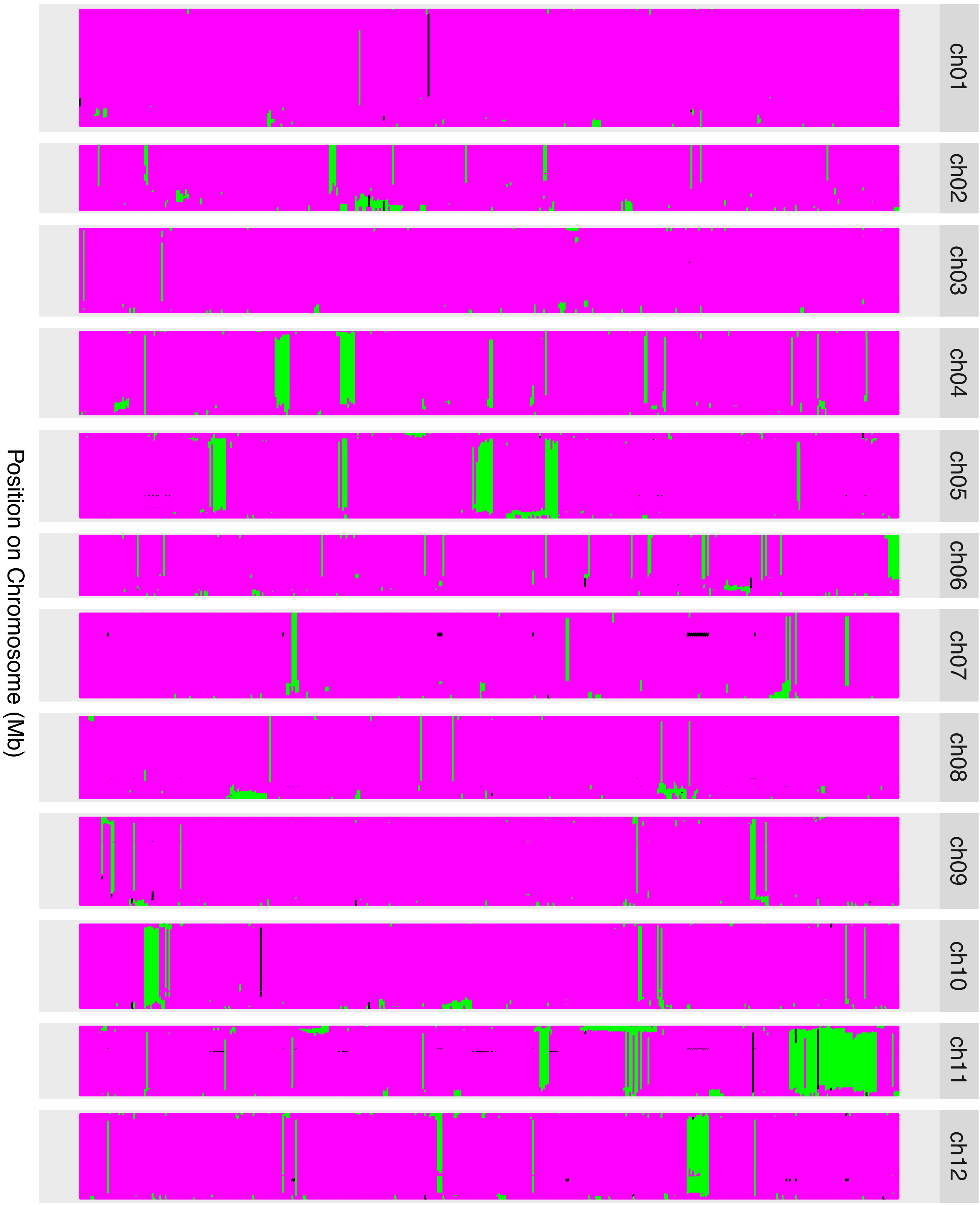

Figure S2. Composite genotype map of the entire BIL population in physical distance. The three genotypes are denoted as: M82 = magenta, heterozygous = black, *S. penellii* = green.

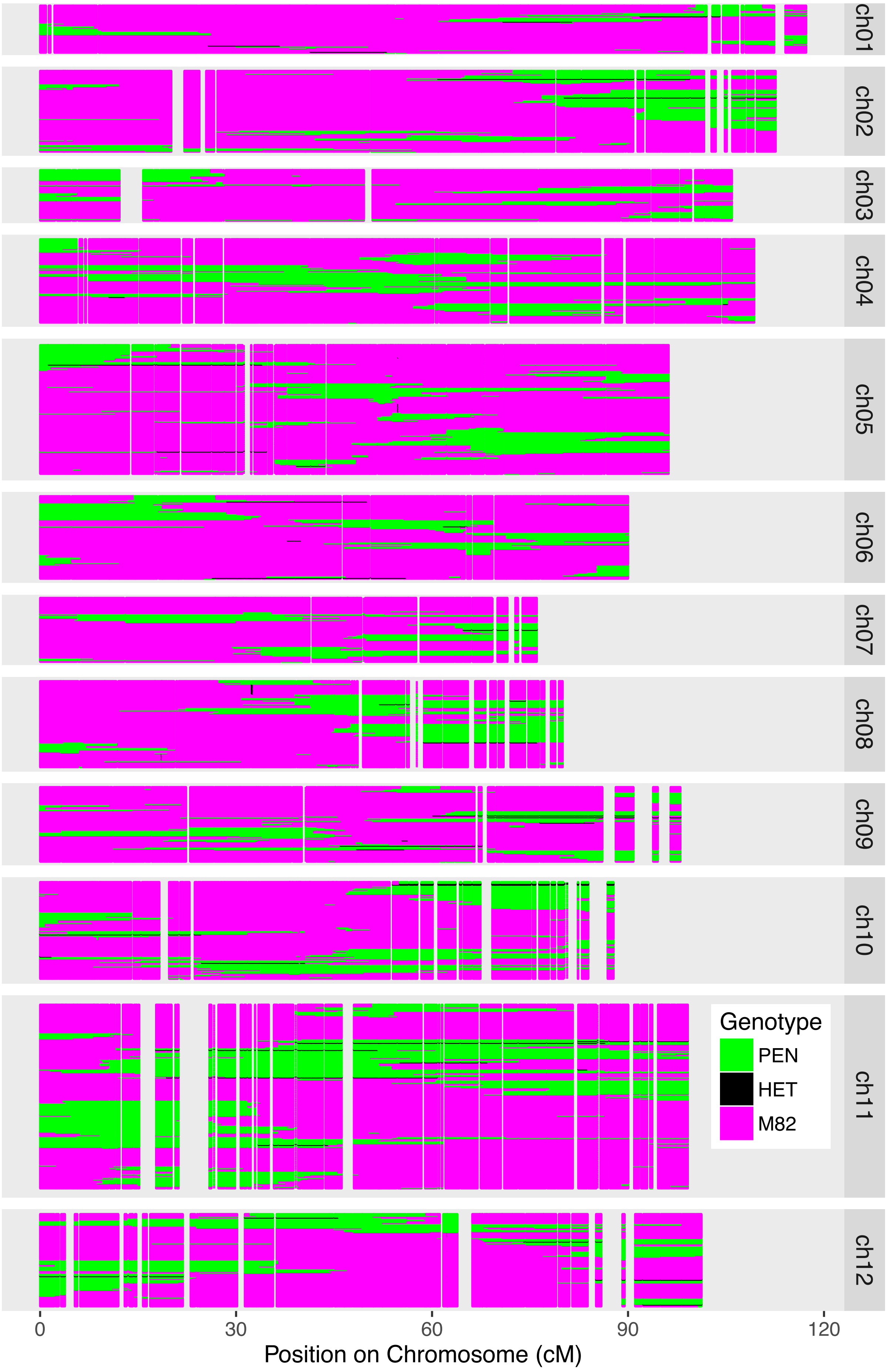

Figure S3. Composite genotype map of the entire BIL population in genetic distance with BILs clustered by each chromosome after removing samples without an introgression. Gaps are the product of merging nearby recombination boundaries when defining bins.

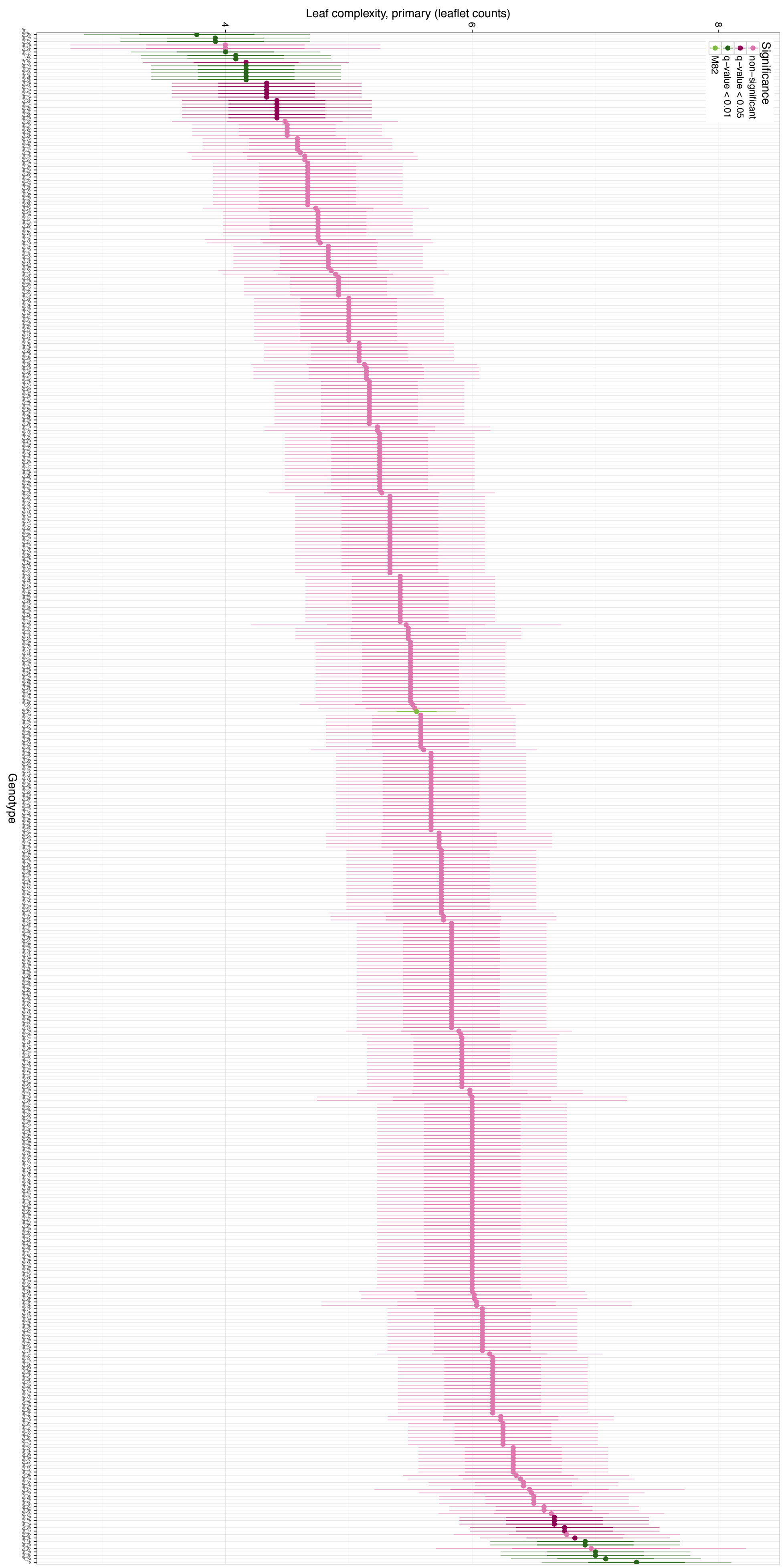

Figure S4. Primary leaf complexity BIL means. The thick line is the standard deviation, and the thin line is the approximate 95% CI. Significance coloring is in comparison to M82.

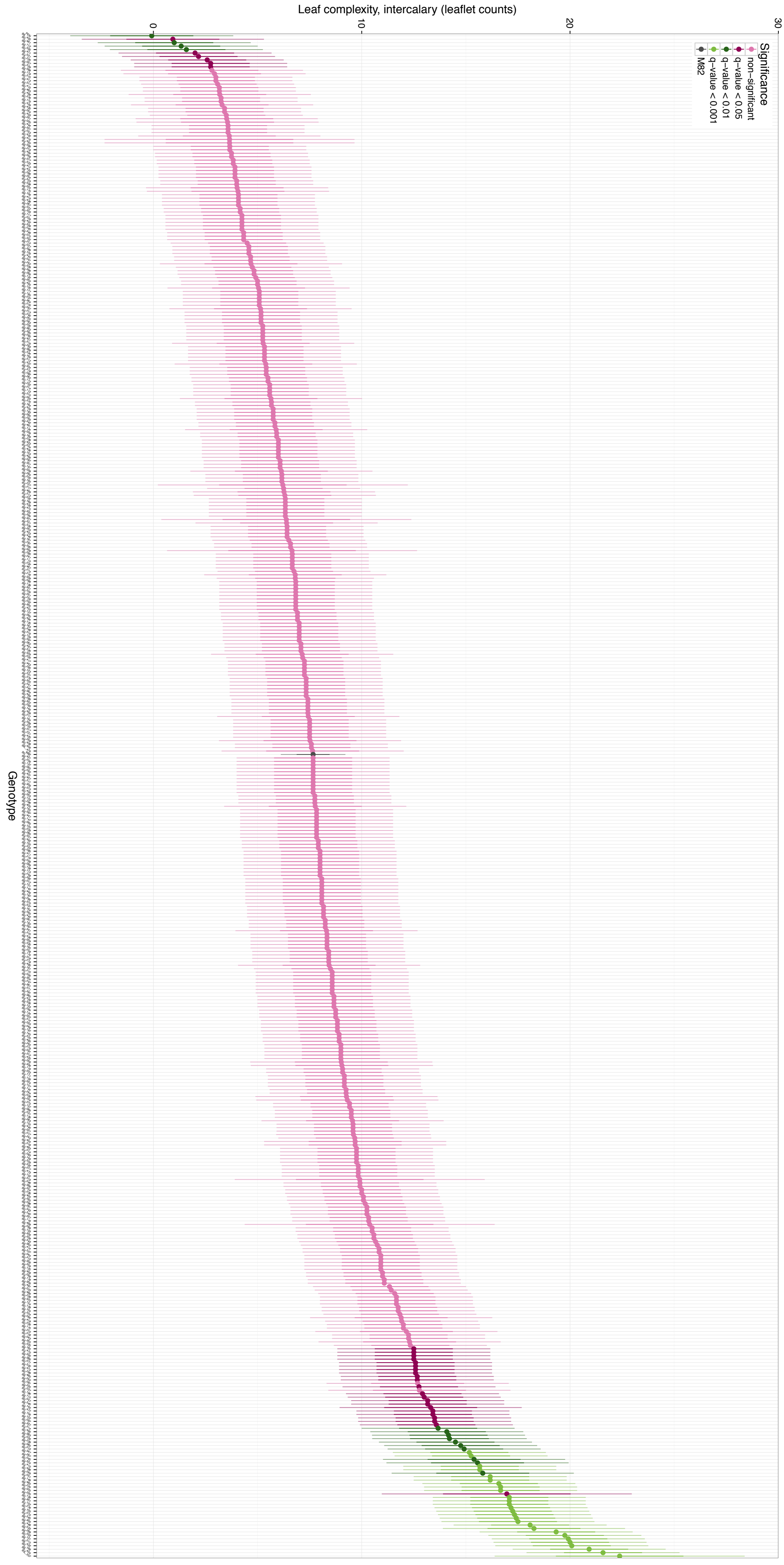

Figure S5. Intercalary leaf complexity BIL means. The thick line is the standard deviation, and the thin line is the approximate 95% CI. Significance coloring is in comparison to M82.

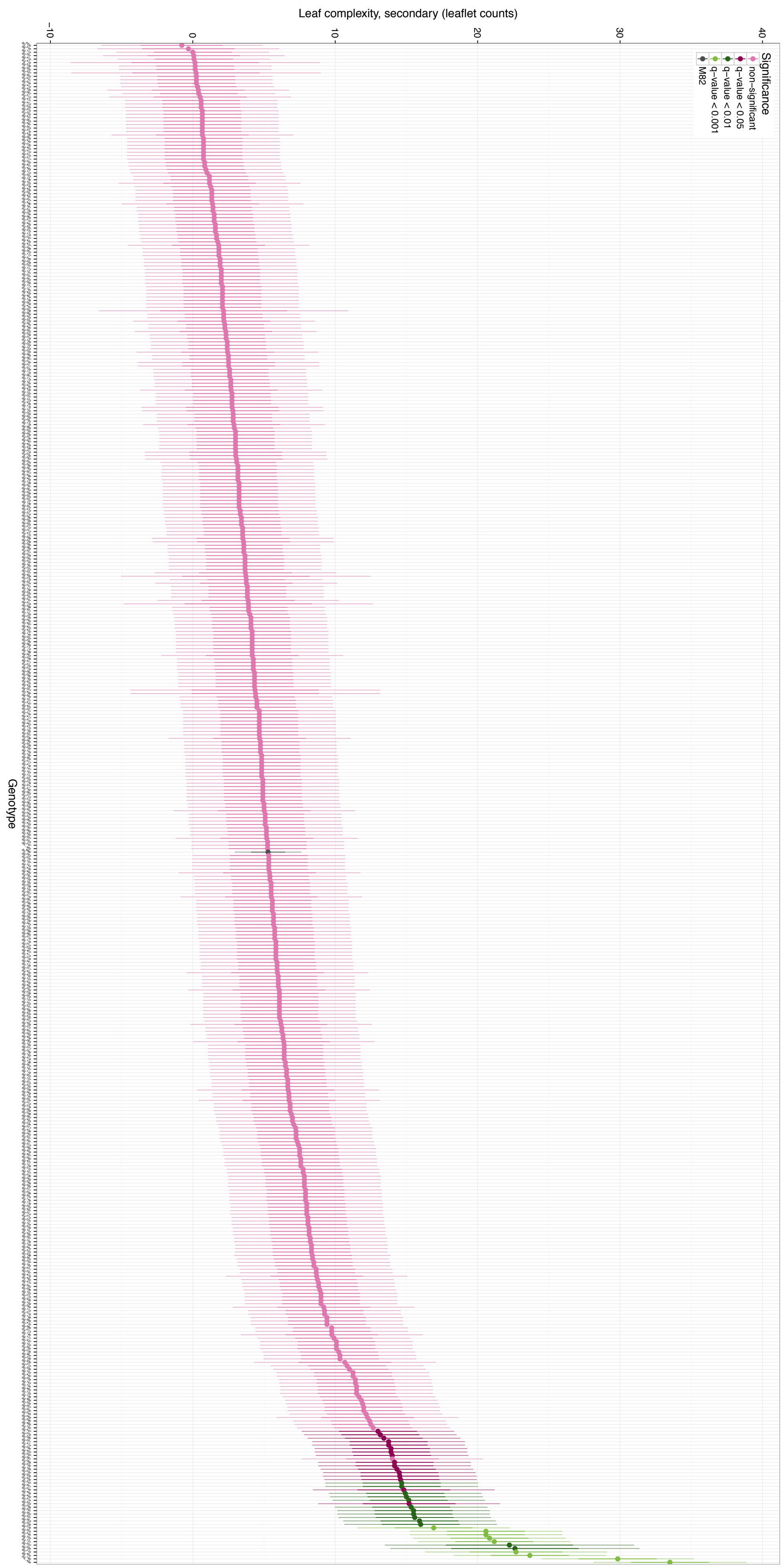

Figure S6. Secondary leaf complexity BIL means. The thick line is the standard deviation, and the thin line is the approximate 95% CI. Significance coloring is in comparison to M82.

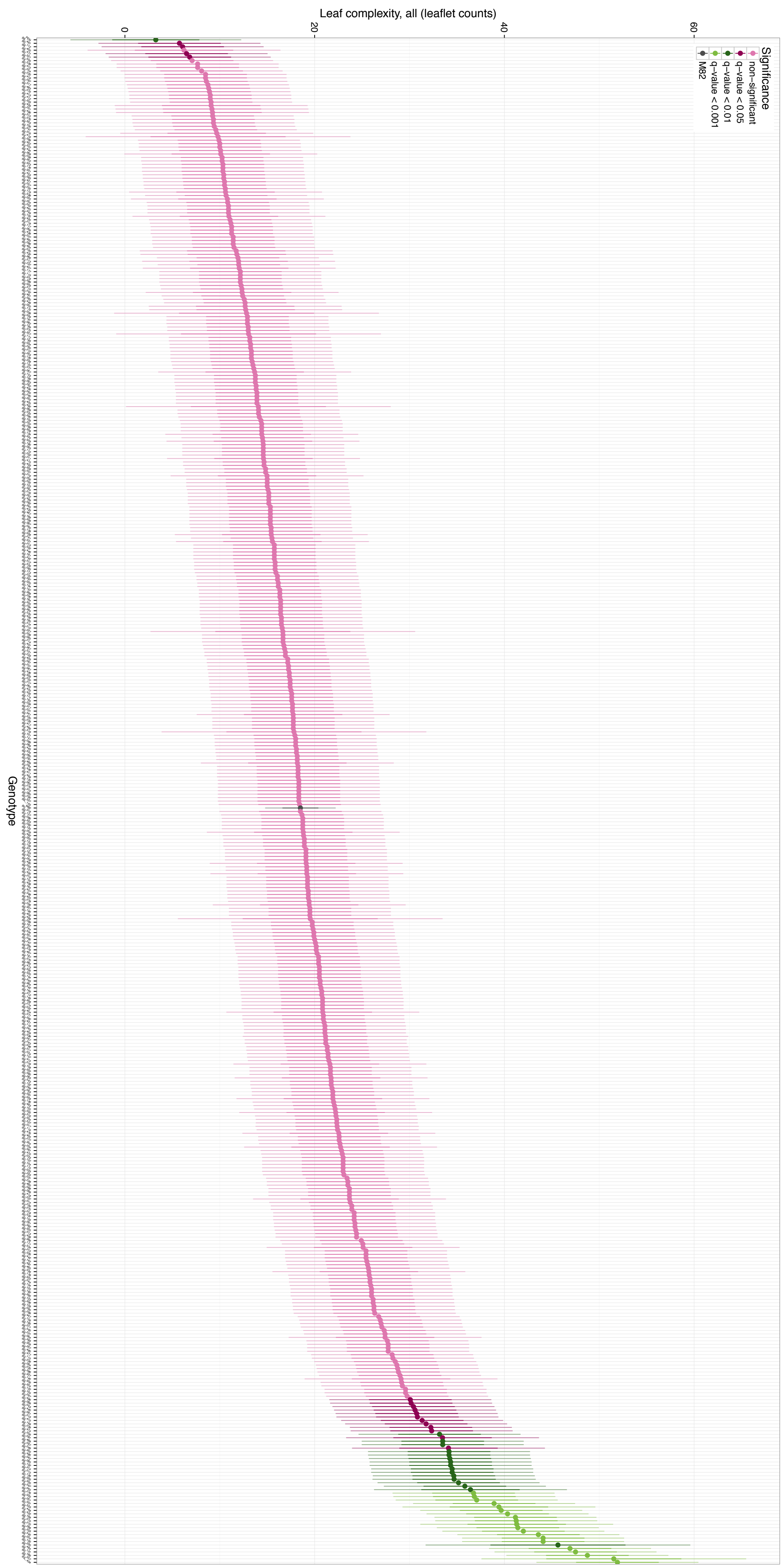

Figure S7. Total leaf complexity BIL means. The thick line is the standard deviation, and the thin line is the approximate 95% CI. Significance coloring is in comparison to M82.

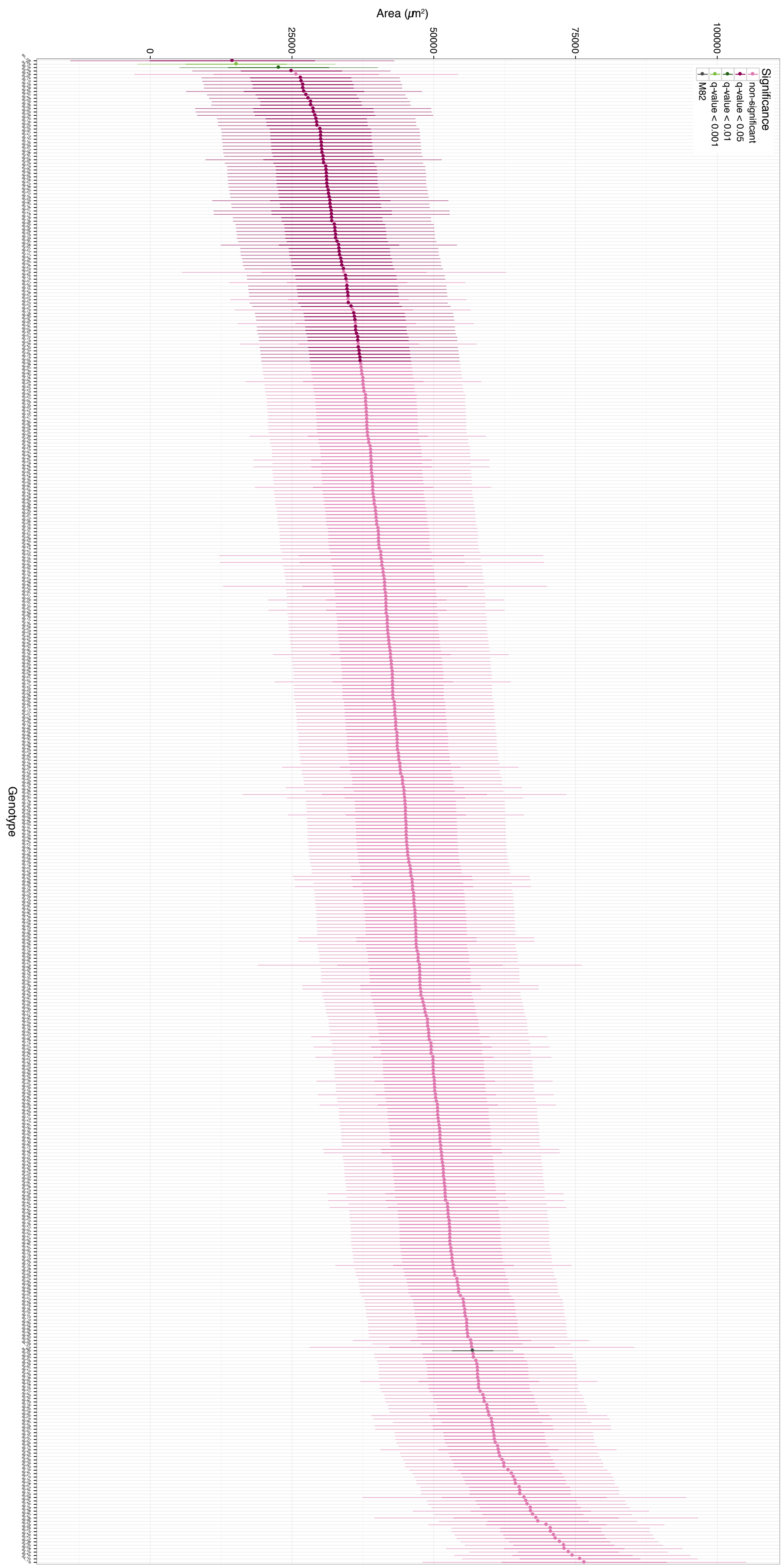

Figure S8. Leaflet area BIL means. The thick line is the standard deviation, and the thin line is the approximate 95% CI. Significance coloring is in comparison to M82.

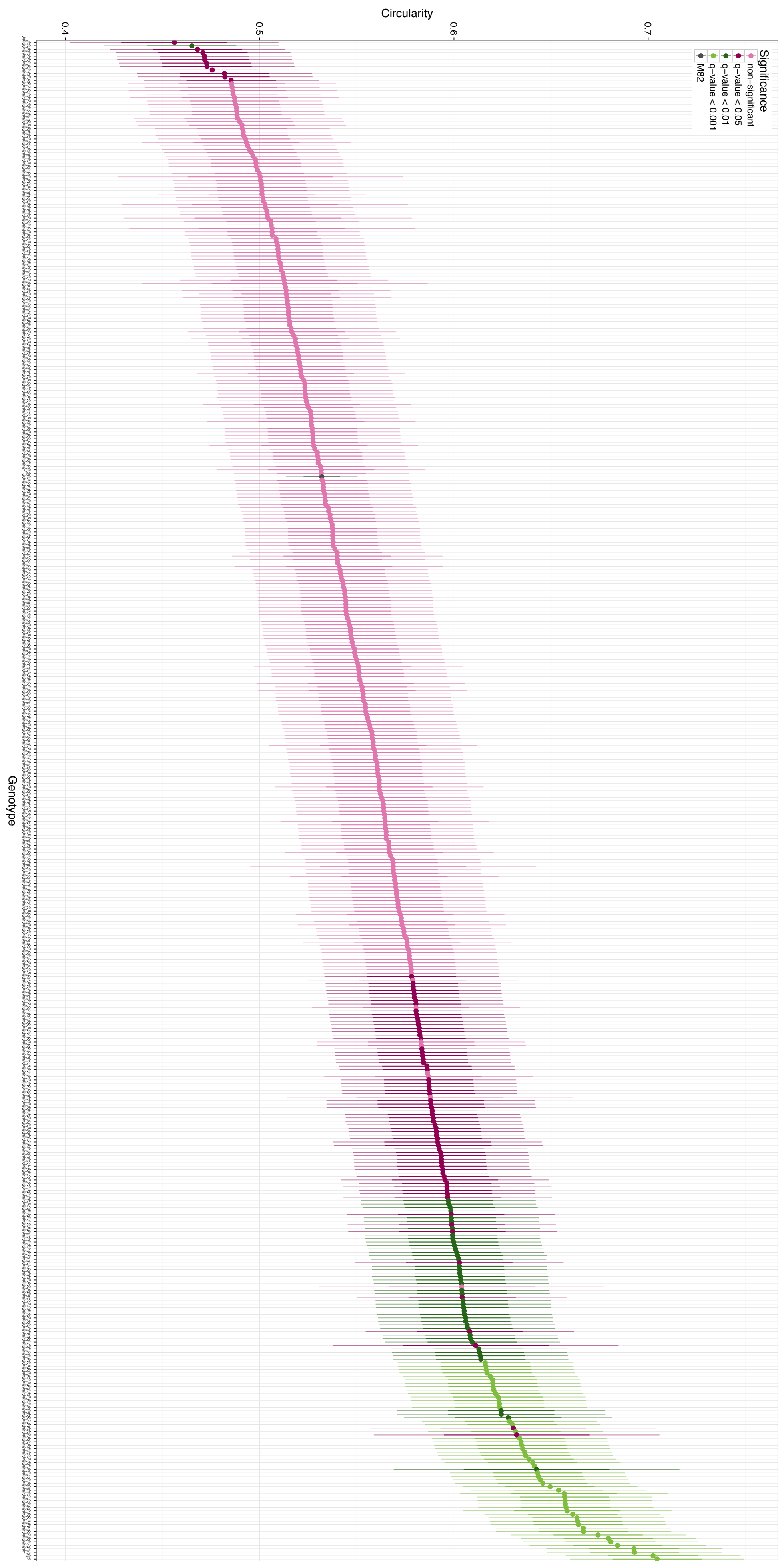

Figure S9. Leaflet circularity BIL means. The thick line is the standard deviation, and the thin line is the approximate 95% CI. Significance coloring is in comparison to M82.

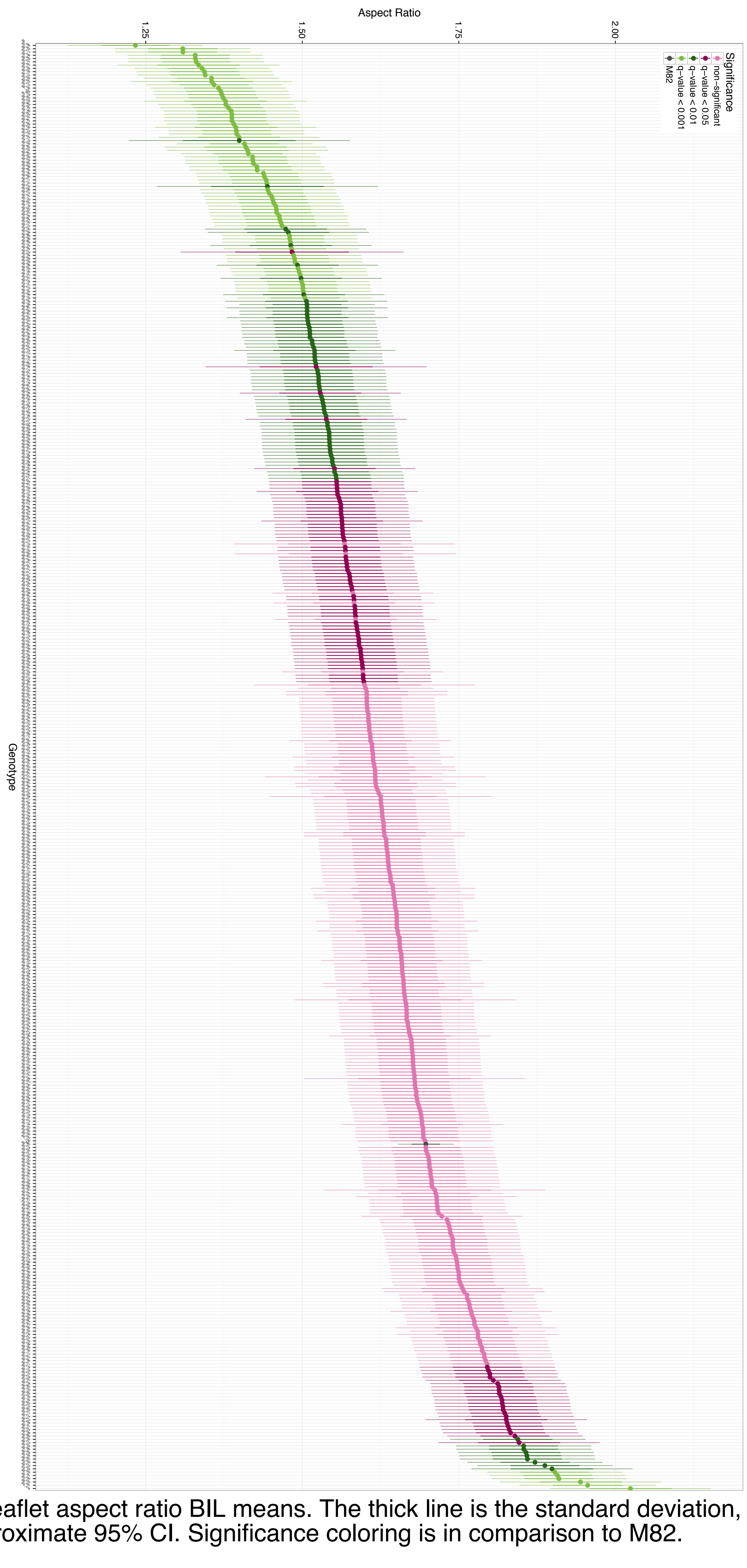

Figure S10. Leaflet aspect ratio BIL means. The thick line is the standard deviation, and the thin line is the approximate 95% CI. Significance coloring is in comparison to M82.

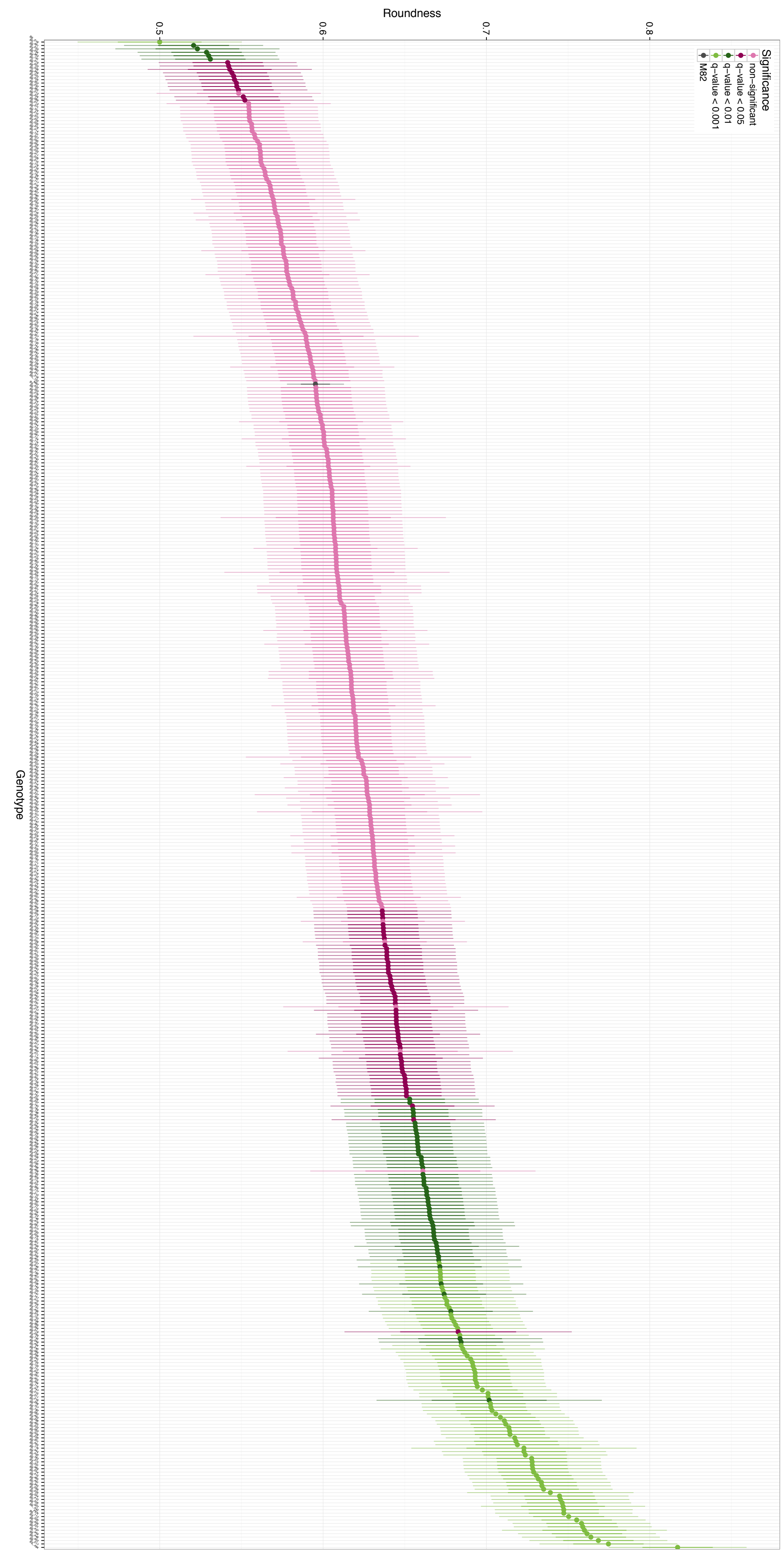

Figure S11. Leaflet roundness BIL means. The thick line is the standard deviation, and the thin line is the approximate 95% CI. Significance coloring is in comparison to M82.

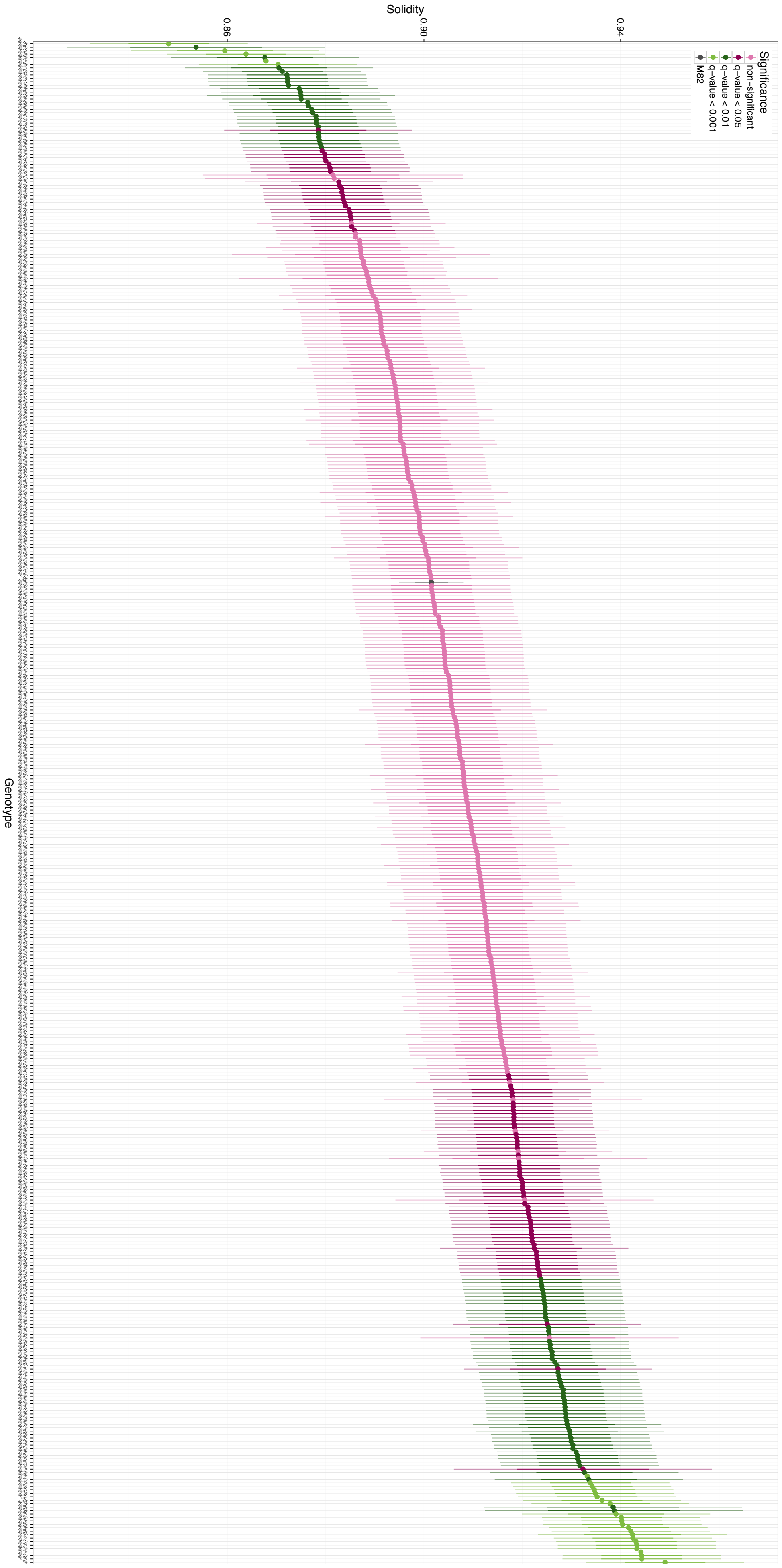

Figure S12. Leaflet solidity BIL means. The thick line is the standard deviation, and the thin line is the approximate 95% CI. Significance coloring is in comparison to M82.

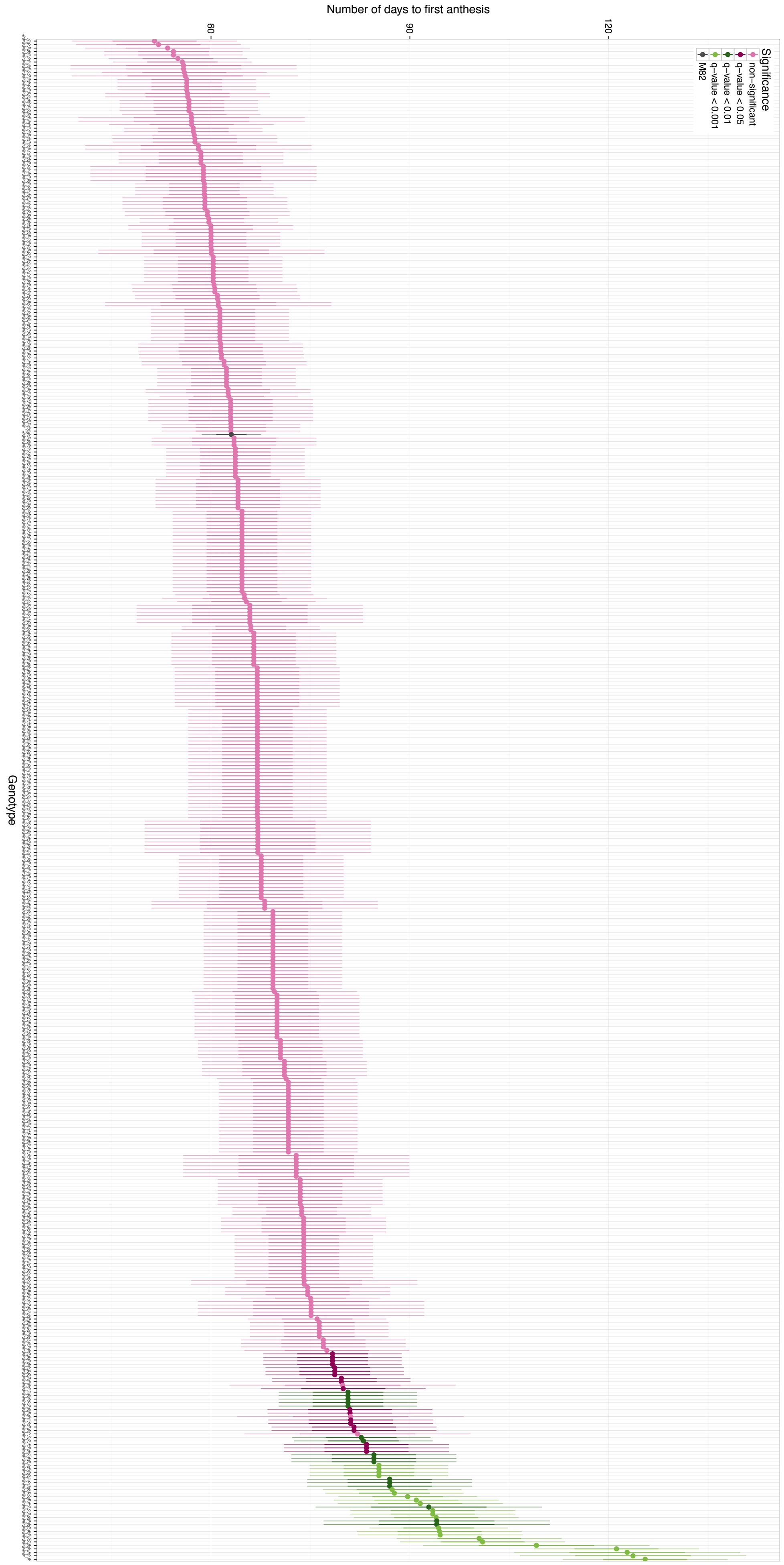

Figure S13. Flowering time BIL means. The thick line is the standard deviation, and the thin line is the approximate 95% CI. Significance coloring is in comparison to M82.

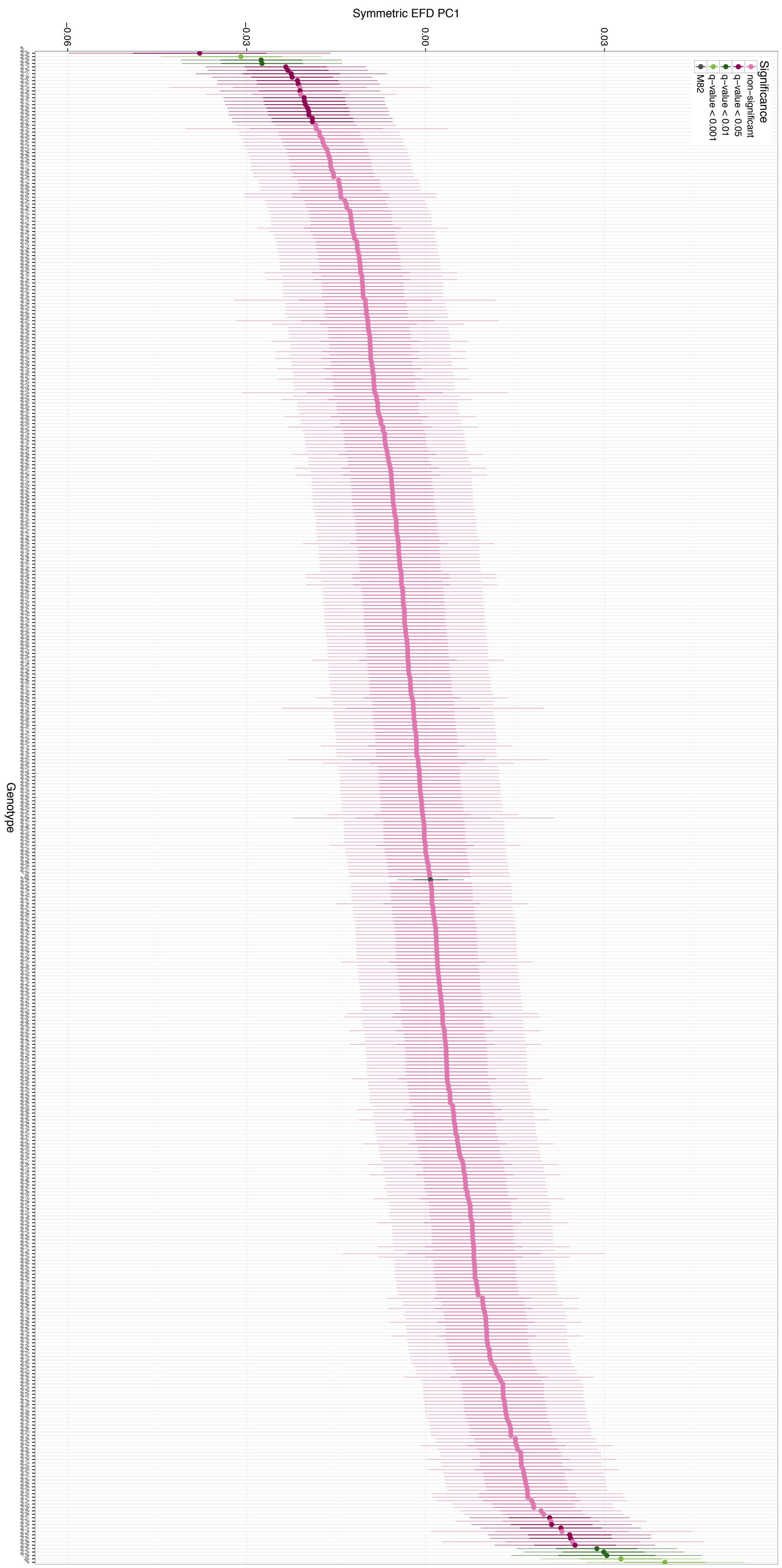

Figure S14. Leaflet symmetric EFD PC1 BIL means. The thick line is the standard deviation, and the thin line is the approximate 95% CI. Significance coloring is in comparison to M82.

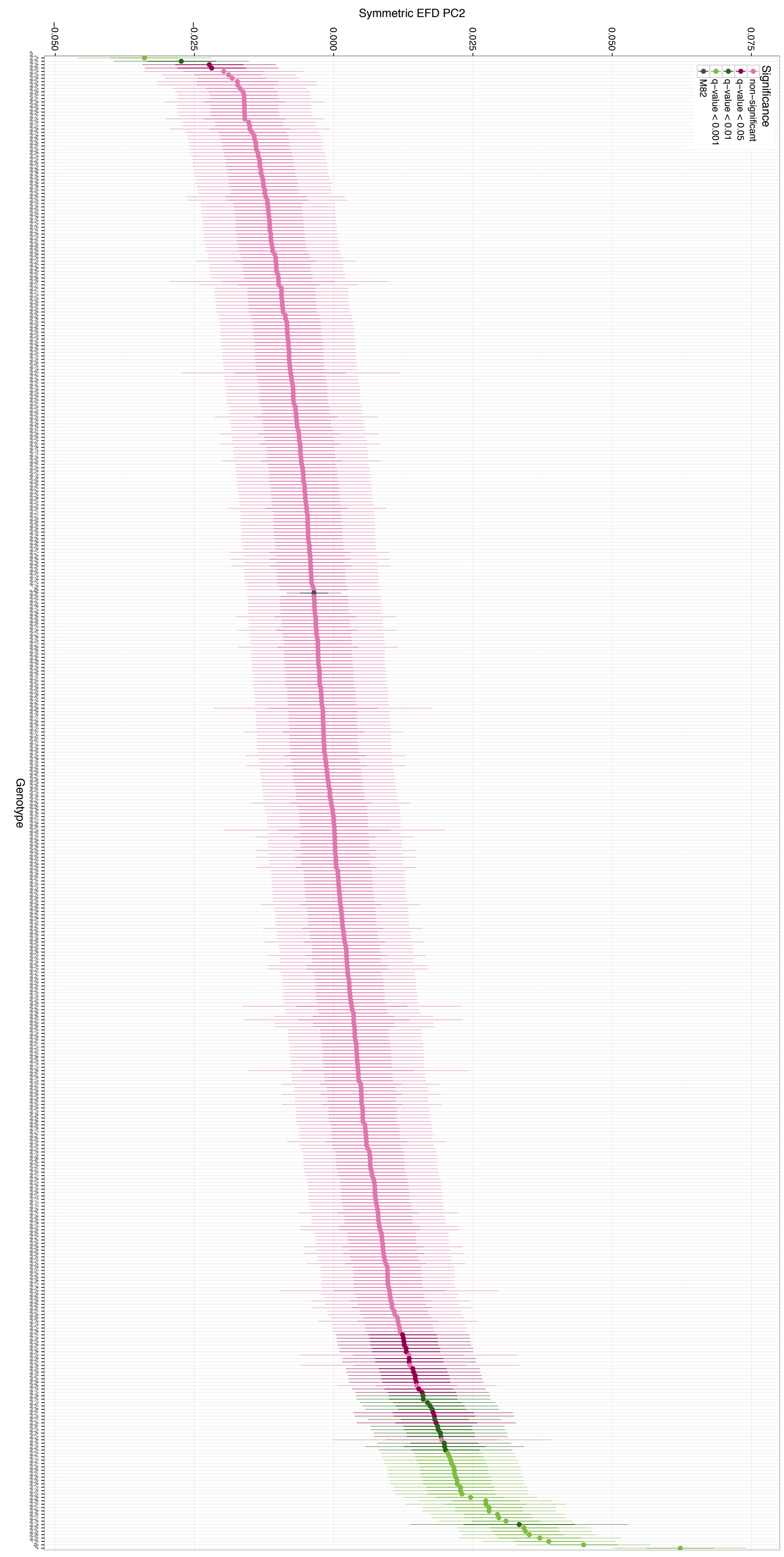

Figure S15. Leaflet symmetric EFD PC2 BIL means. The thick line is the standard deviation, and the thin line is the approximate 95% CI. Significance coloring is in comparison to M82.

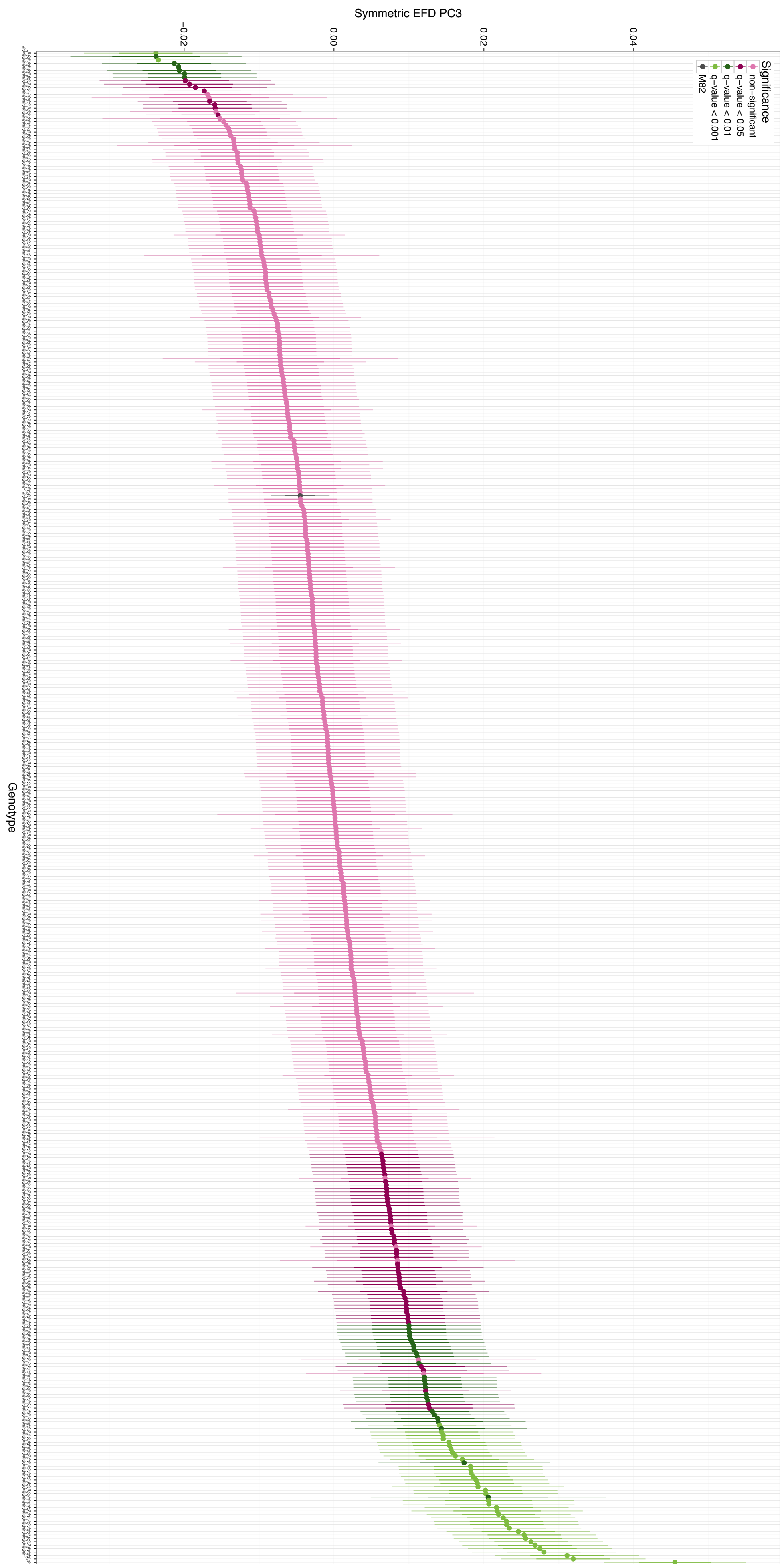

Figure S16. Leaflet symmetric EFD PC3 BIL means. The thick line is the standard deviation, and the thin line is the approximate 95% CI. Significance coloring is in comparison to M82.

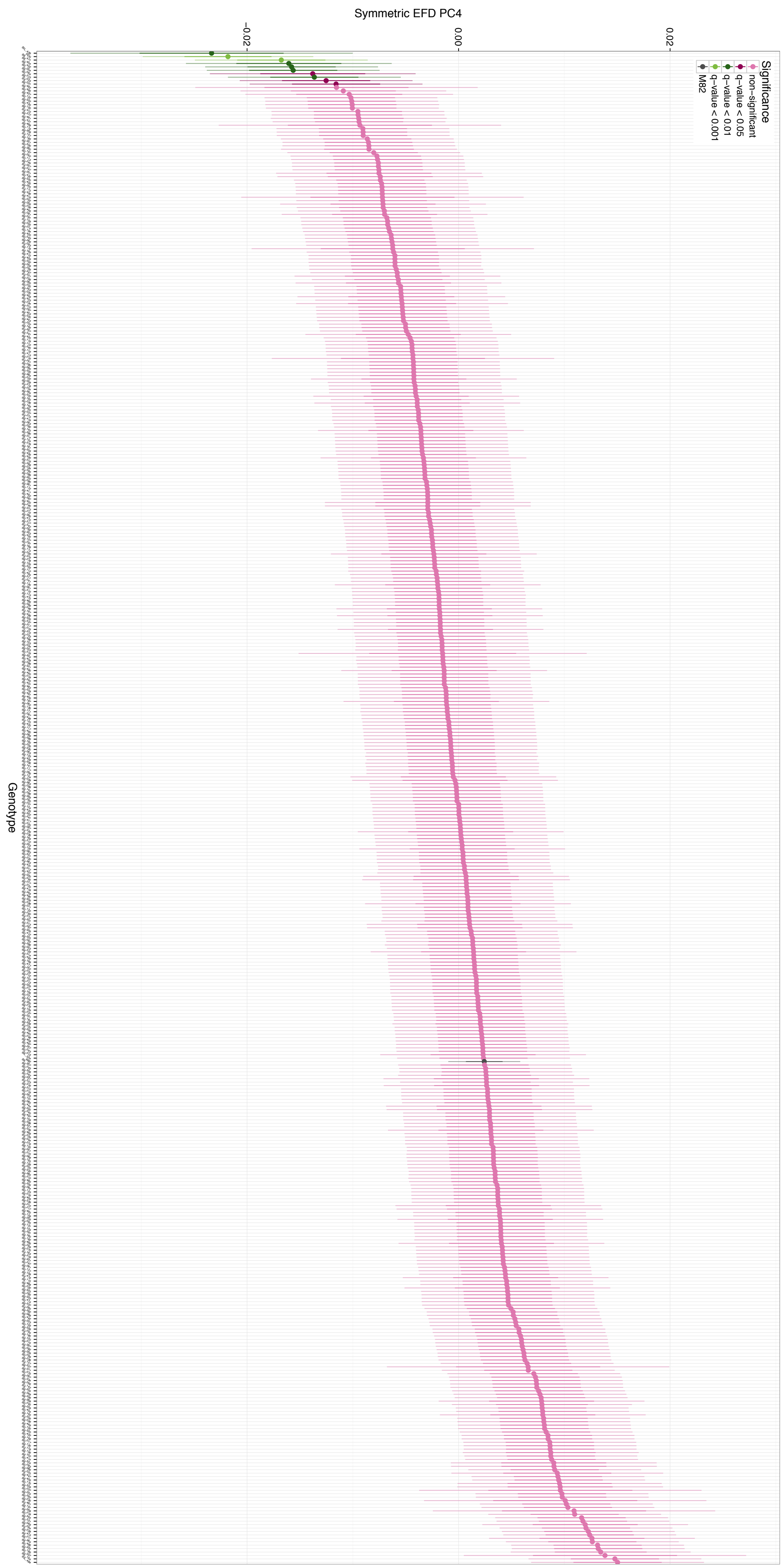

Figure S17. Leaflet symmetric EFD PC4 BIL means. The thick line is the standard deviation, and the thin line is the approximate 95% CI. Significance coloring is in comparison to M82.

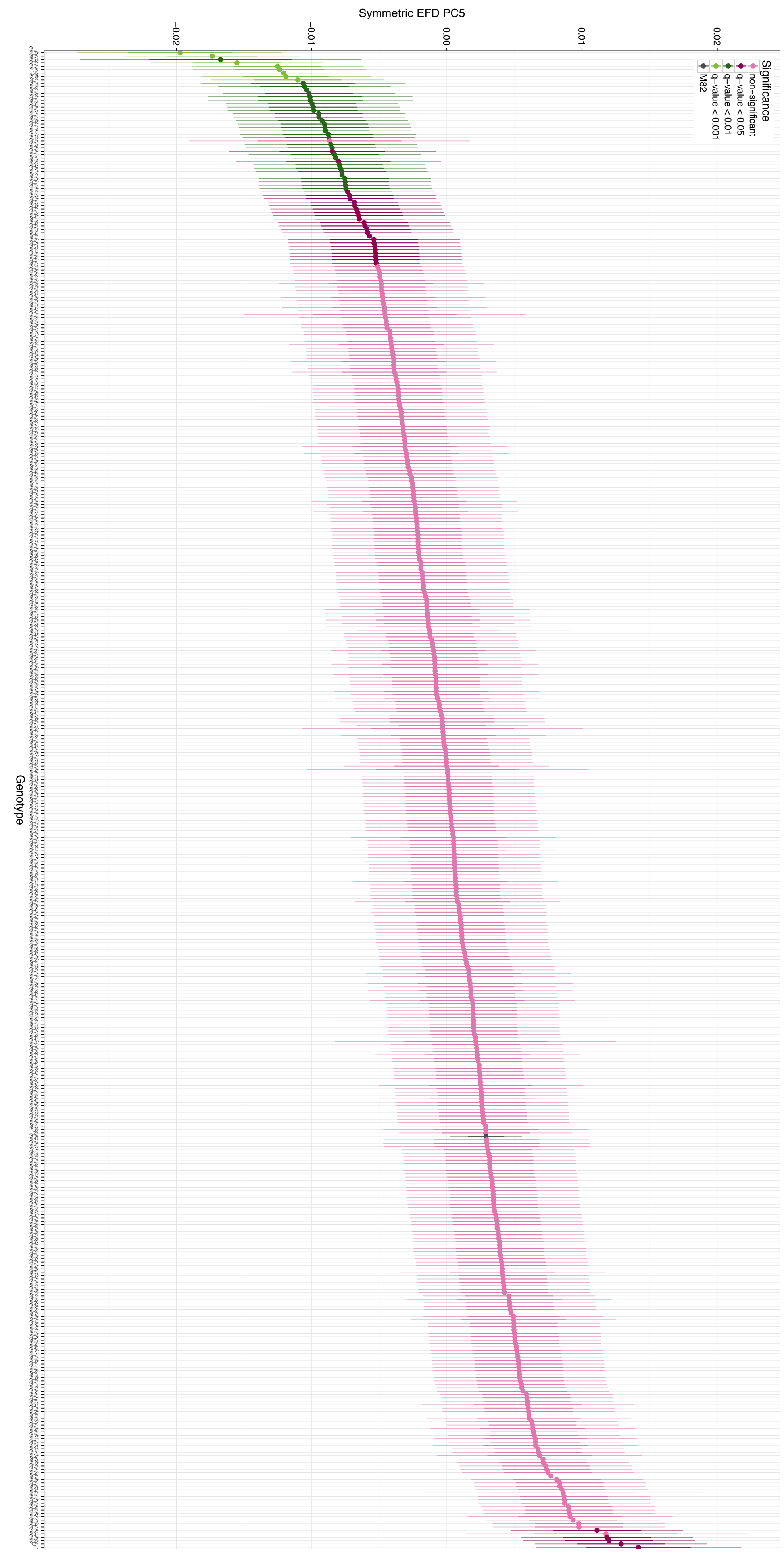

Figure S18. Leaflet symmetric EFD PC5 BIL means. The thick line is the standard deviation, and the thin line is the approximate 95% CI. Significance coloring is in comparison to M82.

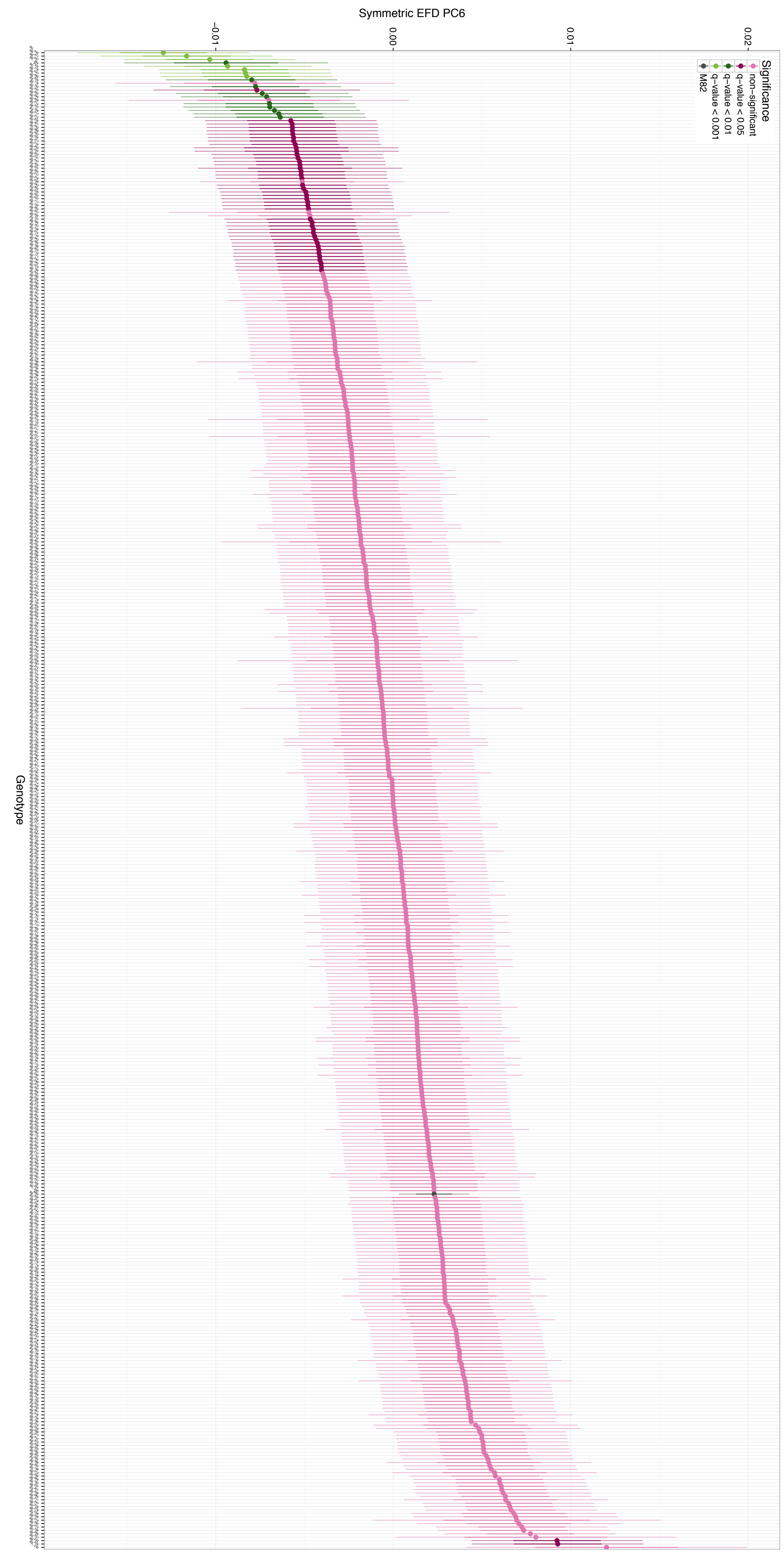

Figure S19. Leaflet symmetric EFD PC6 BIL means. The thick line is the standard deviation, and the thin line is the approximate 95% CI. Significance coloring is in comparison to M82.

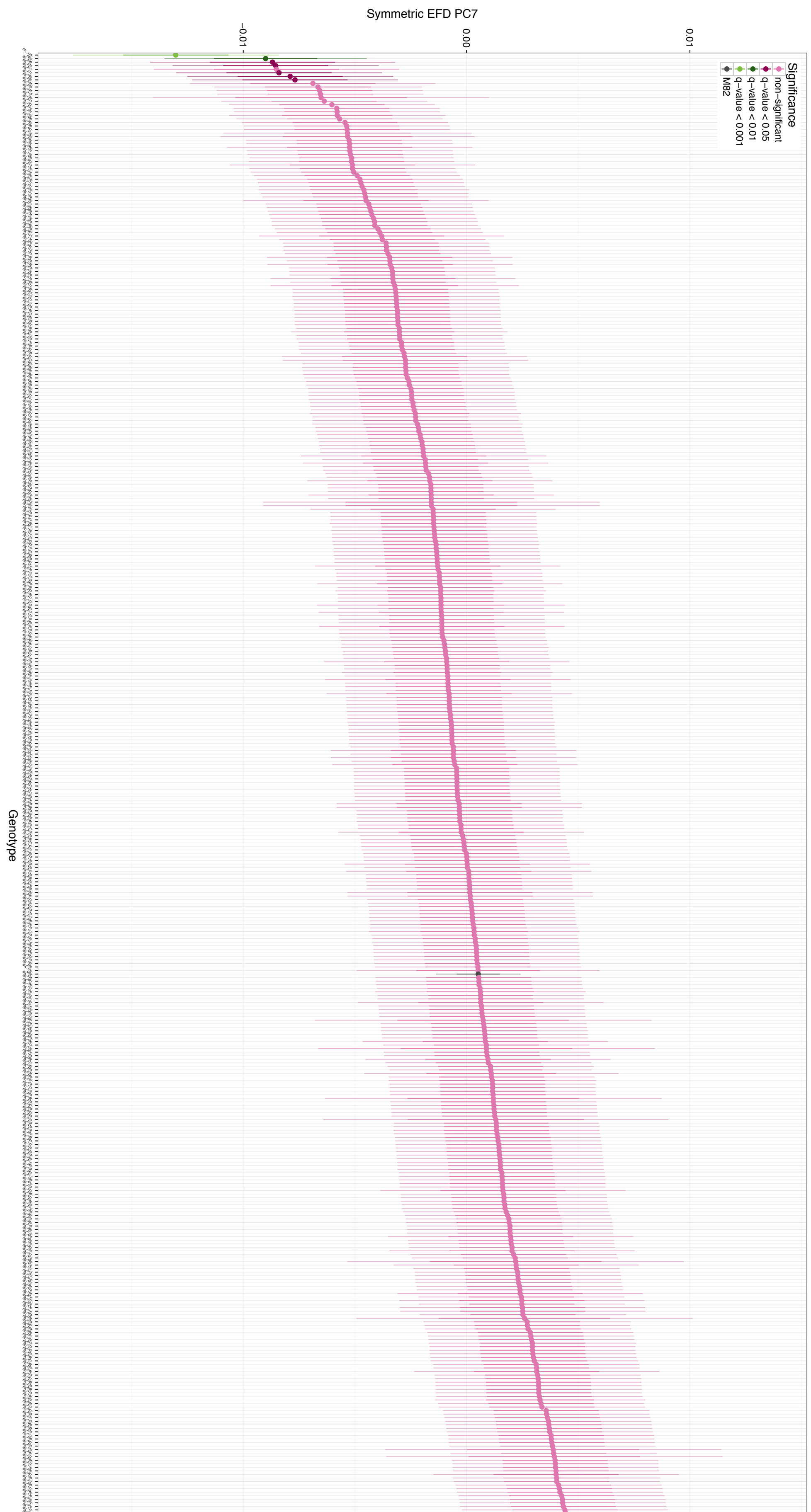

Figure S20. Leaflet symmetric EFD PC7 BIL means. The thick line is the standard deviation, and the thin line is the approximate 95% CI. Significance coloring is in comparison to M82.

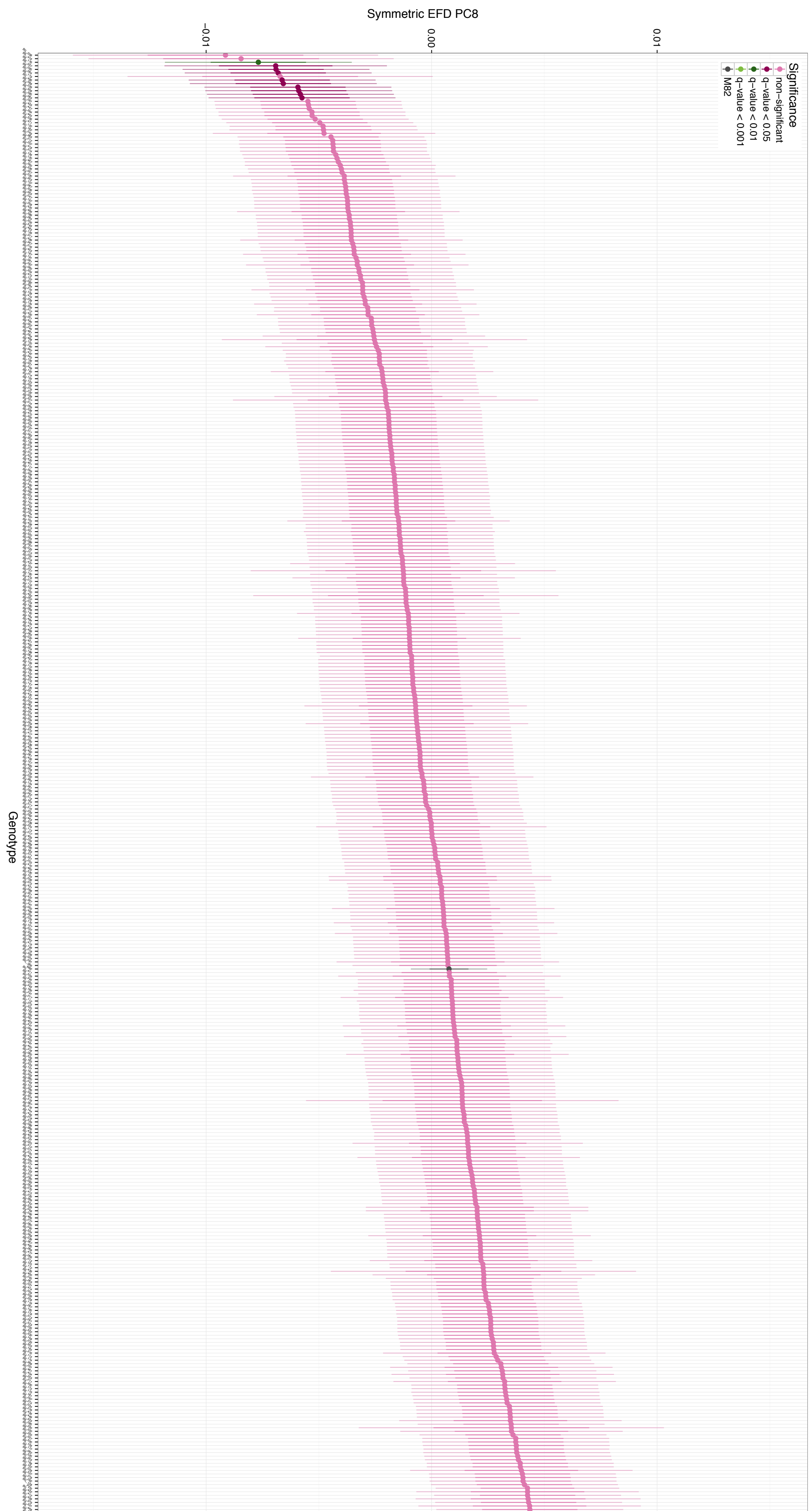

Figure S21. Leaflet symmetric EFD PC8 BIL means. The thick line is the standard deviation, and the thin line is the approximate 95% CI. Significance coloring is in comparison to M82.

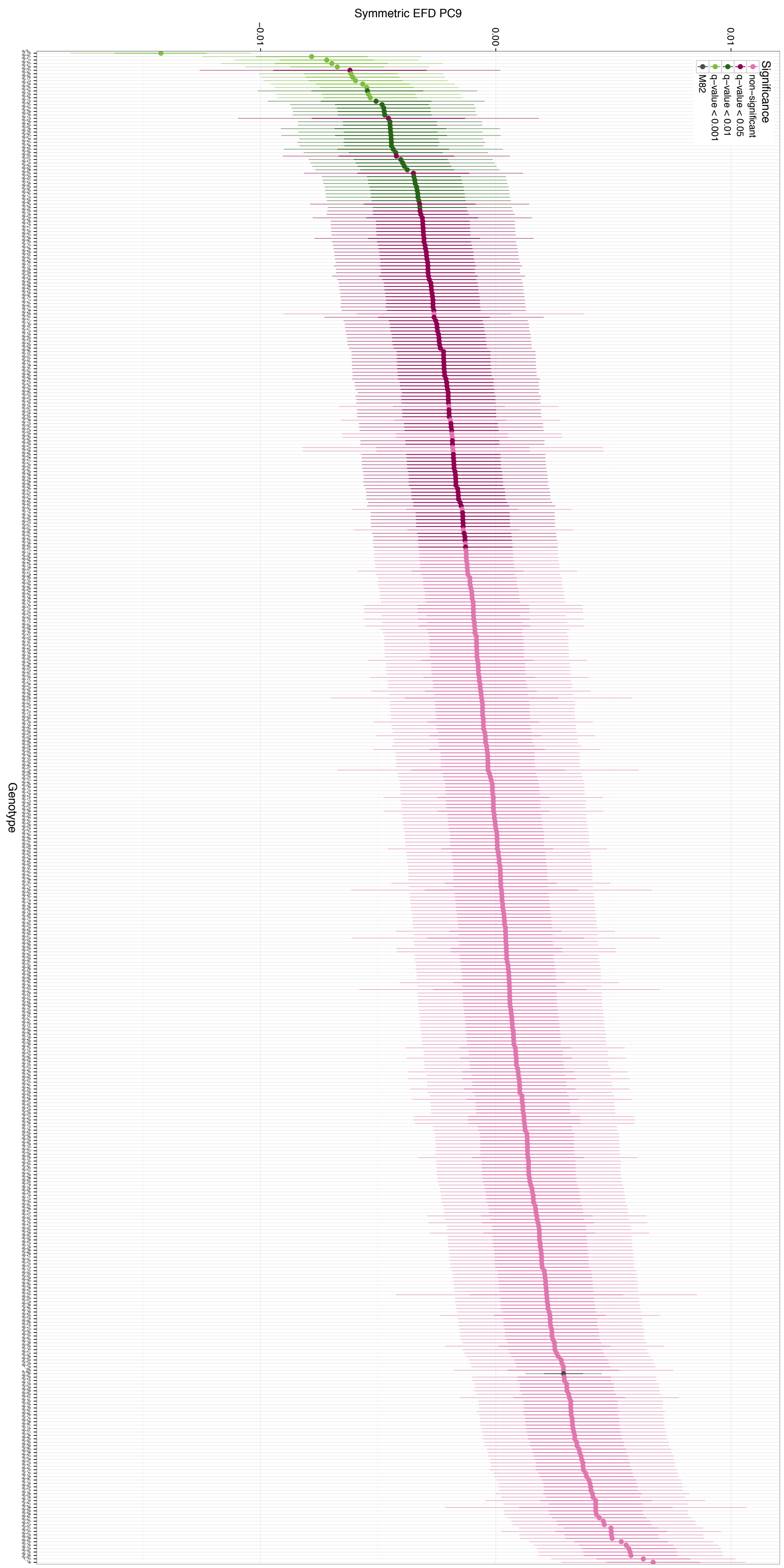

Figure S22. Leaflet symmetric EFD PC9 BIL means. The thick line is the standard deviation, and the thin line is the approximate 95% CI. Significance coloring is in comparison to M82.

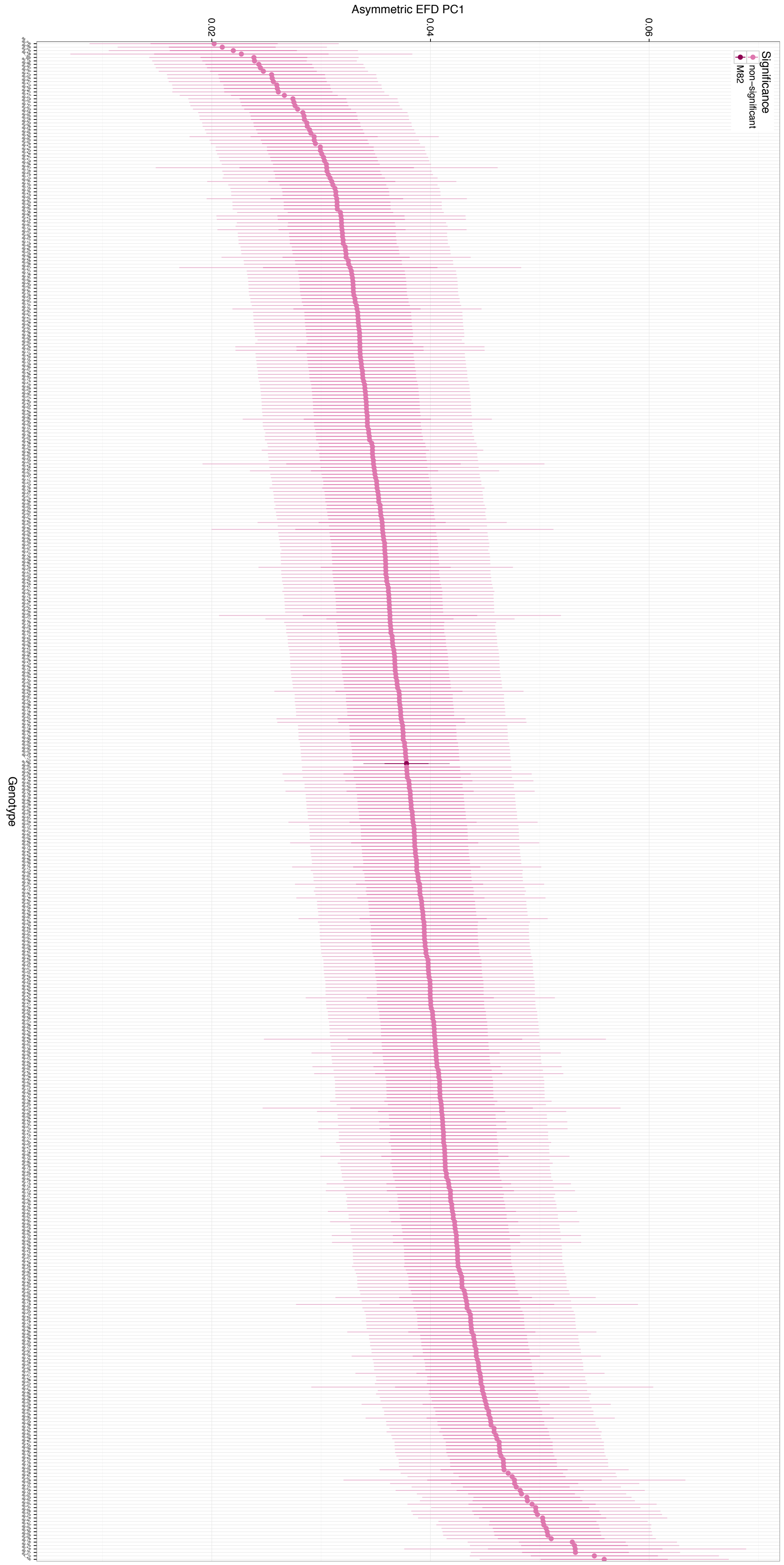

Figure S23. Leaflet asymmetric EFD PC1 BIL means. The thick line is the standard deviation, and the thin line is the approximate 95% CI. Significance coloring is in comparison to M82.

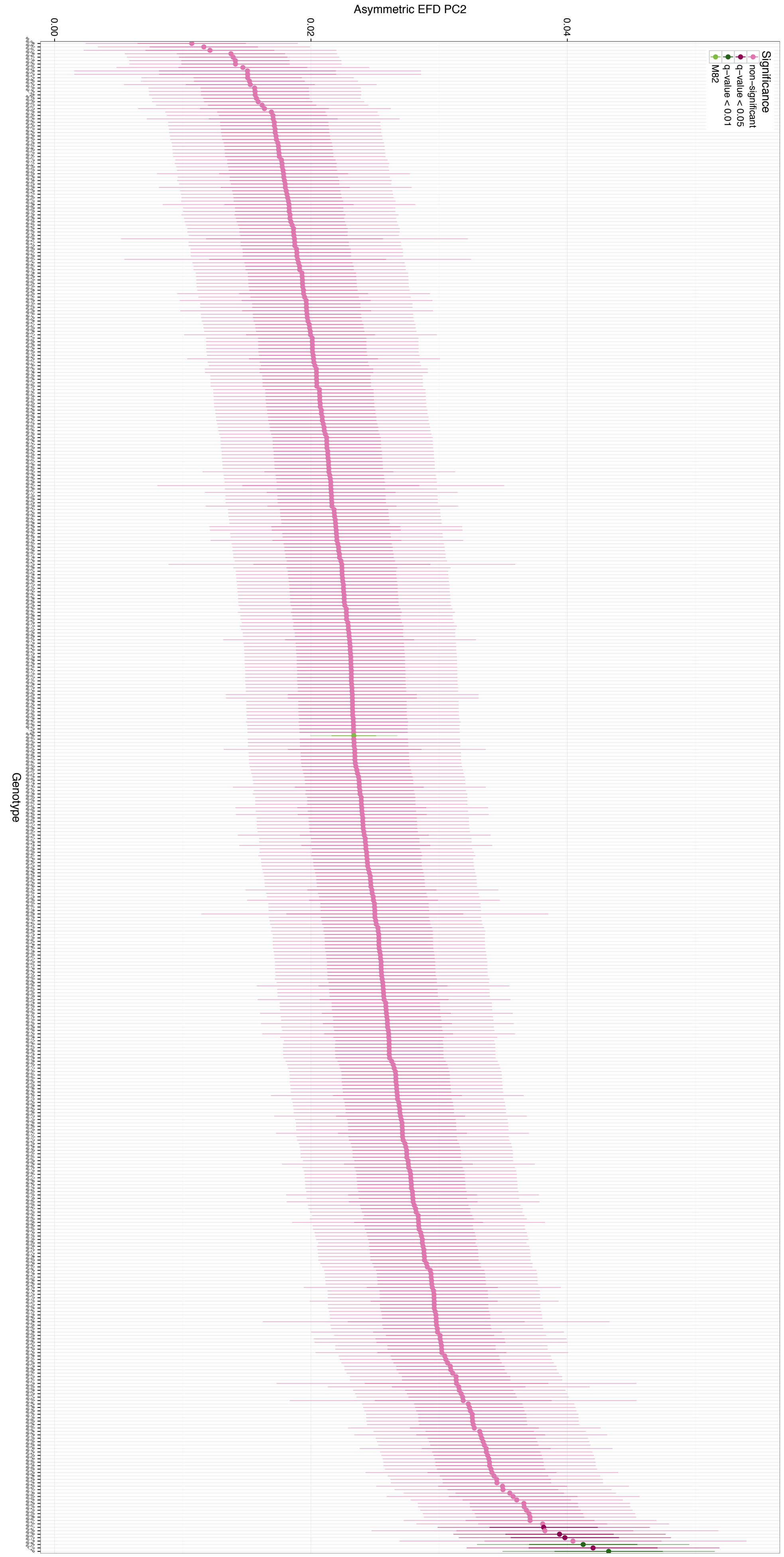

Figure S24. Leaflet asymmetric EFD PC2 BIL means. The thick line is the standard deviation, and the thin line is the approximate 95% CI. Significance coloring is in comparison to M82.

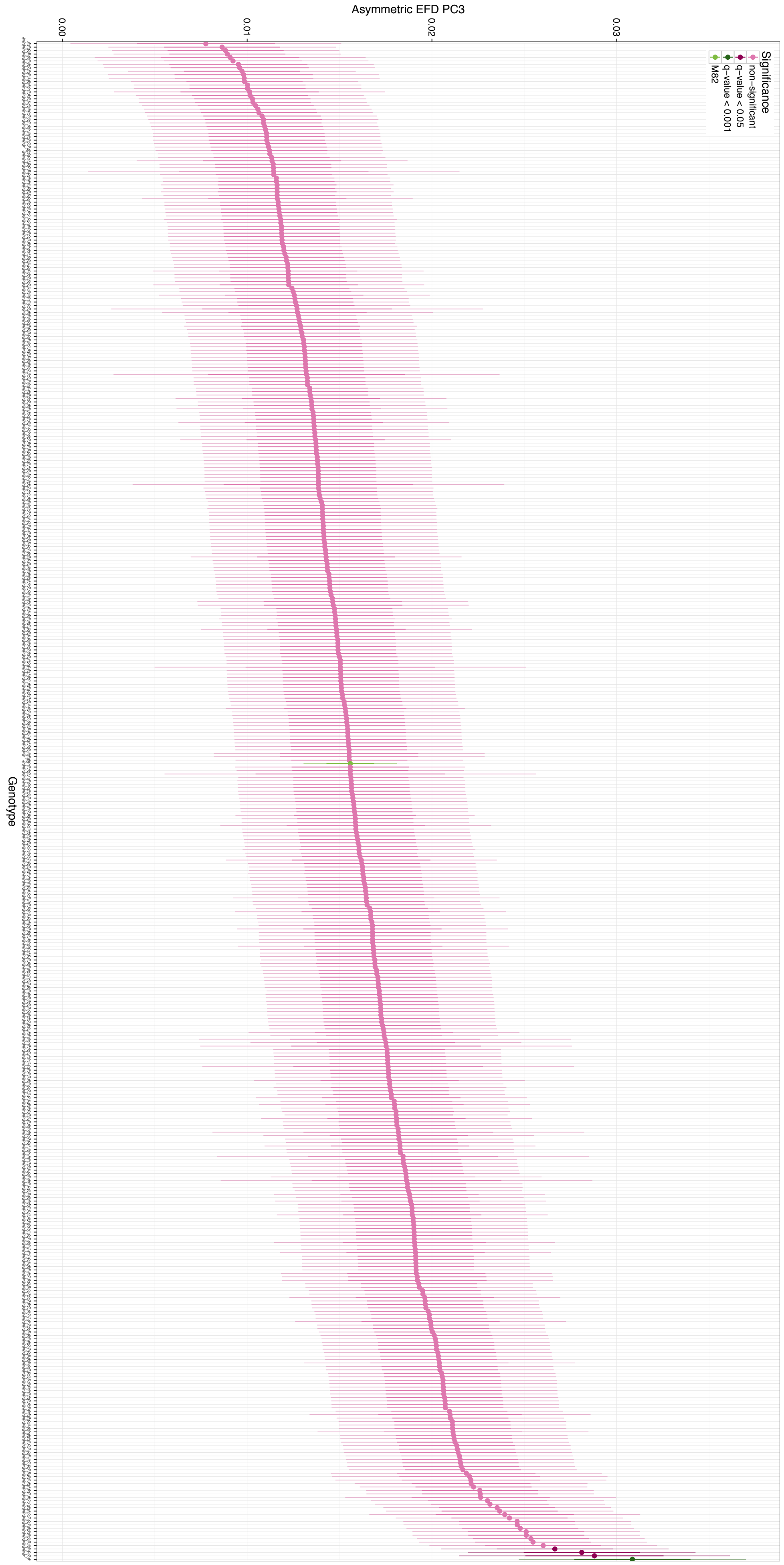

Figure S25. Leaflet asymmetric EFD PC3 BIL means. The thick line is the standard deviation, and the thin line is the approximate 95% CI. Significance coloring is in comparison to M82.

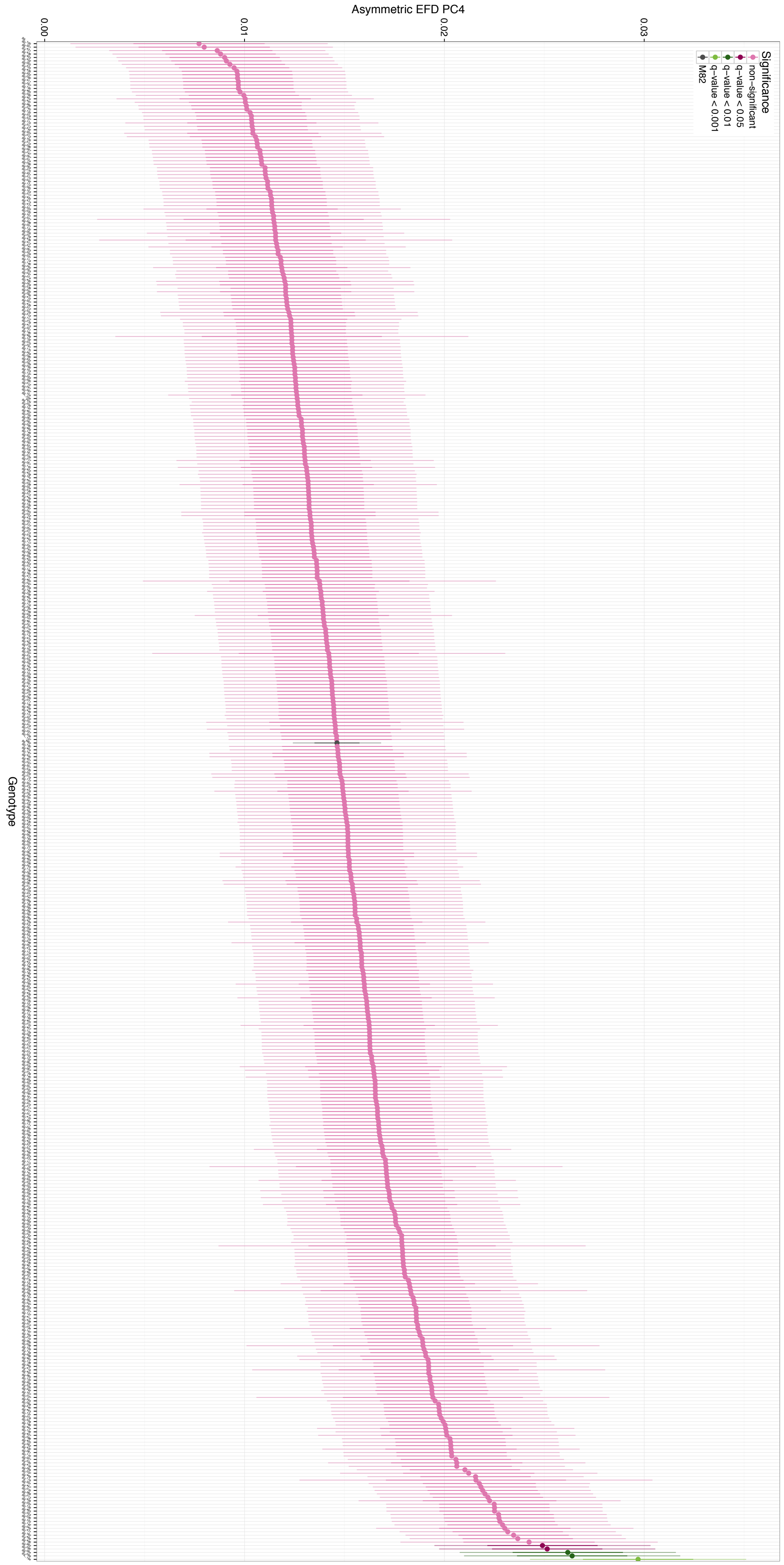

Figure S26. Leaflet asymmetric EFD PC4 BIL means. The thick line is the standard deviation, and the thin line is the approximate 95% CI. Significance coloring is in comparison to M82.

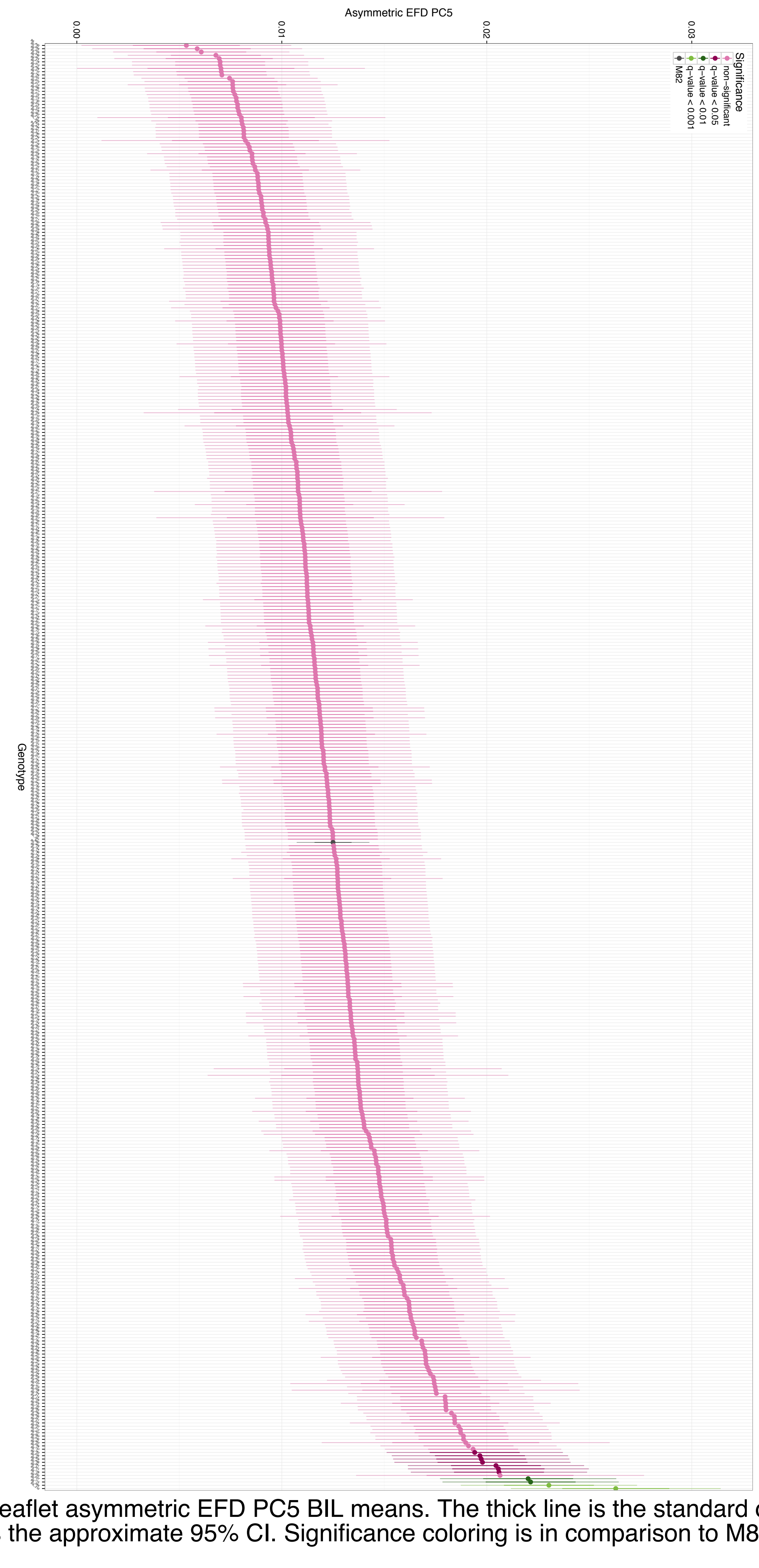

Figure S27. Leaflet asymmetric EFD PC5 BIL means. The thick line is the standard deviation, and the thin line is the approximate 95% CI. Significance coloring is in comparison to M82.

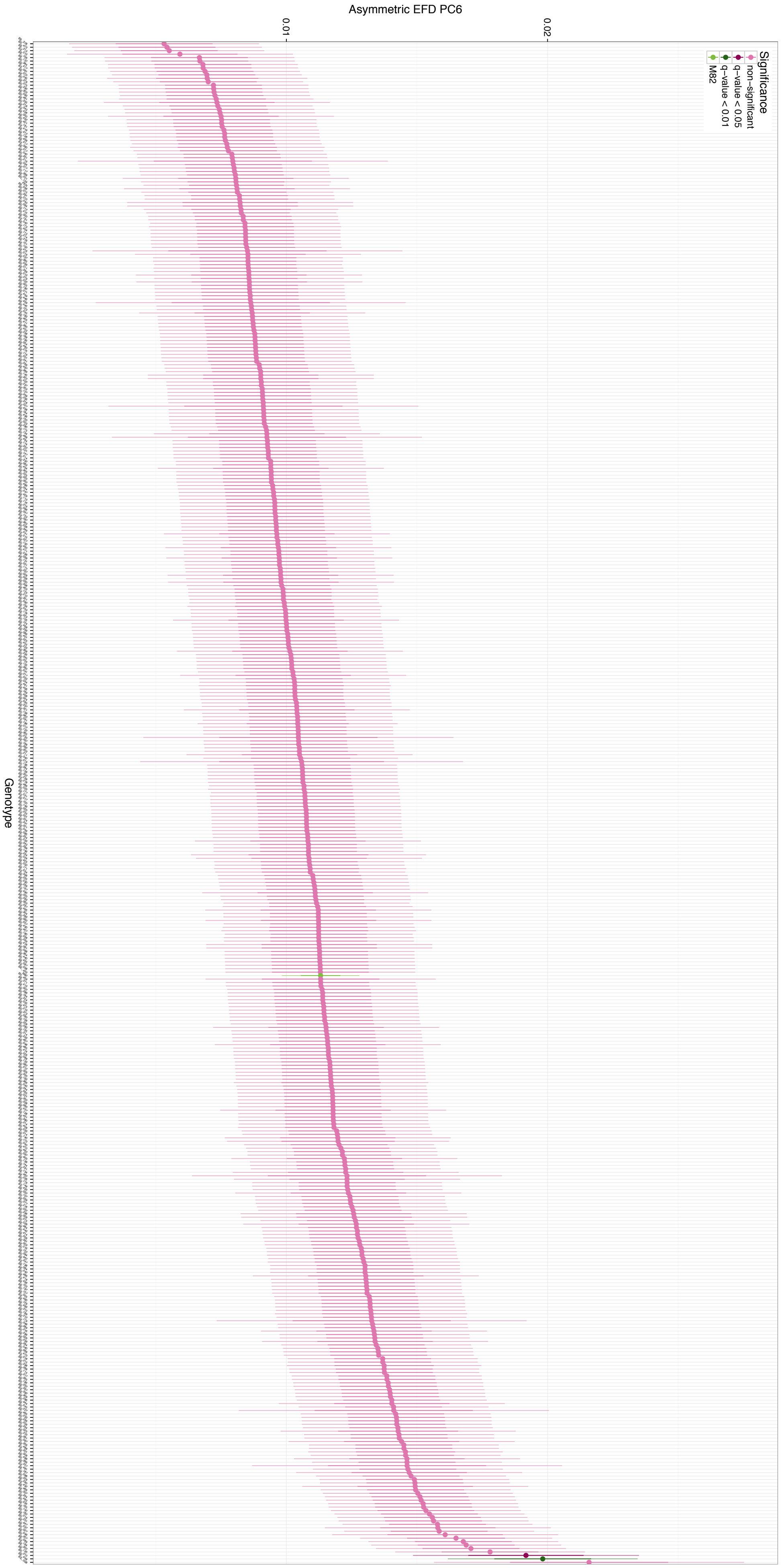

Figure S28. Leaflet asymmetric EFD PC6 BIL means. The thick line is the standard deviation, and the thin line is the approximate 95% CI. Significance coloring is in comparison to M82.

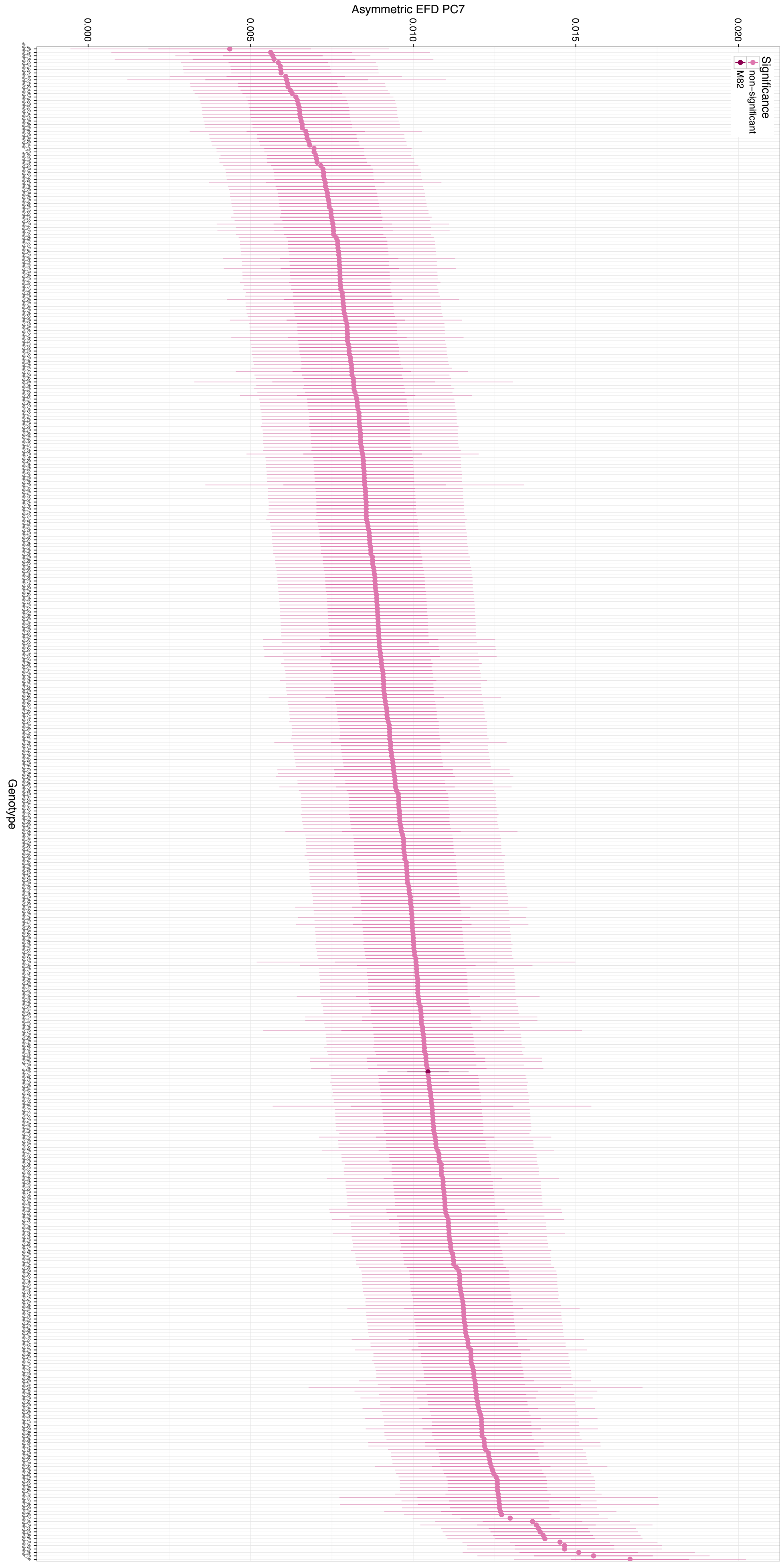

Figure S29. Leaflet asymmetric EFD PC7 BIL means. The thick line is the standard deviation, and the thin line is the approximate 95% CI. Significance coloring is in comparison to M82.

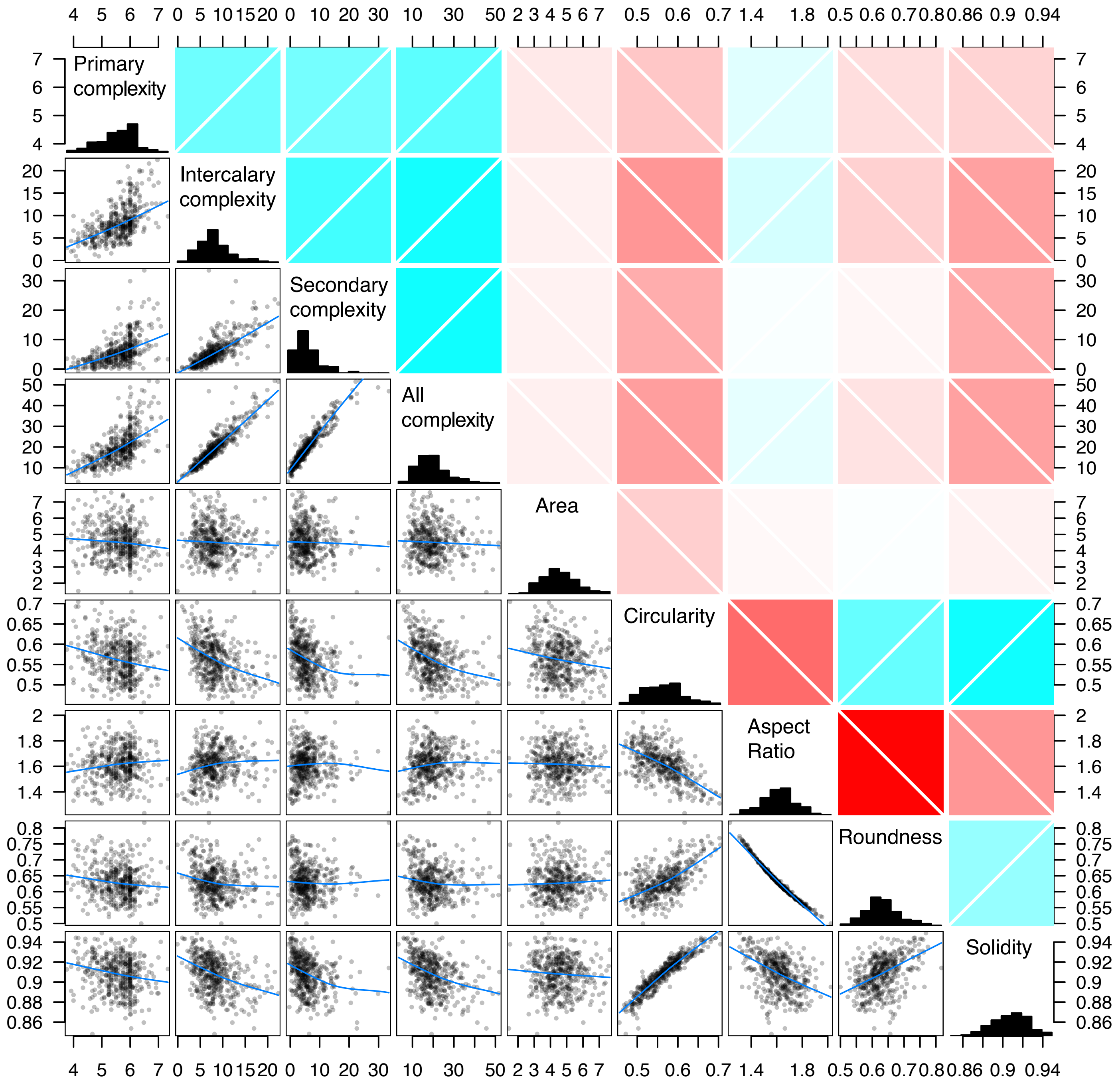

Figure S30. Leaf complexity and leaflet shape trait distributions and correlations. The diagonal shows single trait distributions and their names. The lower triangle shows bivariate scatter plots of pairwise trait combinations; the blue curves are the LOESS regression fit of the bivariate data. The upper triangle shows the strength and sign of correlation among pairs of traits; blue indicates positive correlation, red negative correlation, and the intensity of the color represents the magnitude of the correlation.

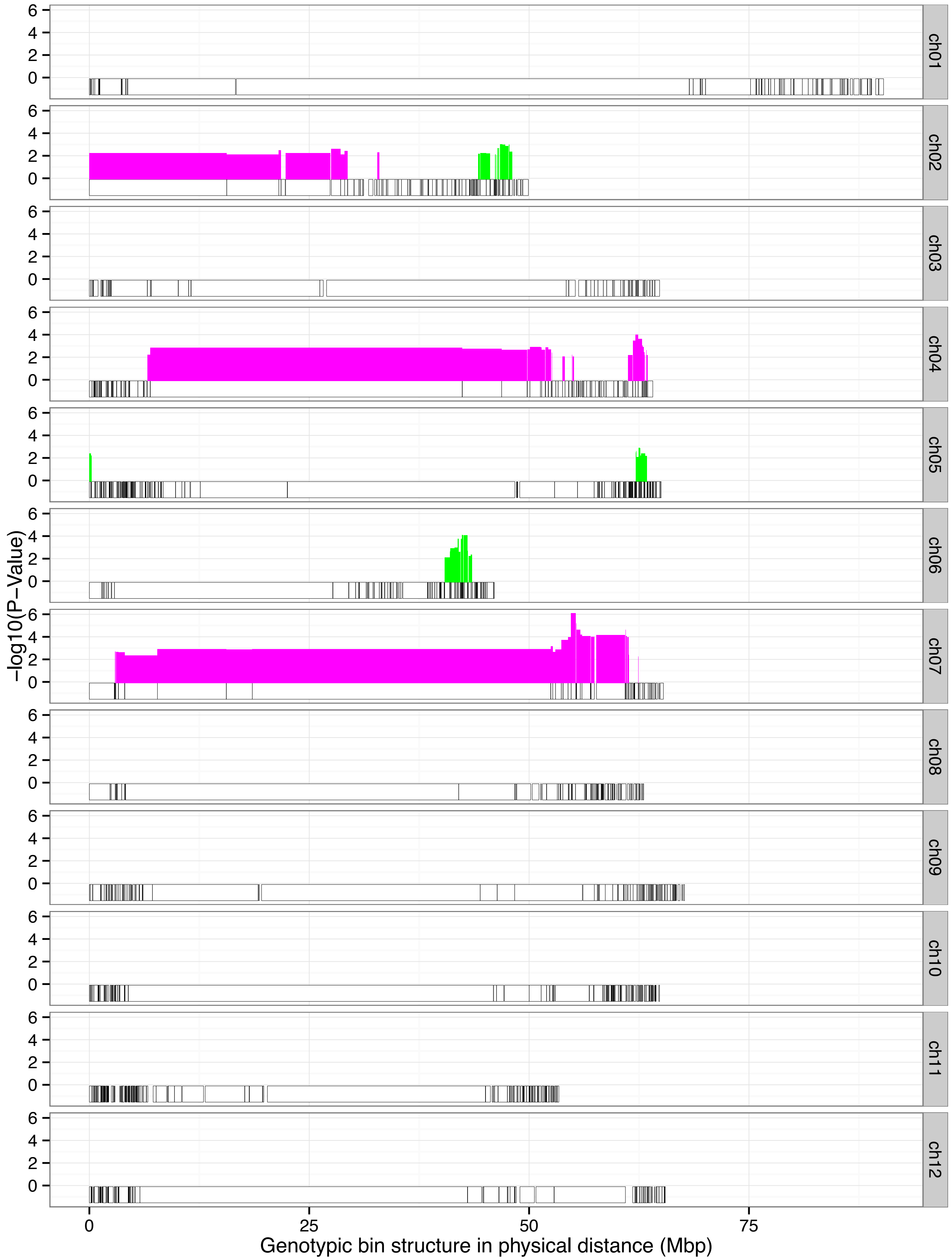

Figure S31. Primary leaf complexity marginal regression QTL mapping results. QTL sign (phenotypic effect) is denoted by color: positive = green, negative = magenta.

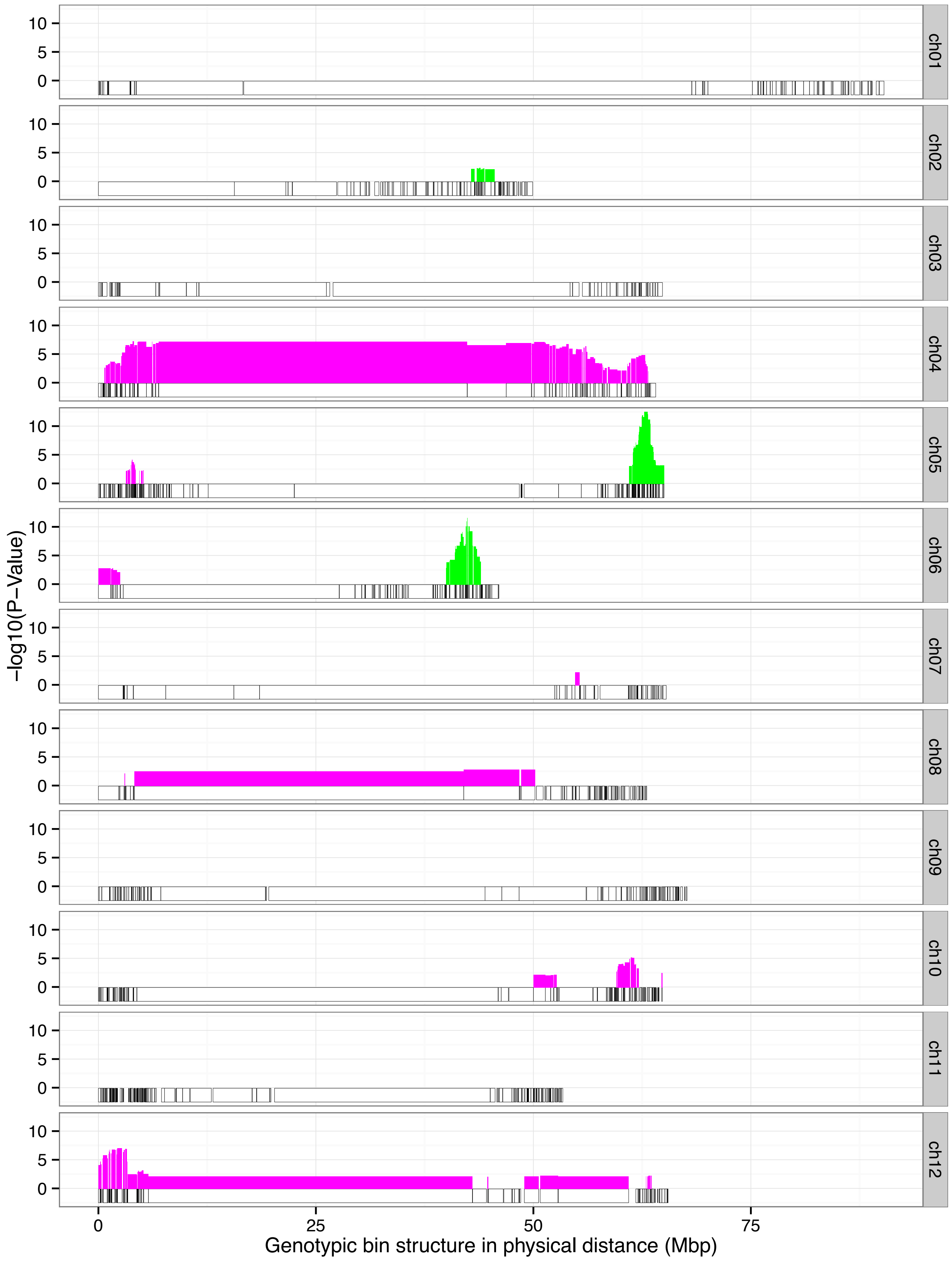

Figure S32. Intercalary leaf complexity marginal regression QTL mapping results. QTL sign (phenotypic effect) is denoted by color: positive = green, negative = magenta.

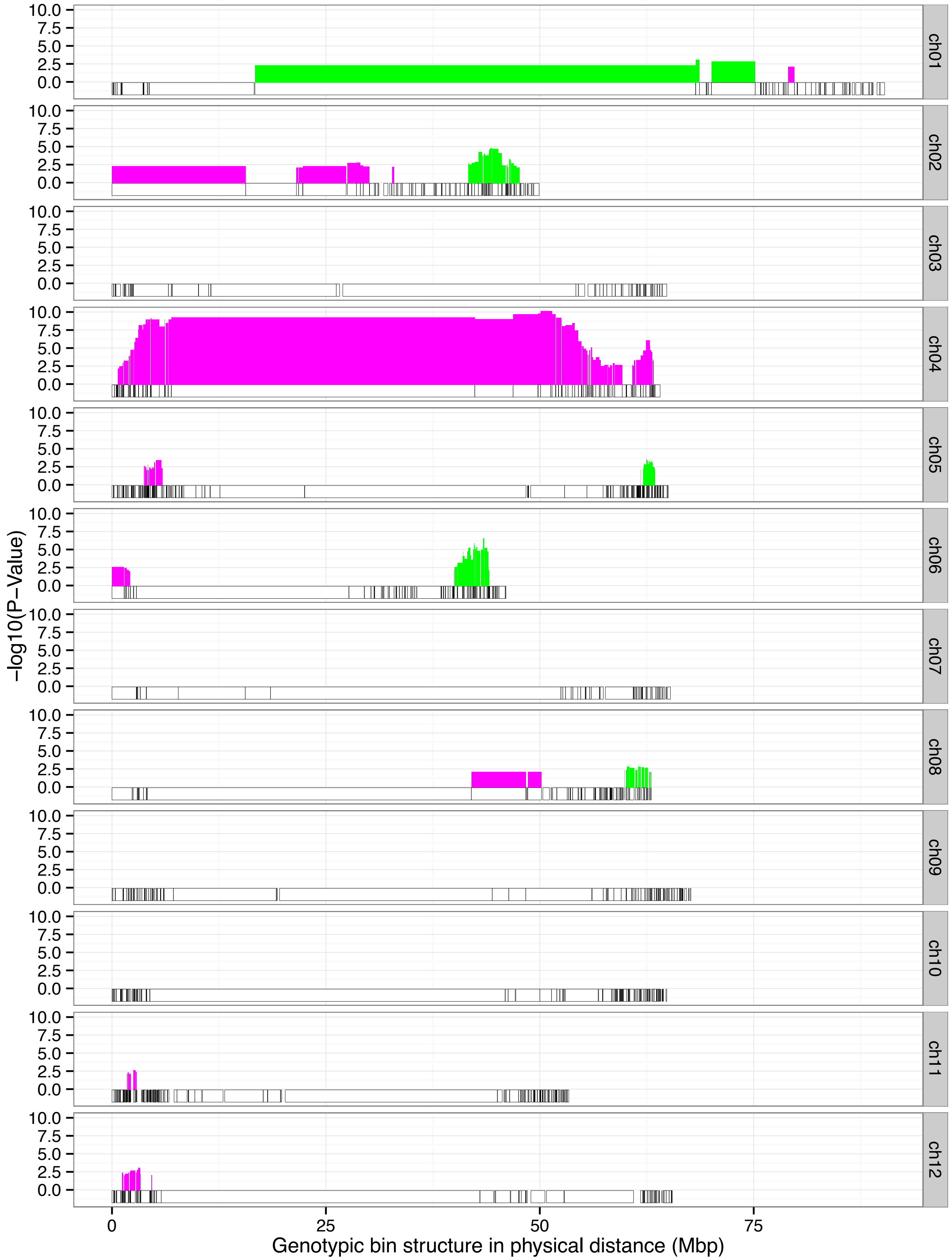

Figure S33. Secondary leaf complexity marginal regression QTL mapping results. QTL sign (phenotypic effect) is denoted by color: positive = green, negative = magenta.

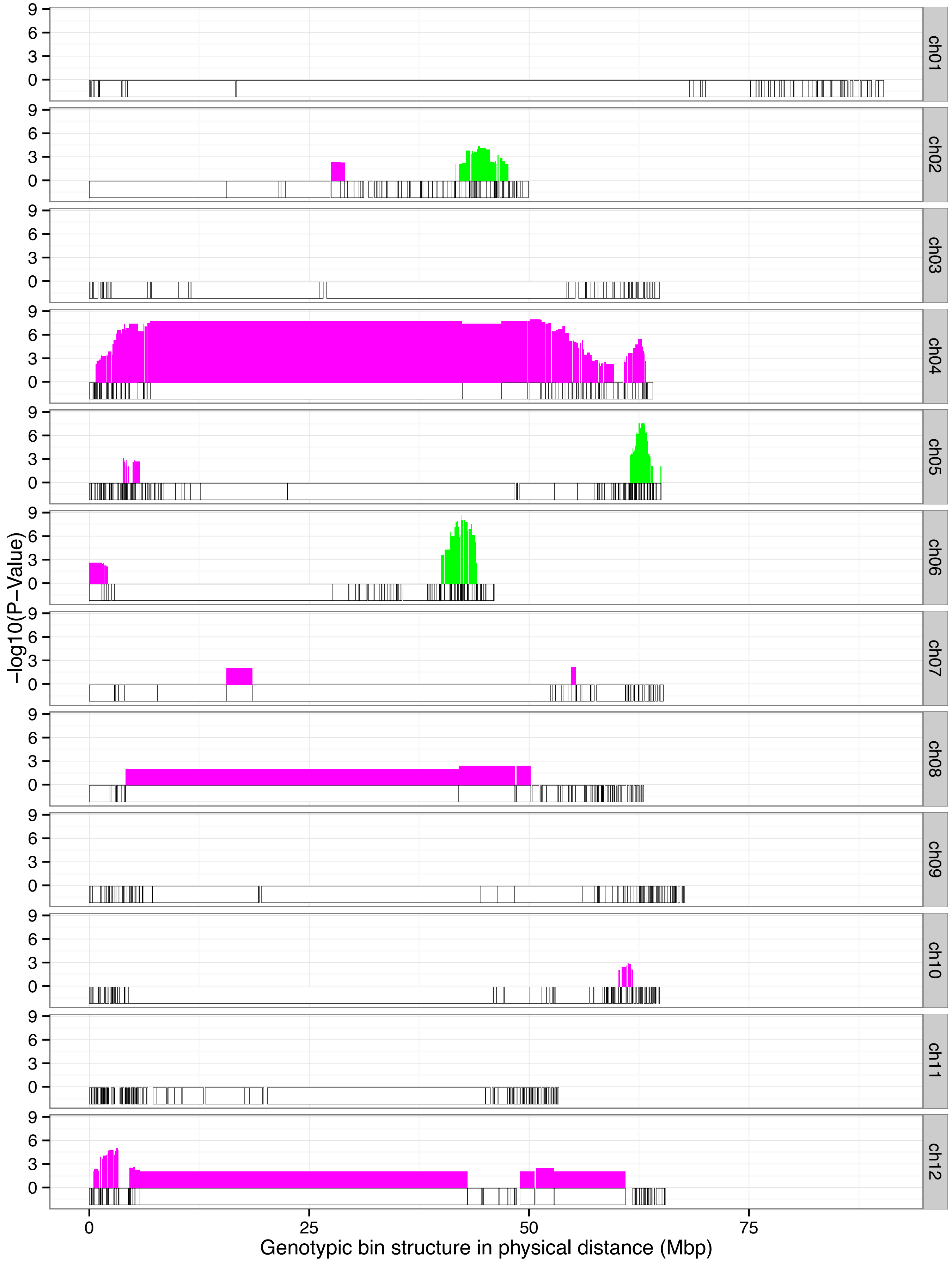

Figure S34. Total leaf complexity marginal regression QTL mapping results. QTL sign (phenotypic effect) is denoted by color: positive = green, negative = magenta.

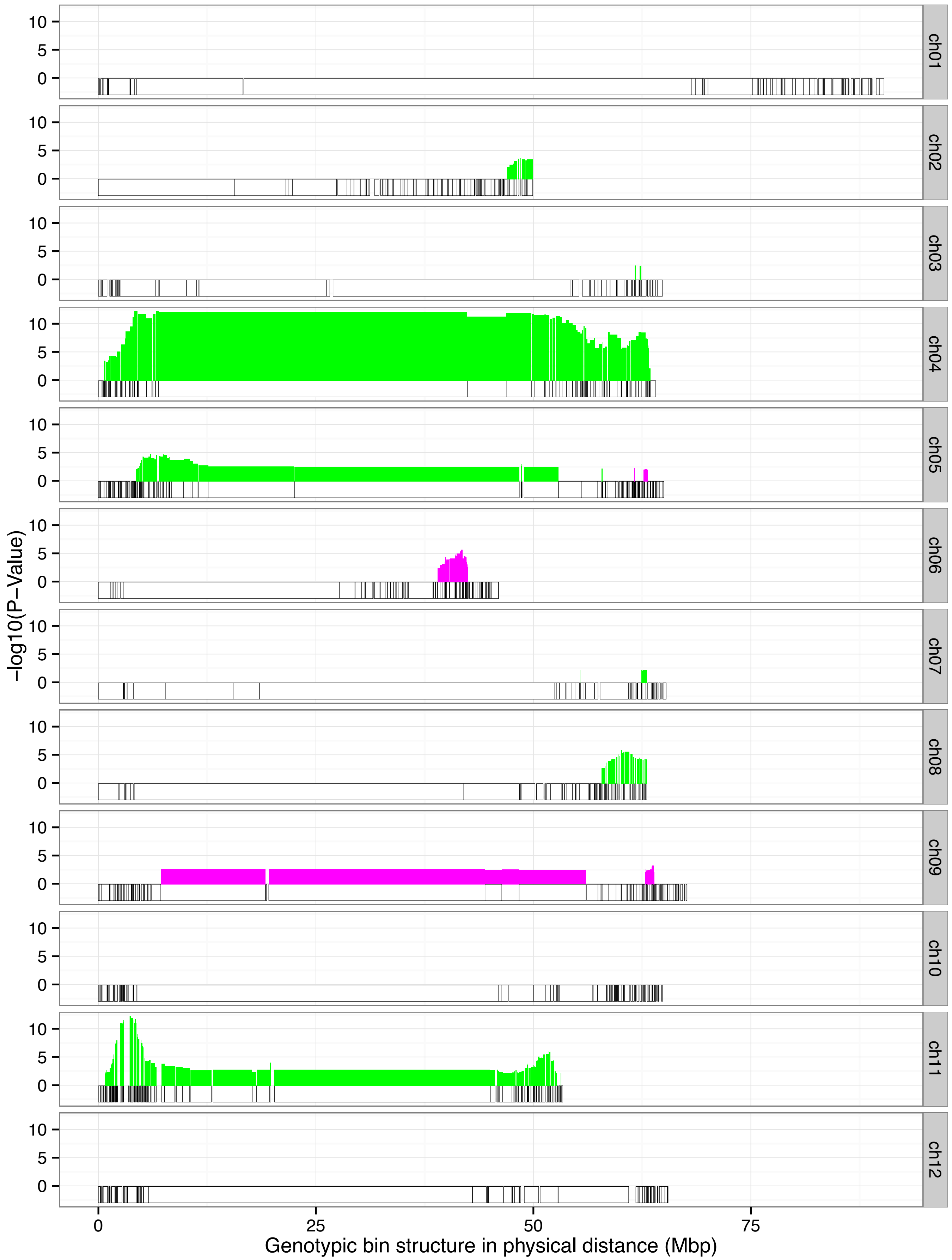

Figure S35. Leaflet circularity marginal regression QTL mapping results. QTL sign (phenotypic effect) is denoted by color: positive = green, negative = magenta.

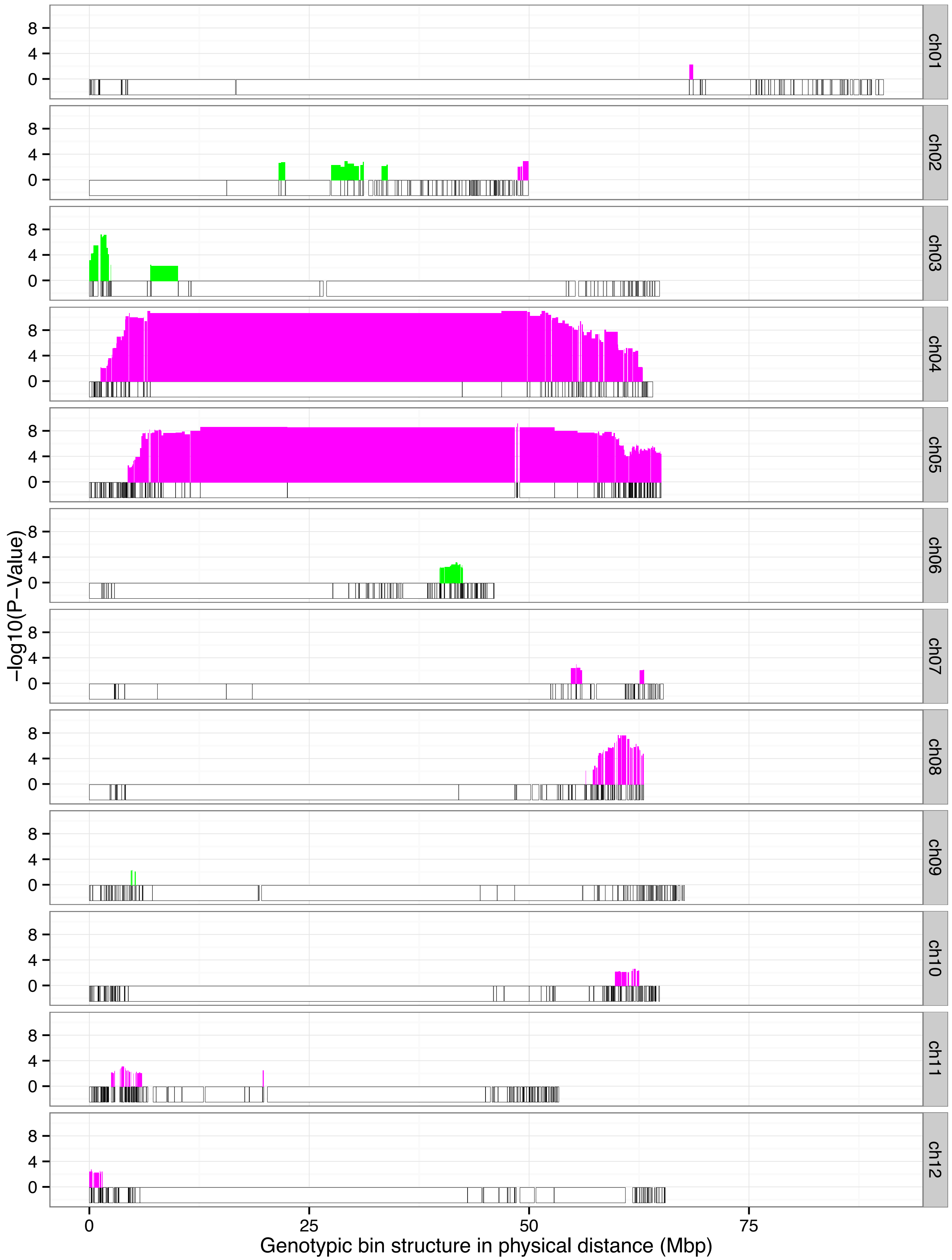

Figure S36. Leaflet aspect ratio marginal regression QTL mapping results. QTL sign (phenotypic effect) is denoted by color: positive = green, negative = magenta.

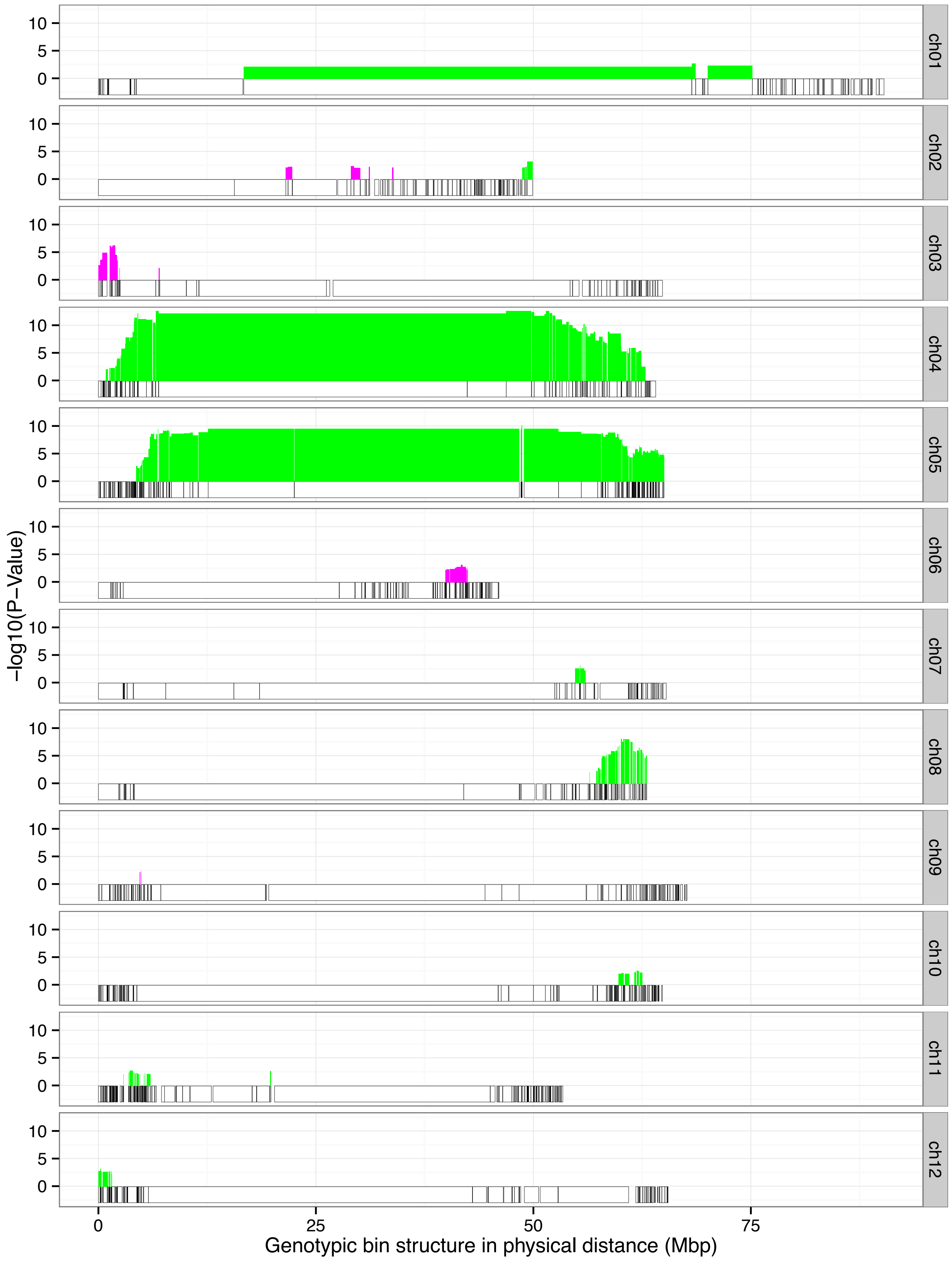

Figure S37. Leaflet roundness marginal regression QTL mapping results. QTL sign (phenotypic effect) is denoted by color: positive = green, negative = magenta.

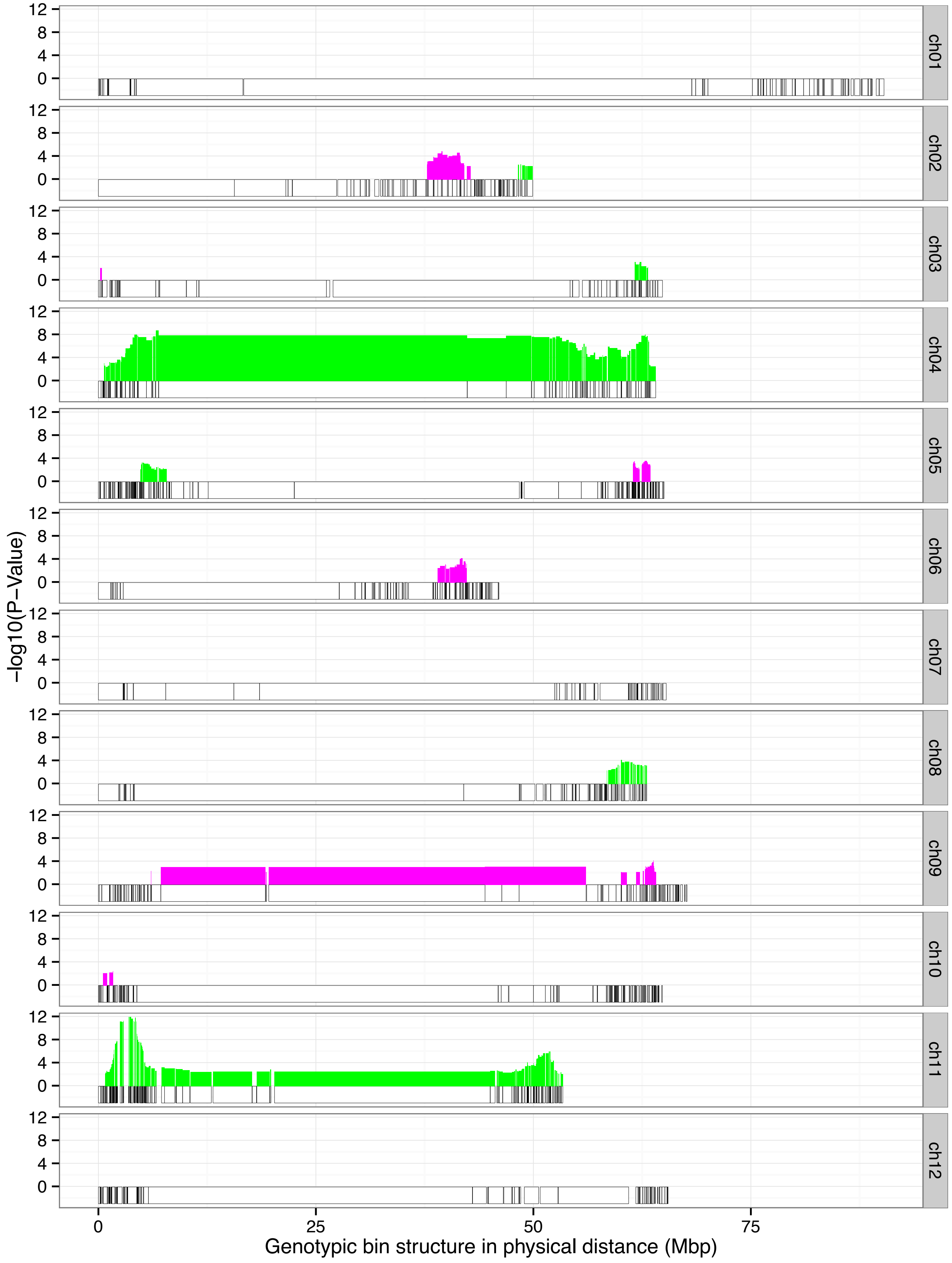

Figure S38. Leaflet solidity marginal regression QTL mapping results. QTL sign (phenotypic effect) is denoted by color: positive = green, negative = magenta.

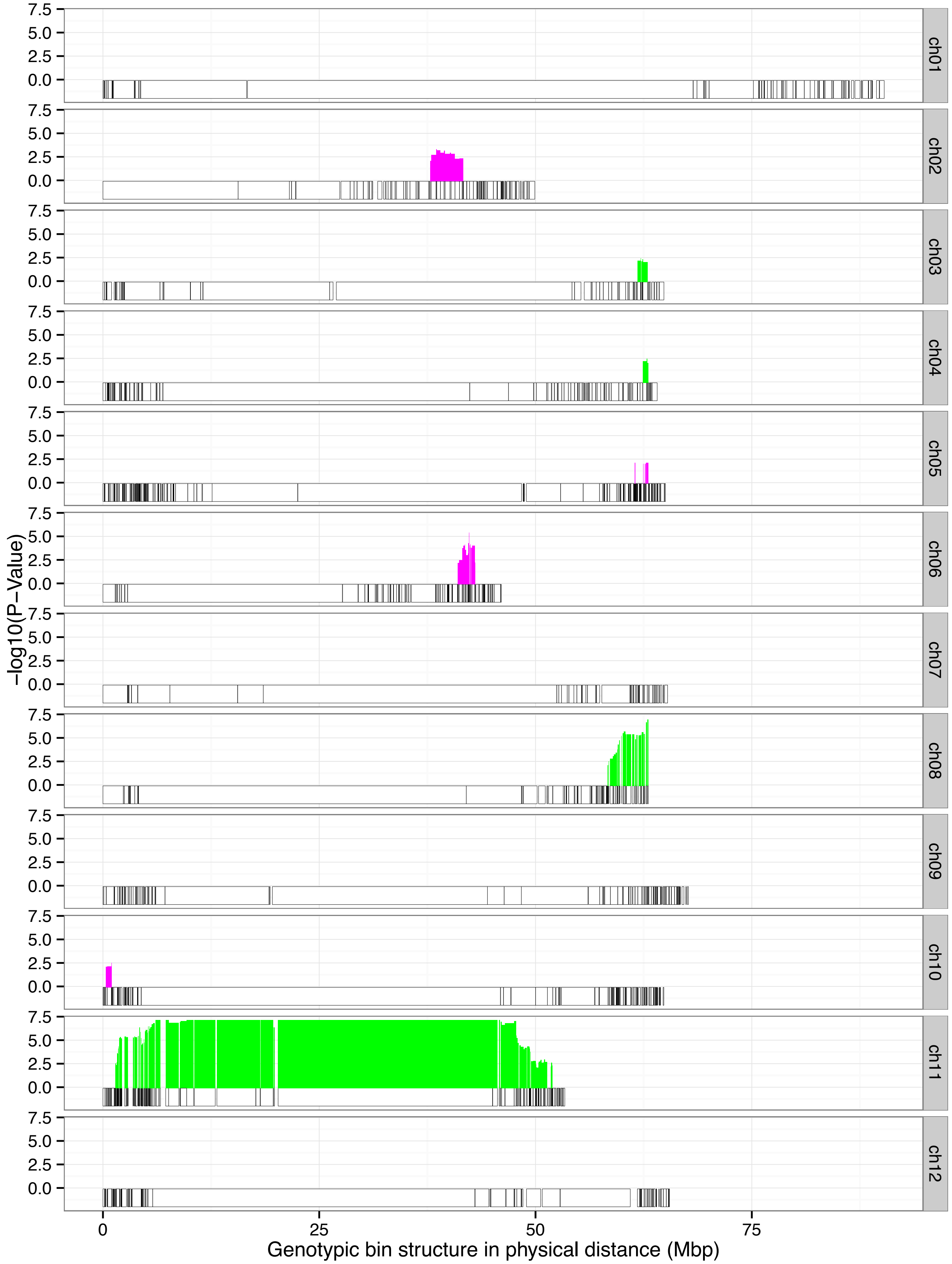

Figure S39. Leaflet symmetric EFD PC1 marginal regression QTL mapping results. QTL sign (phenotypic effect) is denoted by color: positive = green, negative = magenta.

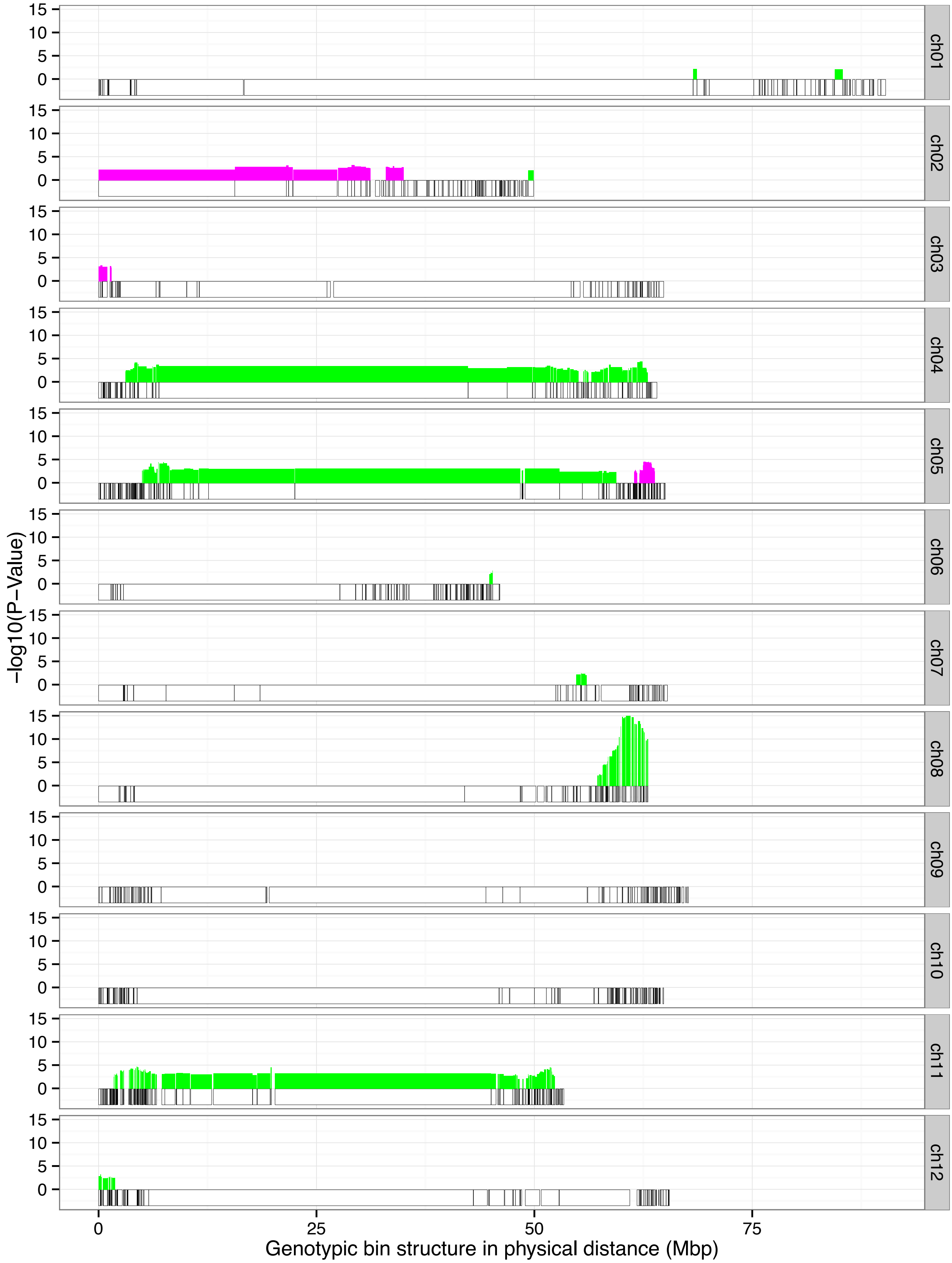

Figure S40. Leaflet symmetric EFD PC2 marginal regression QTL mapping results. QTL sign (phenotypic effect) is denoted by color: positive = green, negative = magenta.

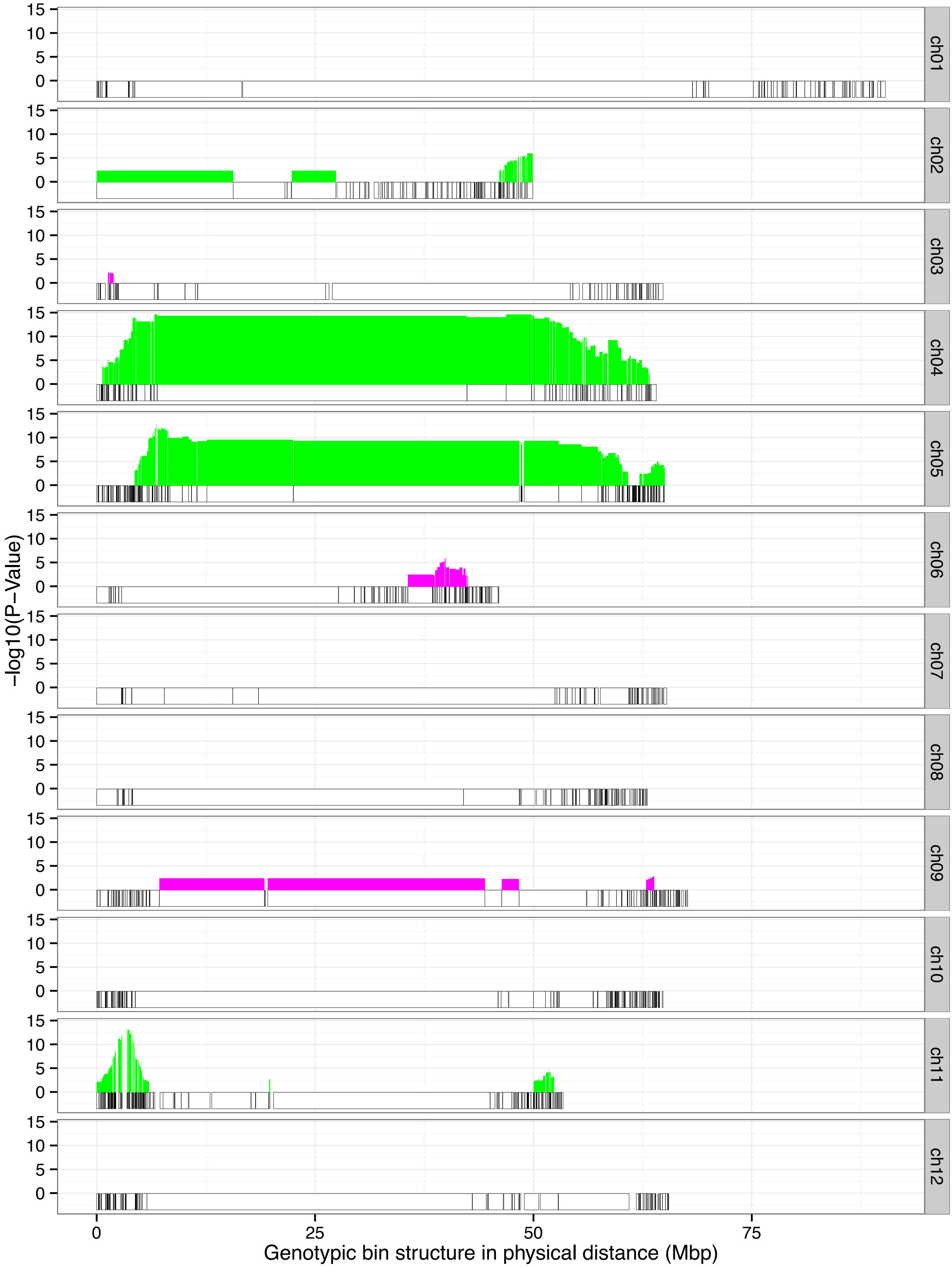

Figure S41. Leaflet symmetric EFD PC3 marginal regression QTL mapping results. QTL sign (phenotypic effect) is denoted by color: positive = green, negative = magenta.

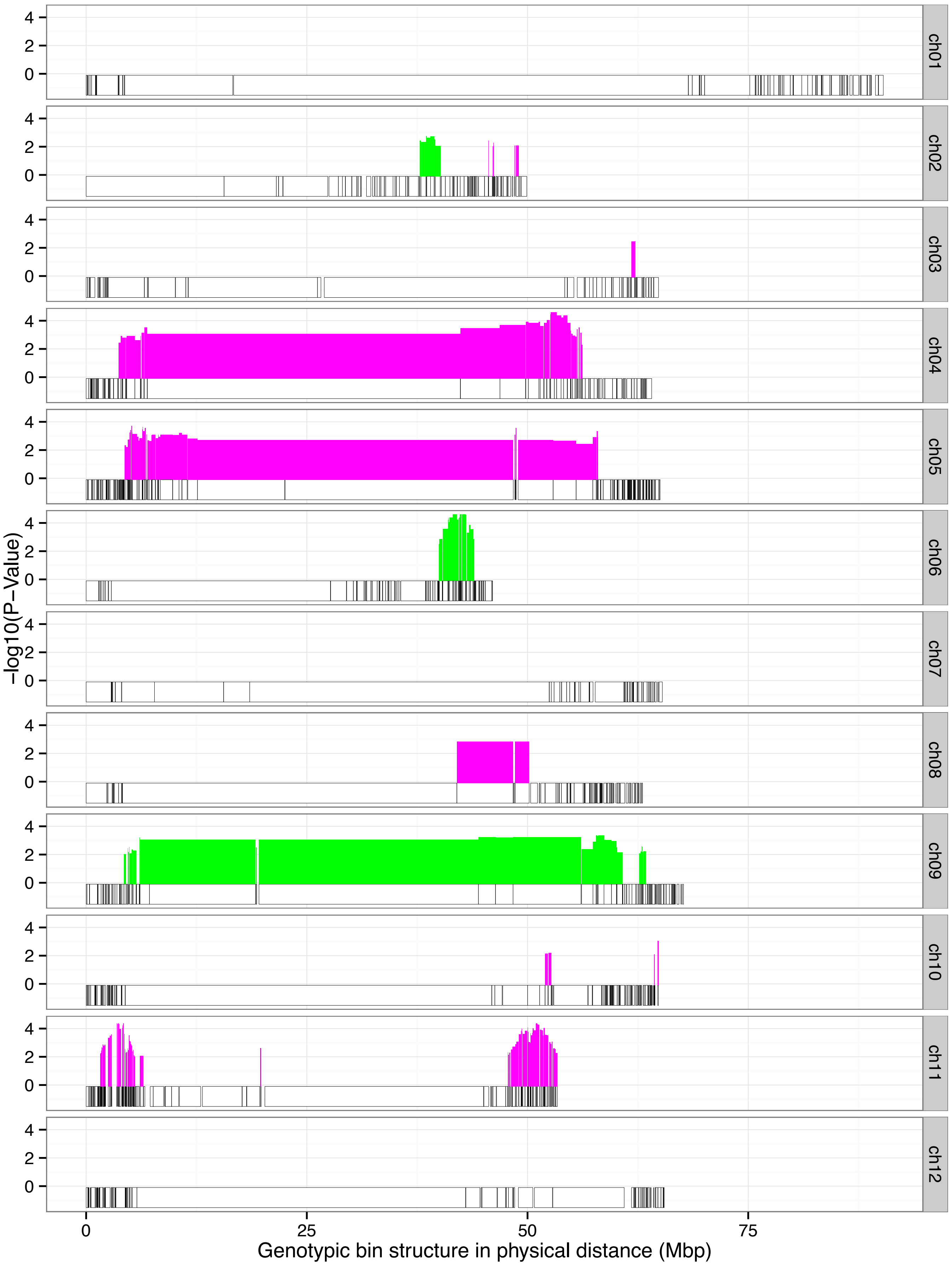

Figure S42. Leaflet symmetric EFD PC4 marginal regression QTL mapping results. QTL sign (phenotypic effect) is denoted by color: positive = green, negative = magenta.

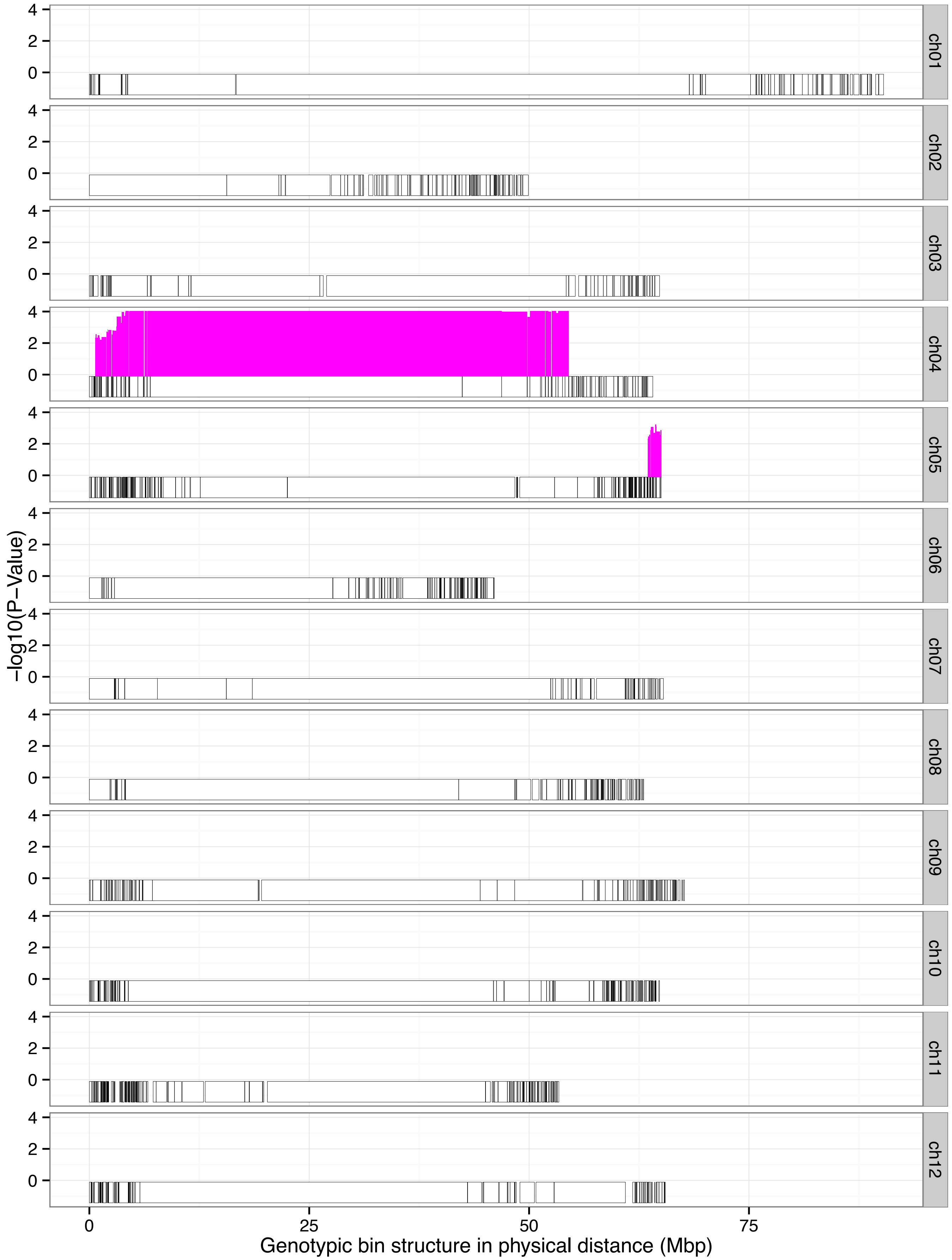

Figure S43. Leaflet asymmetric EFD PC1 marginal regression QTL mapping results. QTL sign (phenotypic effect) is denoted by color: positive = green, negative = magenta.

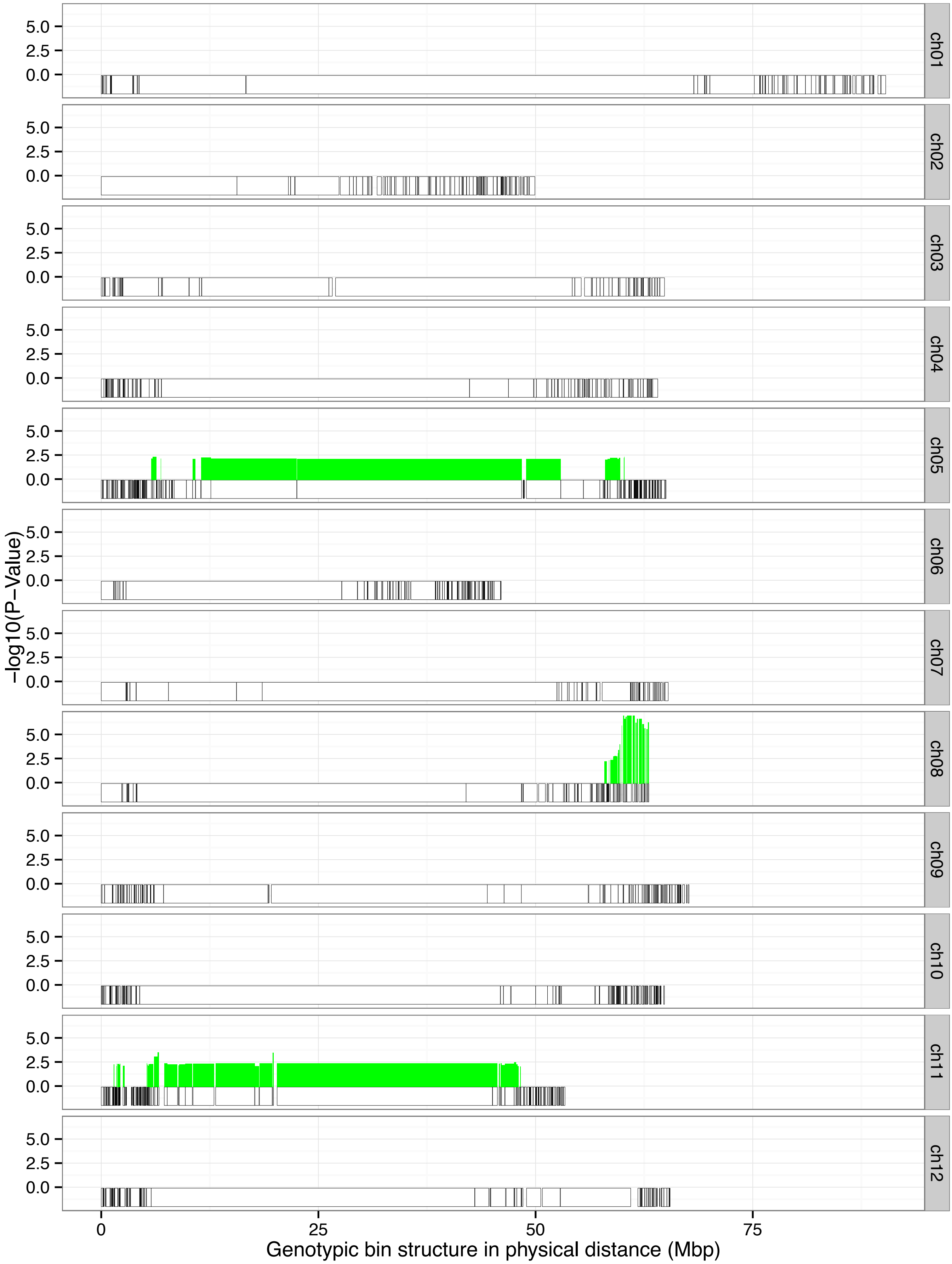

Figure S44. Leaflet asymmetric EFD PC2 marginal regression QTL mapping results. QTL sign (phenotypic effect) is denoted by color: positive = green, negative = magenta.

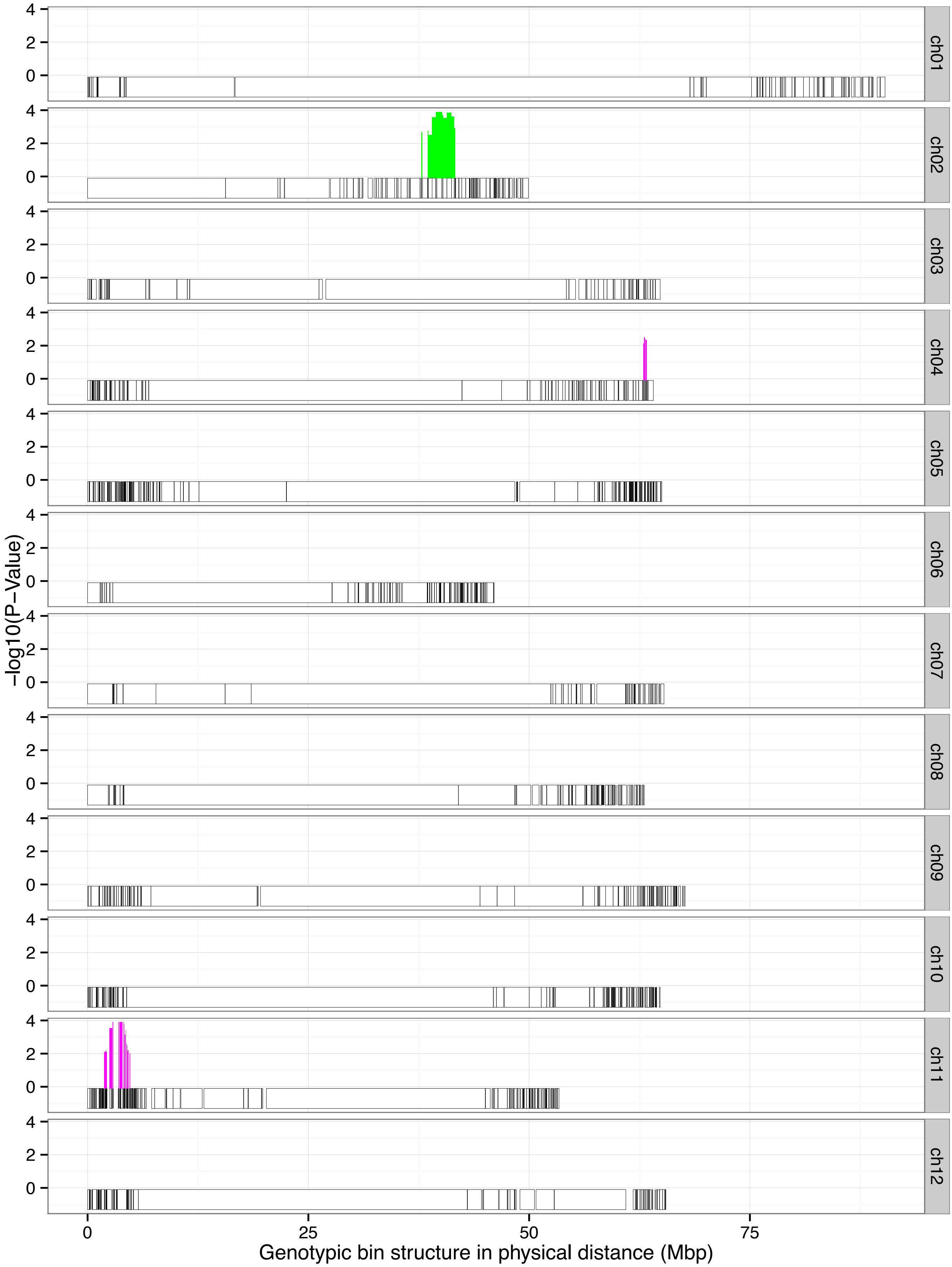

Figure S45. Leaflet asymmetric EFD PC4 marginal regression QTL mapping results. QTL sign (phenotypic effect) is denoted by color: positive = green, negative = magenta.

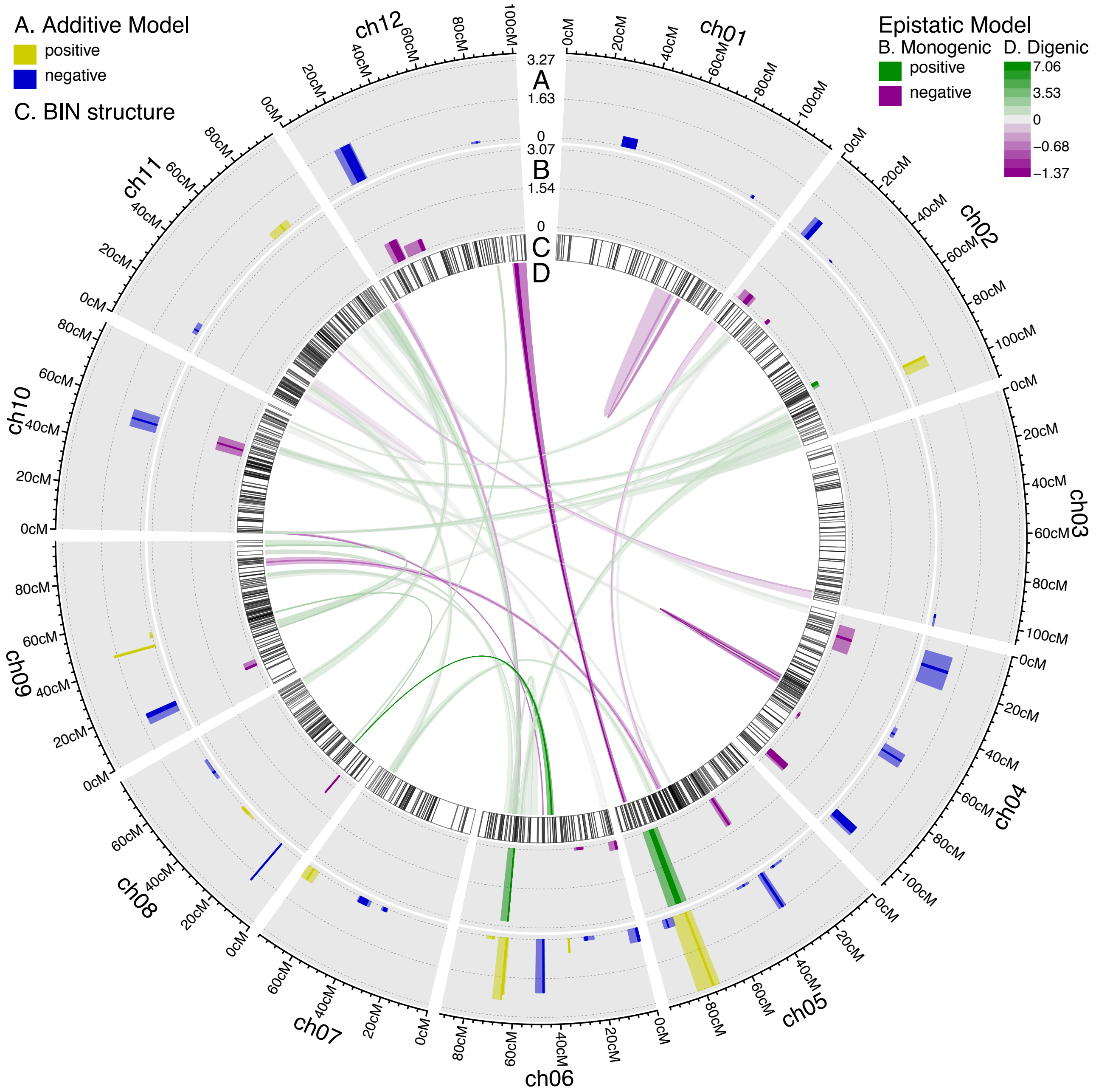

Figure S46. Intercalary leaf complexity SparseNet QTL mapping results. The bin selected by SparseNet as a QTL is colored opaquely, and the nearby bins correlated to it at or greater than 0.9 are taken as an approximate QTL interval and colored translucently.

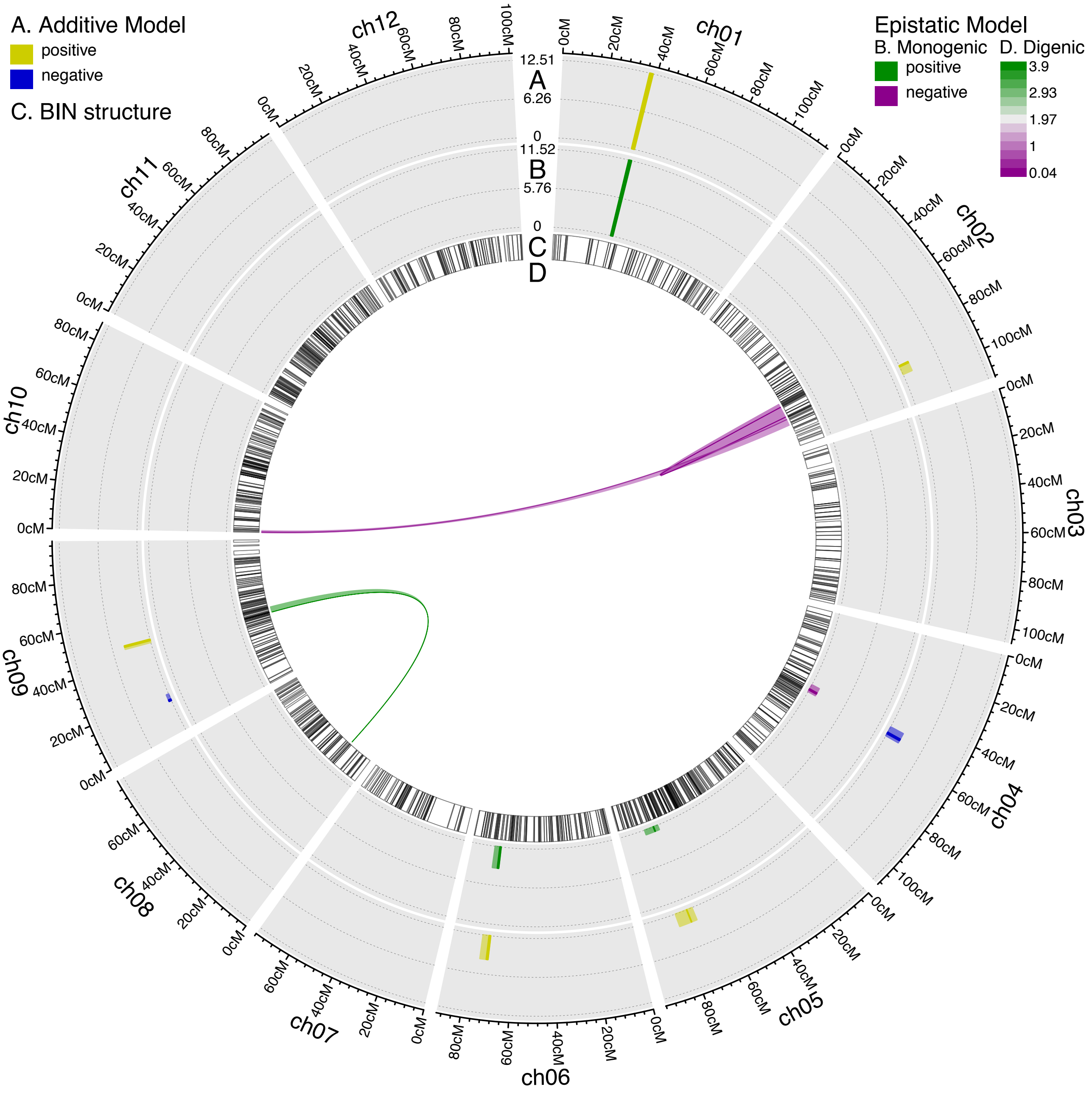

Figure S47. Secondary leaf complexity SparseNet QTL mapping results. The bin selected by SparseNet as a QTL is colored opaquely, and the nearby bins correlated at or greater than 0.9 are taken as an approximate QTL interval and colored translucently.

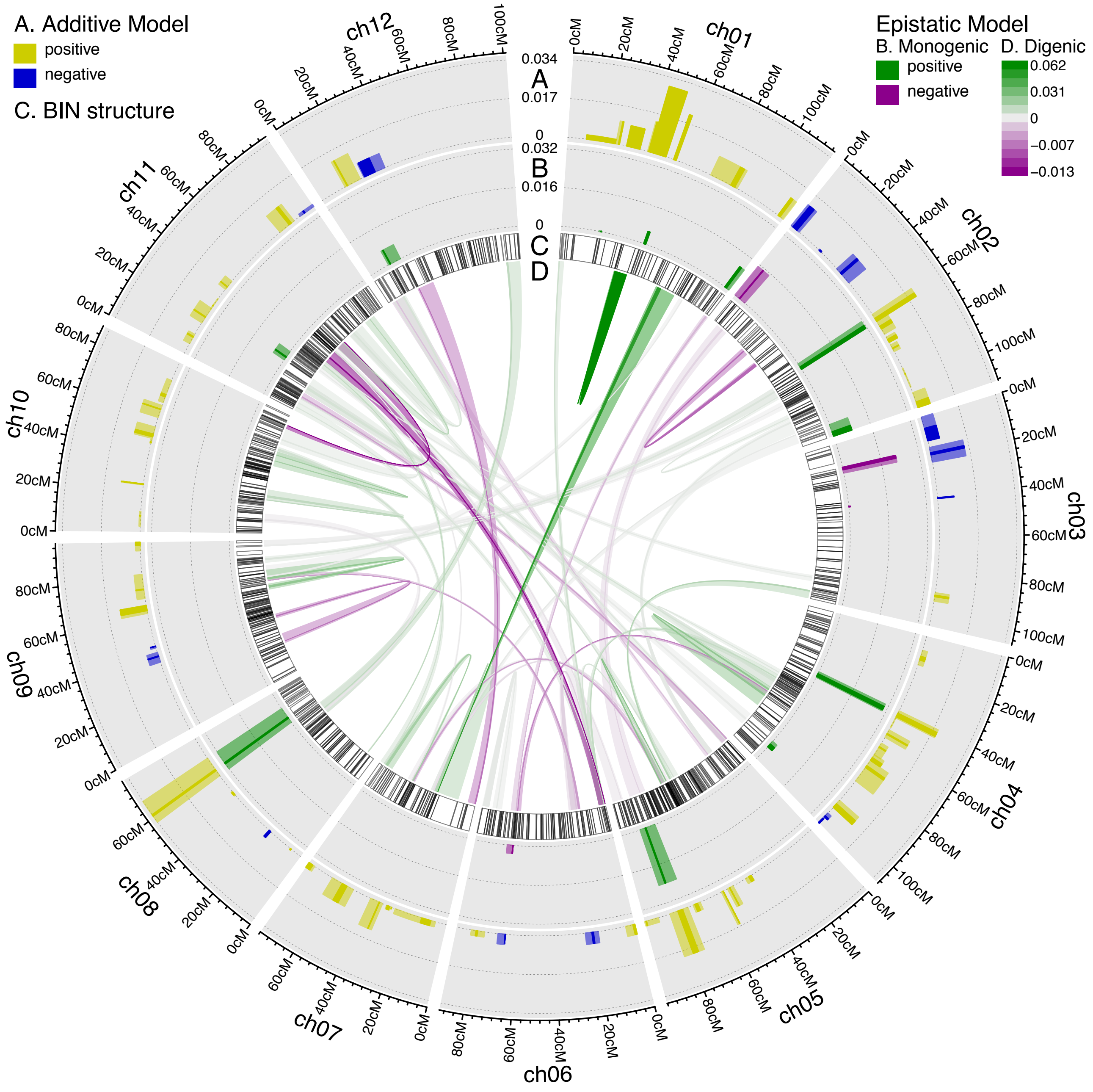

Figure S48. Leaflet roundness SparseNet QTL mapping results. The bin selected by SparseNet as a QTL is colored opaquely, and the nearby bins correlated to it at or greater than 0.9 are taken as an approximate QTL interval and colored translucently.

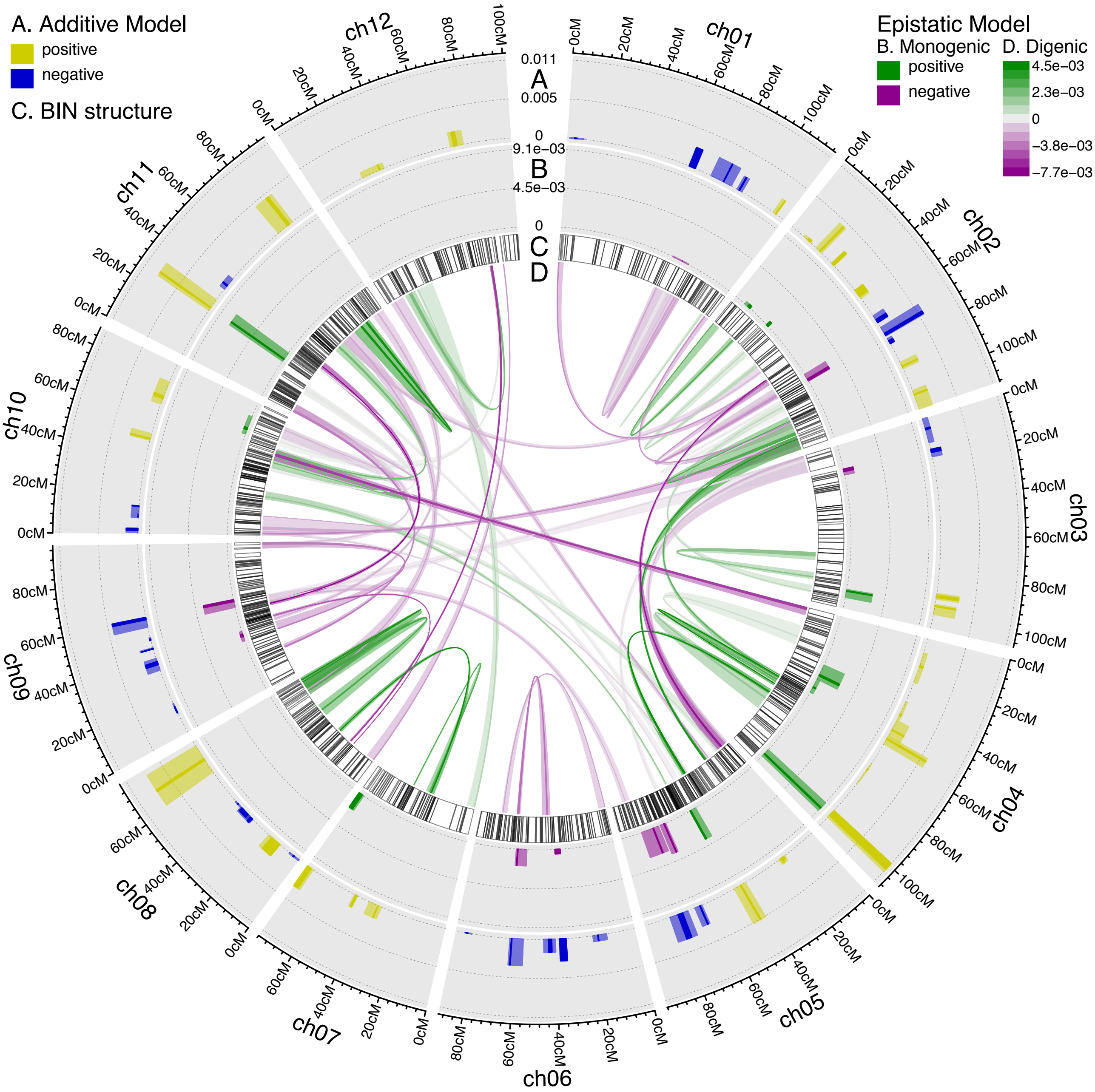

Figure S49. Leaflet solidity SparseNet QTL mapping results. The bin selected by SparseNet as a QTL is colored opaquely, and the nearby bins correlated to it at or greater than 0.9 are taken as an approximate QTL interval and colored translucently.

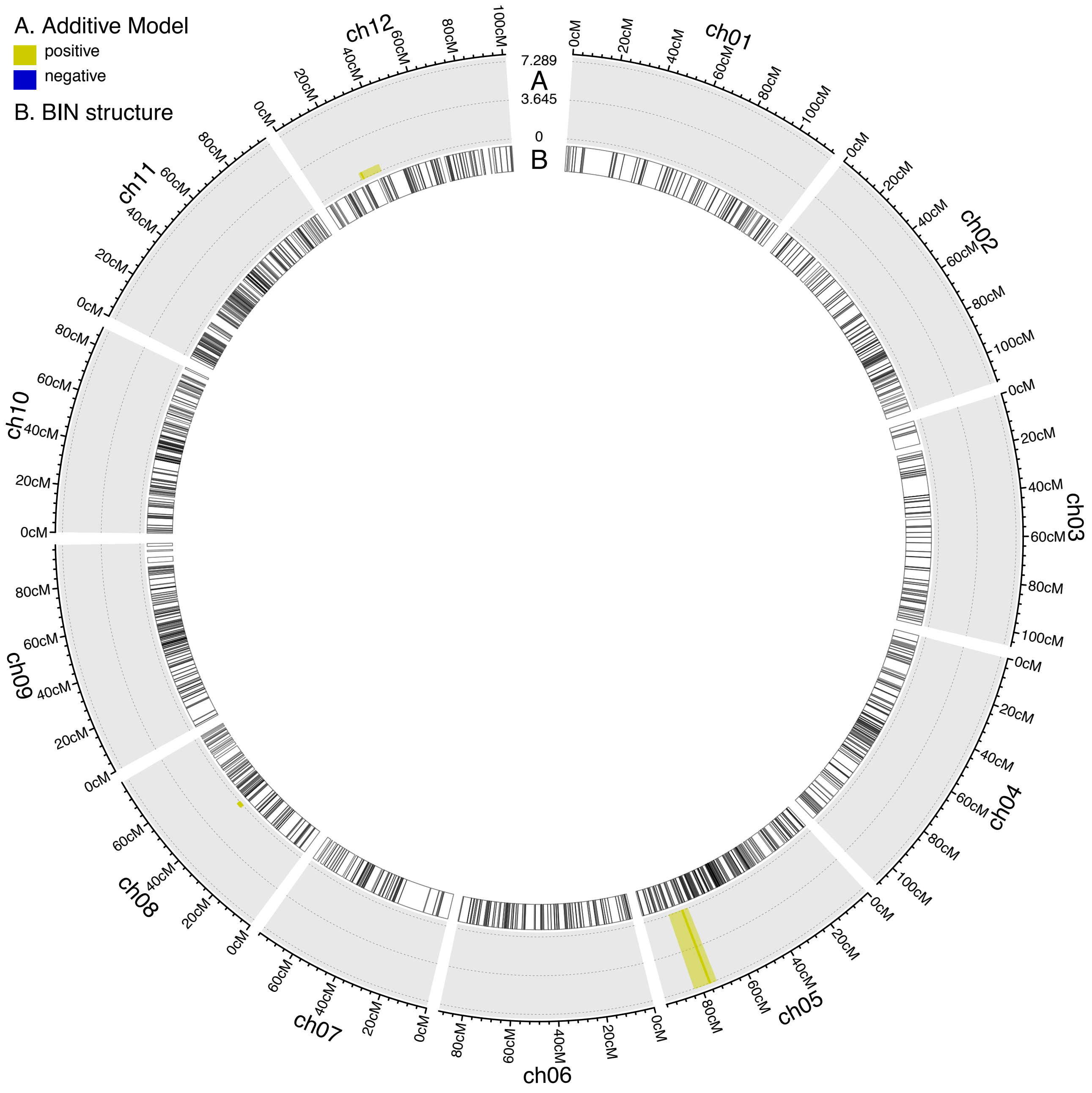

Figure S50. Flowering time (days to first anthesis) SparseNet QTL mapping results. The bin selected by SparseNet as a QTL is colored opaquely, and the nearby bins correlated to it at or greater than 0.9 are taken as an approximate QTL interval and colored translucently.

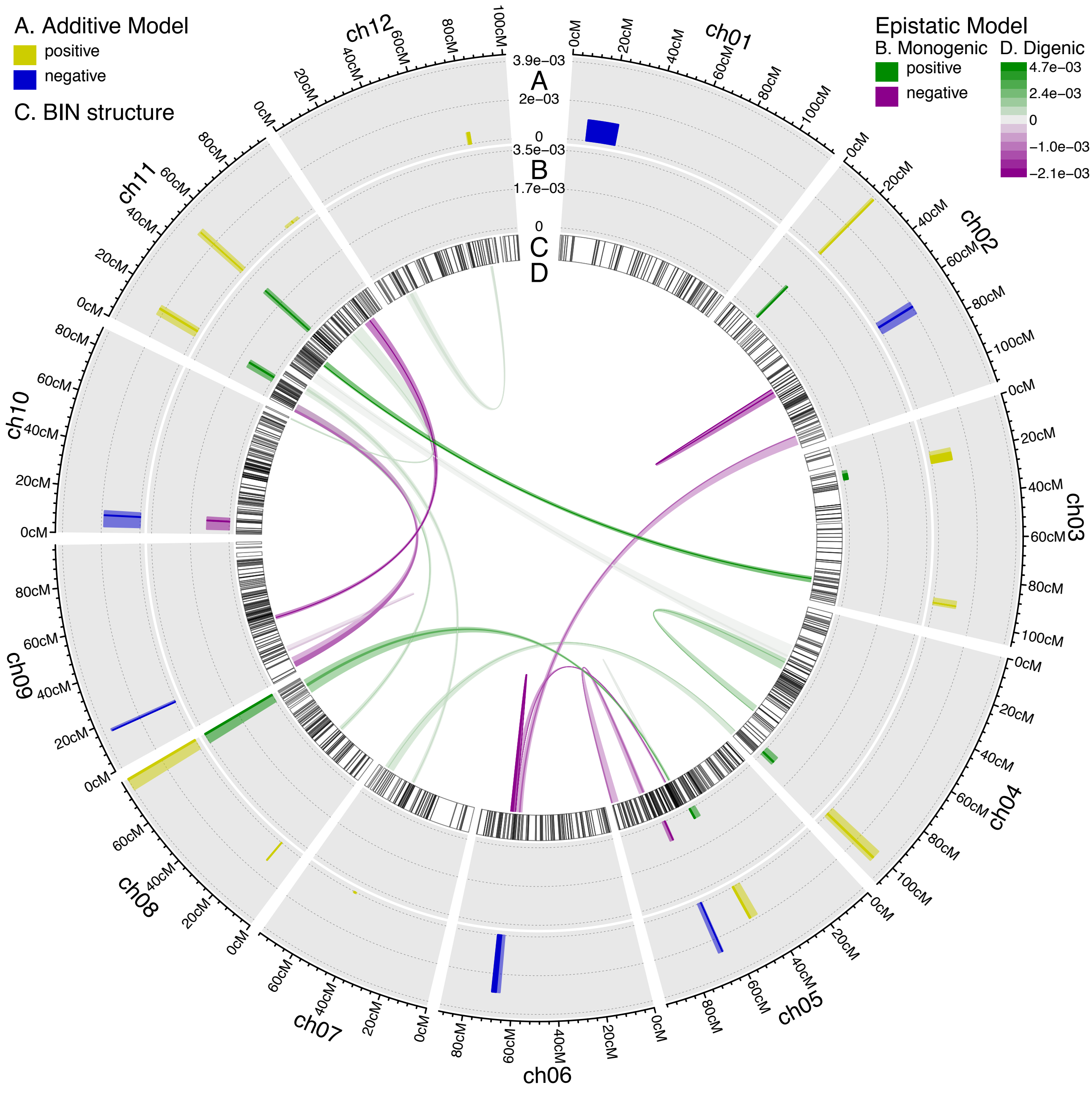

Figure S51. Leaflet symmetric EFD PC1 SparseNet QTL mapping results. The bin selected by SparseNet as a QTL is colored opaquely, and the nearby bins correlated to it at or greater than 0.9 are taken as an approximate QTL interval and colored translucently.

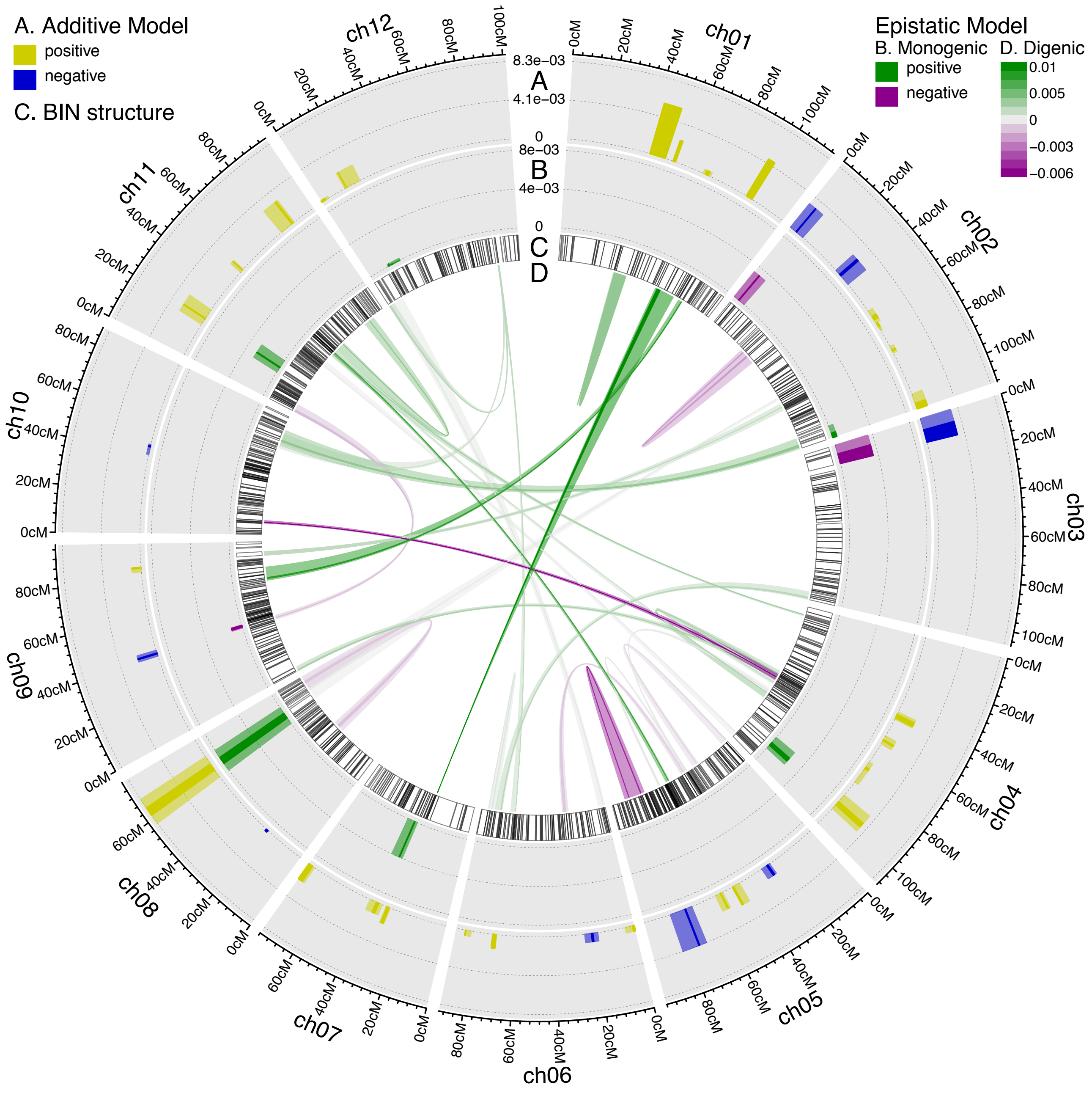

Figure S52. Leaflet symmetric EFD PC2 SparseNet QTL mapping results. The bin selected by SparseNet as a QTL is colored opaquely, and the nearby bins correlated to it at or greater than 0.9 are taken as an approximate QTL interval and colored translucently.

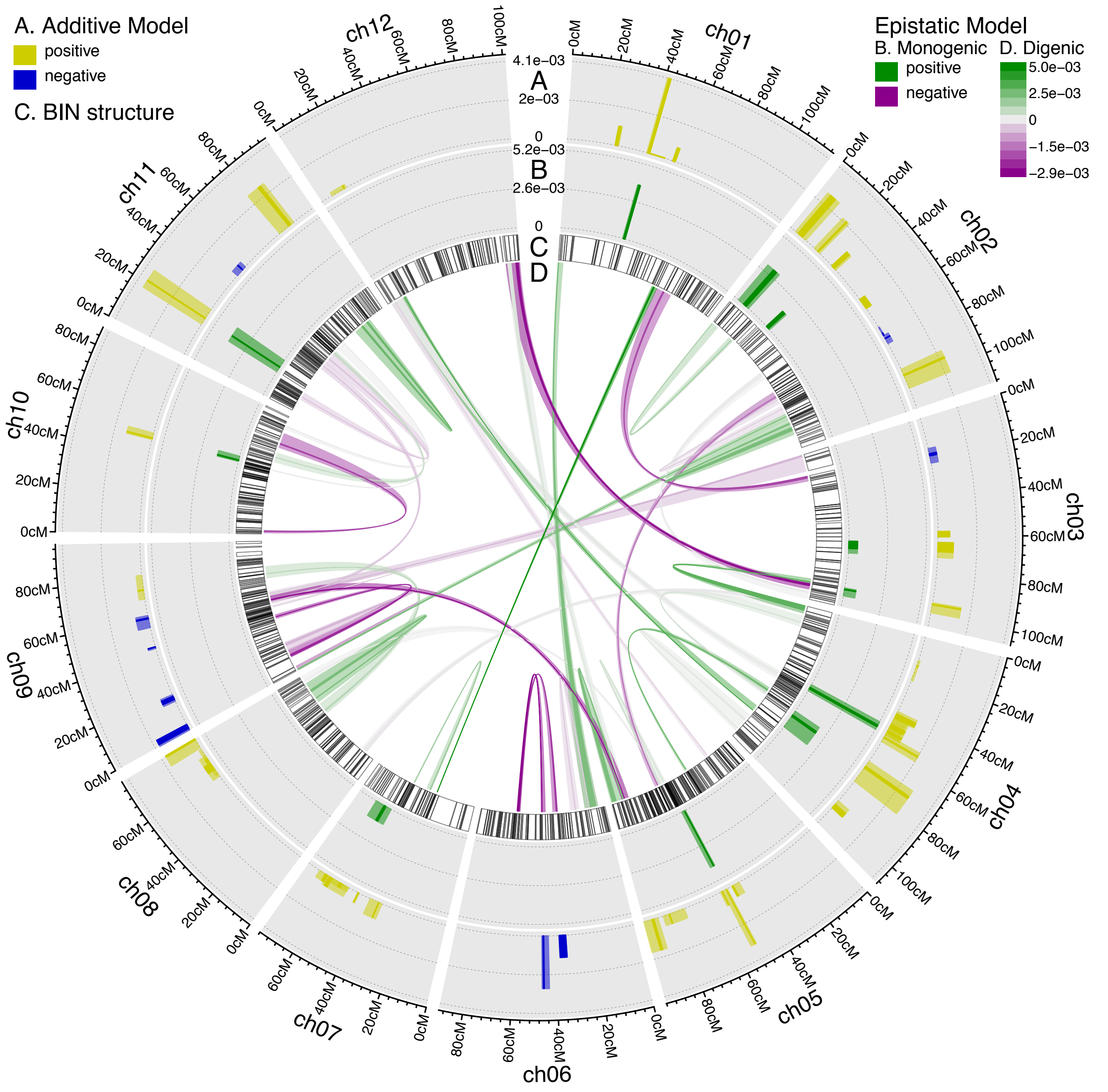

Figure S53. Leaflet symmetric EFD PC3 SparseNet QTL mapping results. The bin selected by SparseNet as a QTL is colored opaquely, and the nearby bins correlated to it at or greater than 0.9 are taken as an approximate QTL interval and colored translucently.

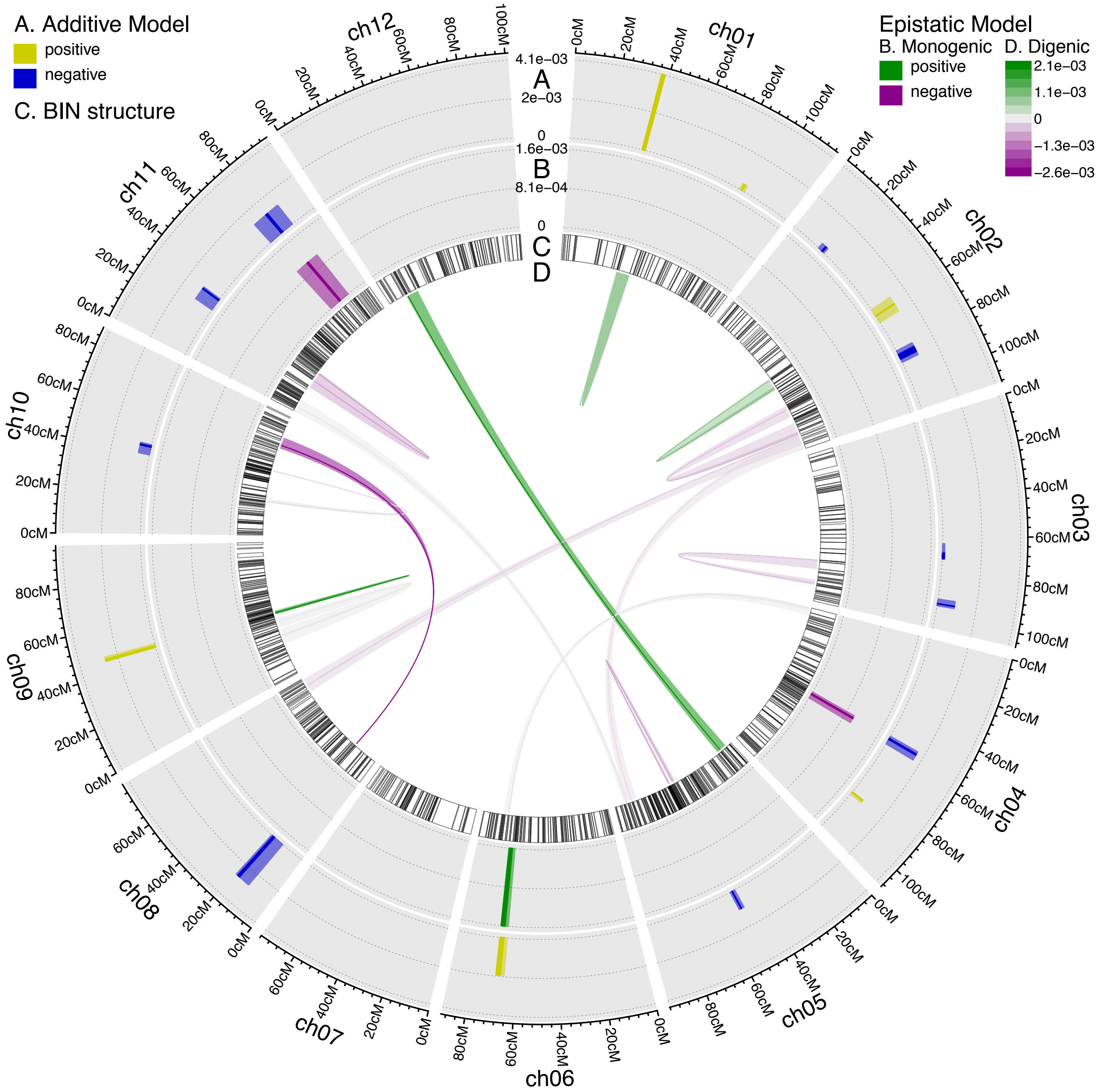

Figure S54. Leaflet symmetric EFD PC4 SparseNet QTL mapping results. The bin selected by SparseNet as a QTL is colored opaquely, and the nearby bins correlated to it at or greater than 0.9 are taken as an approximate QTL interval and colored translucently.

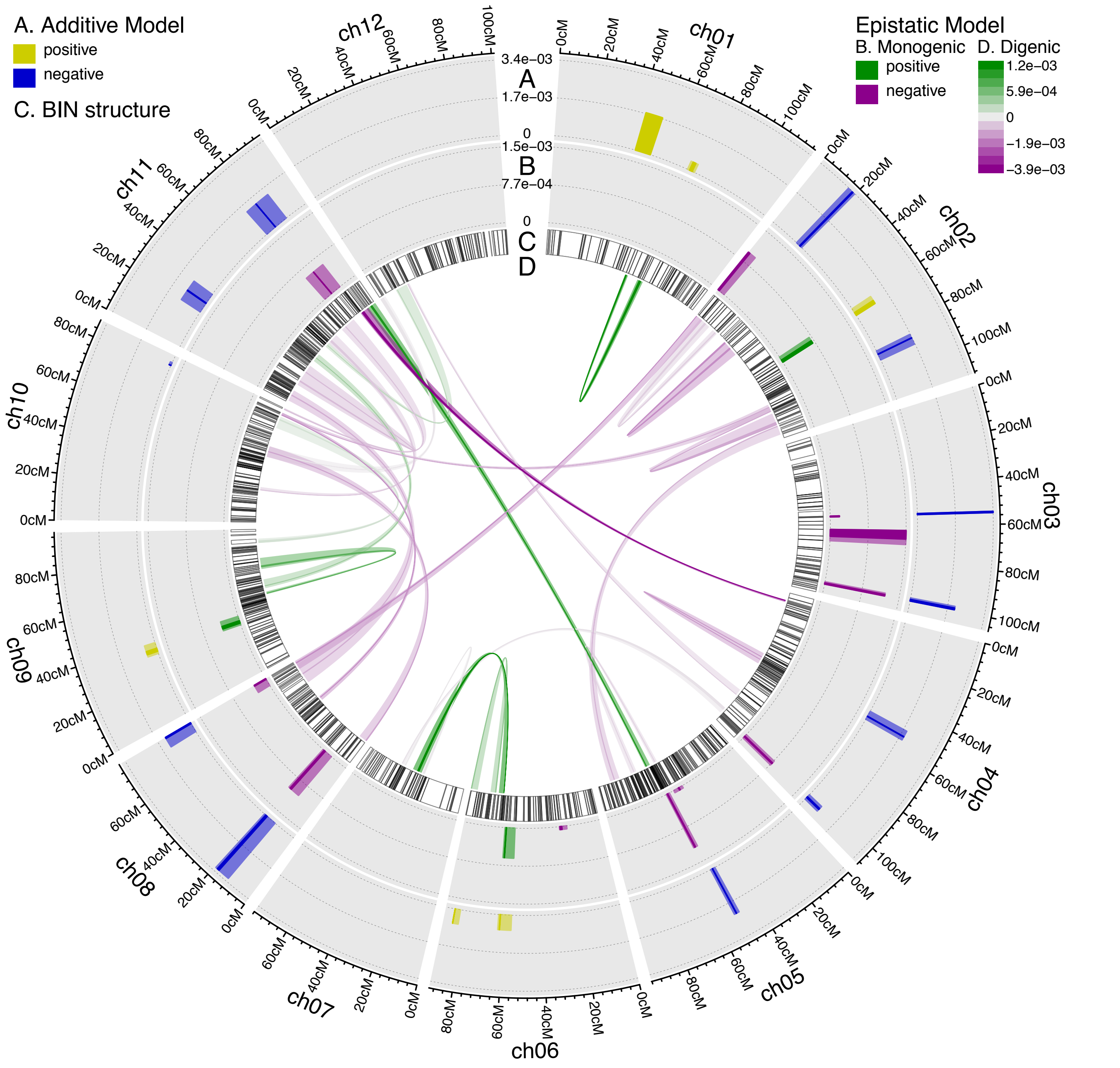

Figure S55. Leaflet symmetric EFD PC5 SparseNet QTL mapping results. The bin selected by SparseNet as a QTL is colored opaquely, and the nearby bins correlated to it at or greater than 0.9 are taken as an approximate QTL interval and colored translucently.

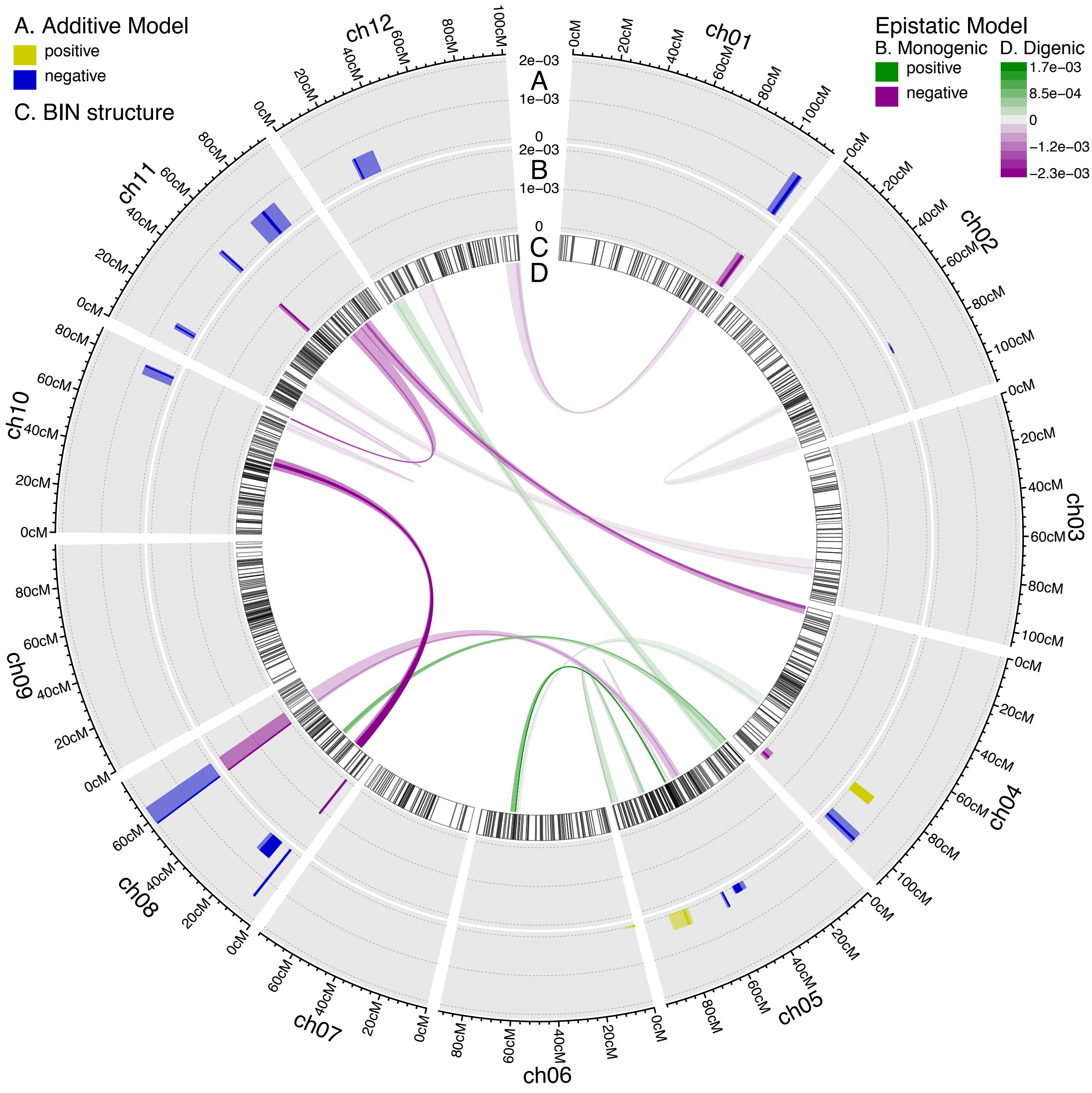

Figure S56. Leaflet symmetric EFD PC6 SparseNet QTL mapping results. The bin selected by SparseNet as a QTL is colored opaquely, and the nearby bins correlated to it at or greater than 0.9 are taken as an approximate QTL interval and colored translucently.

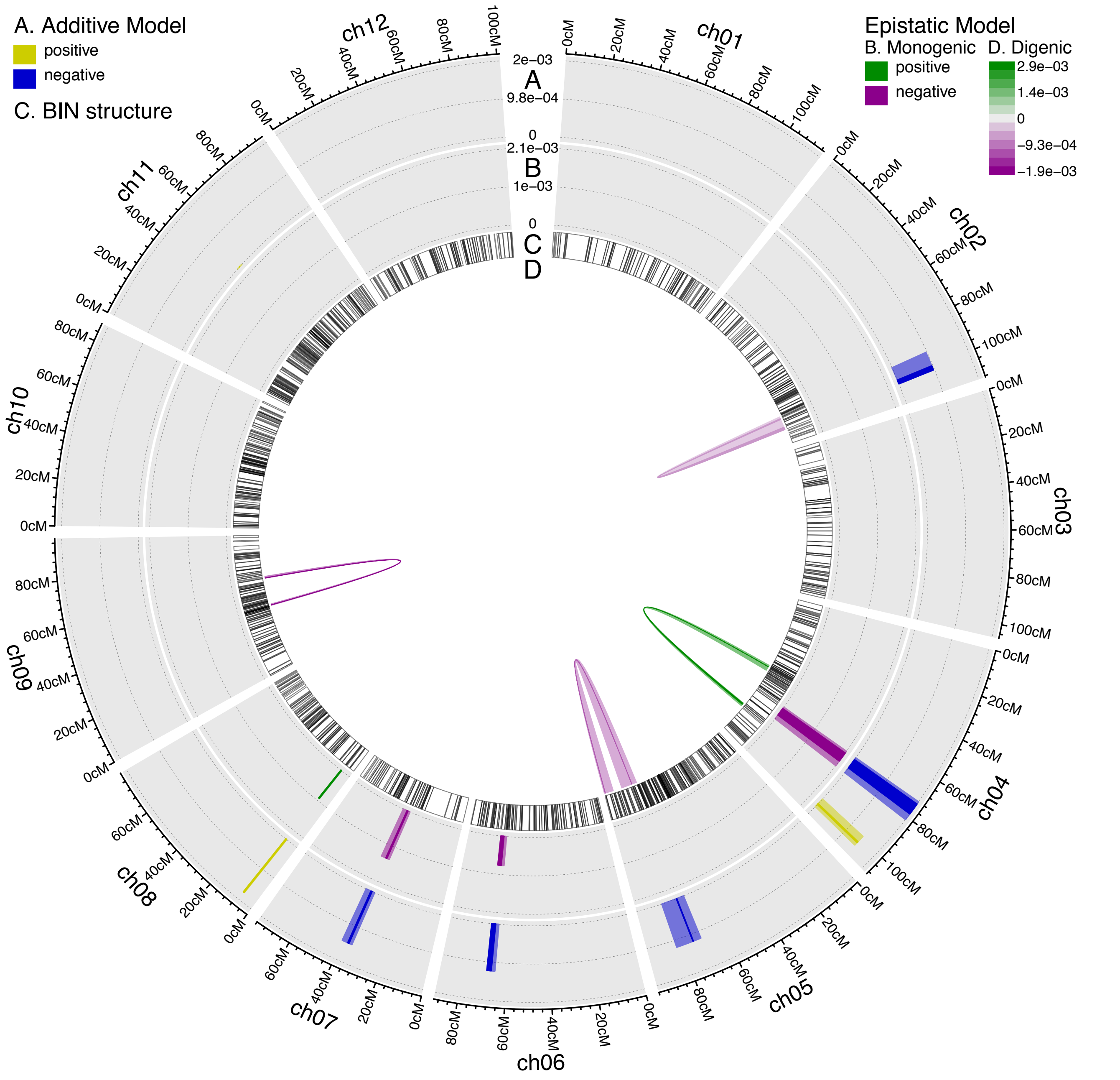

Figure S57. Leaflet symmetric EFD PC7 SparseNet QTL mapping results. The bin selected by SparseNet as a QTL is colored opaquely, and the nearby bins correlated to it at or greater than 0.9 are taken as an approximate QTL interval and colored translucently.

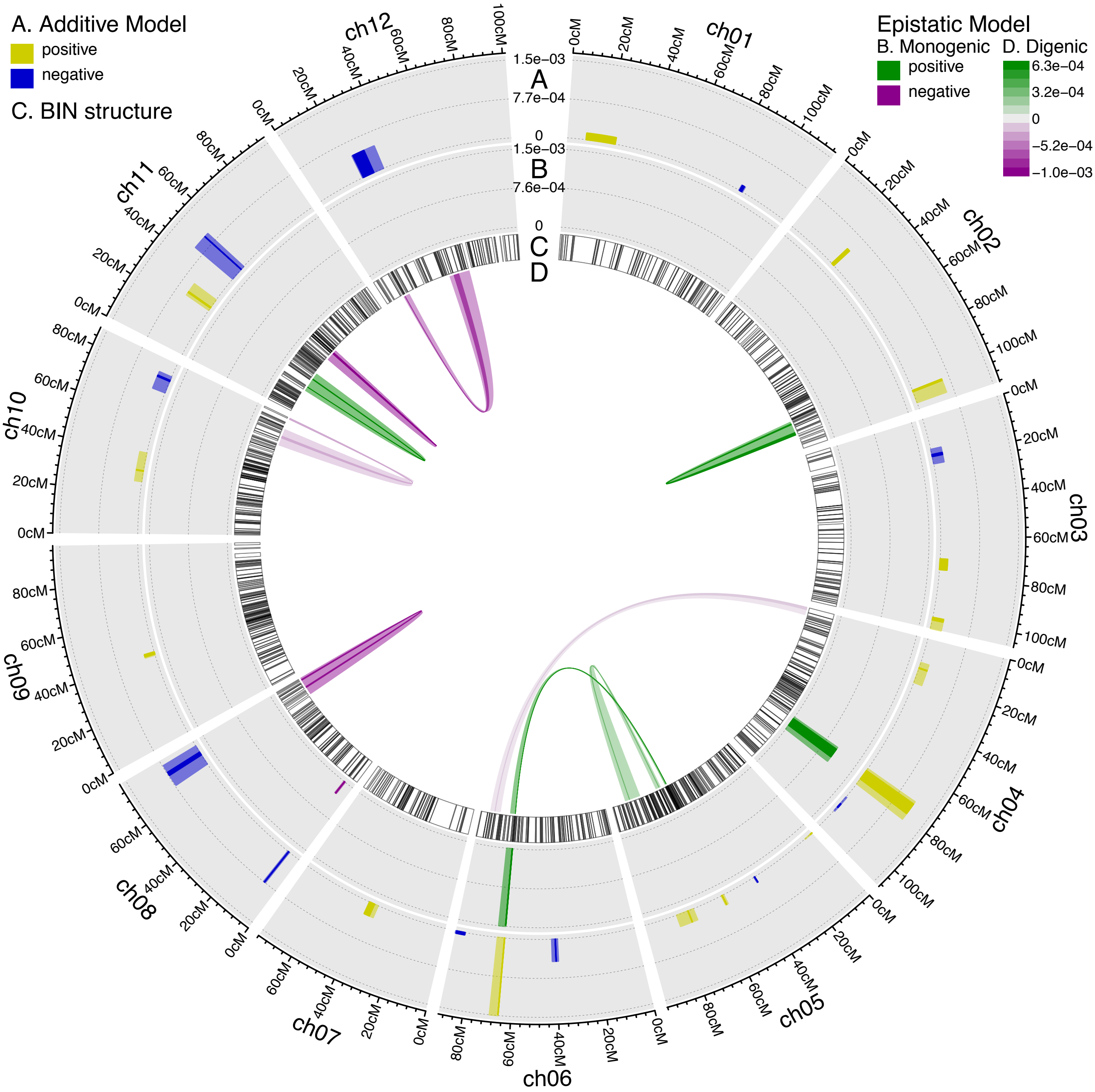

Figure S58. Leaflet symmetric EFD PC8 SparseNet QTL mapping results. The bin selected by SparseNet as a QTL is colored opaquely, and the nearby bins correlated to it at or greater than 0.9 are taken as an approximate QTL interval and colored translucently.

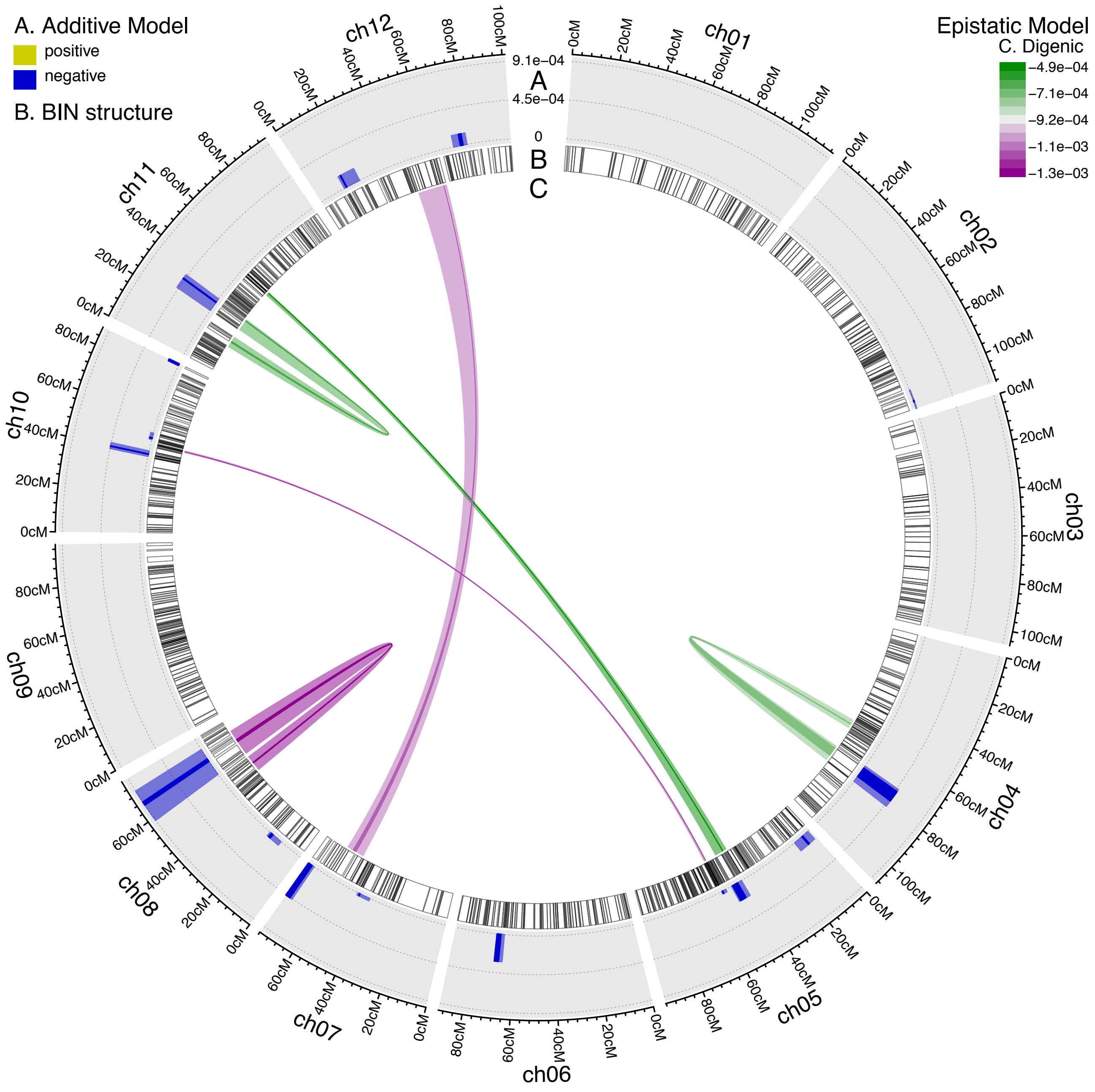

Figure S59. Leaflet symmetric EFD PC9 SparseNet QTL mapping results. The bin selected by SparseNet as a QTL is colored opaquely, and the nearby bins correlated to it at or greater than 0.9 are taken as an approximate QTL interval and colored translucently.

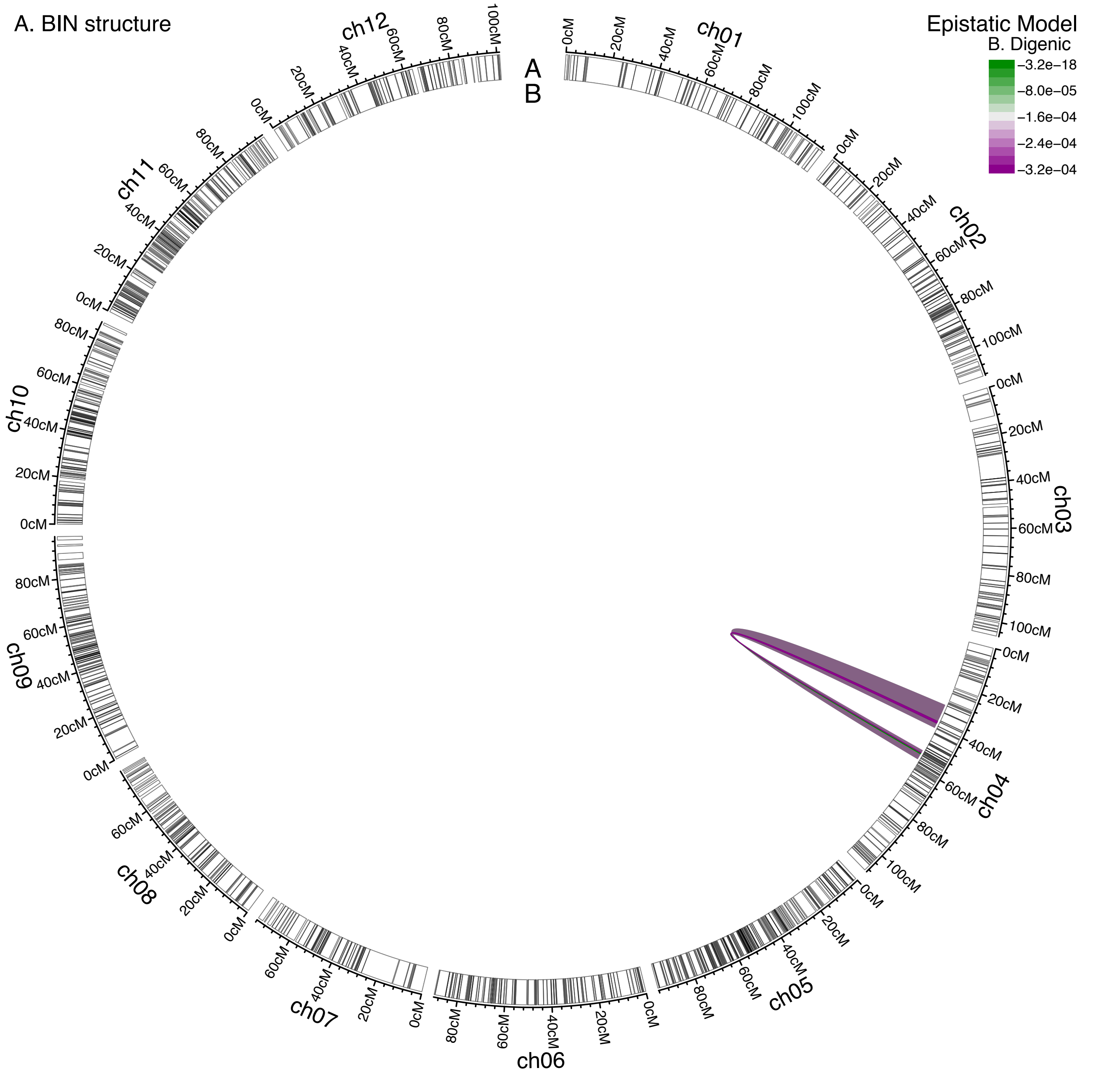

Figure S60. Leaflet asymmetric EFD PC1 SparseNet QTL mapping results. The bin selected by SparseNet as a QTL is colored opaquely, and the nearby bins correlated to it at or greater than 0.9 are taken as an approximate QTL interval and colored translucently.

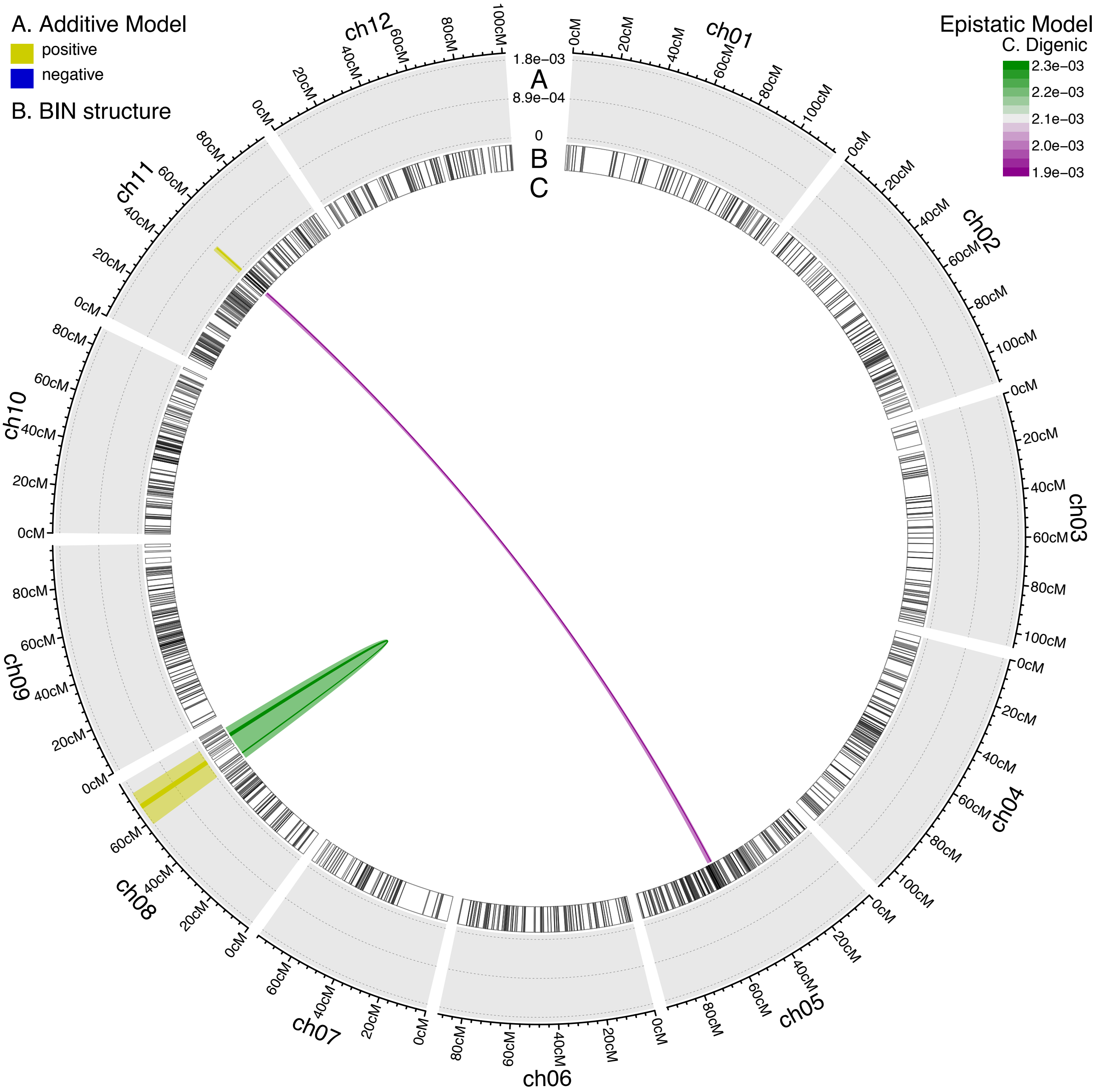

Figure S61. Leaflet asymmetric EFD PC2 SparseNet QTL mapping results. The bin selected by SparseNet as a QTL is colored opaquely, and the nearby bins correlated to it at or greater than 0.9 are taken as an approximate QTL interval and colored translucently.

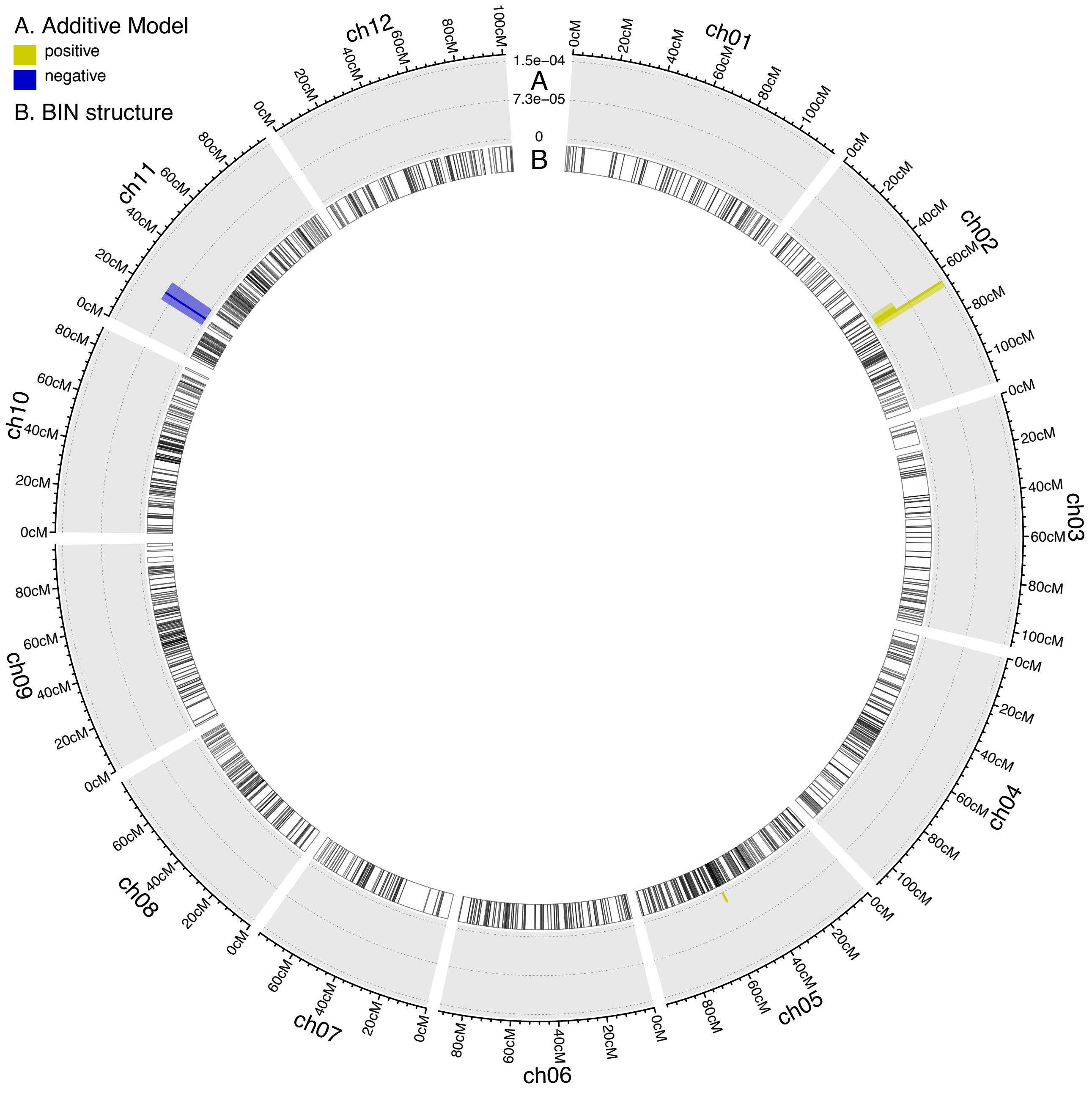

Figure S62. Leaflet asymmetric EFD PC3 SparseNet QTL mapping results. The bin selected by SparseNet as a QTL is colored opaquely, and the nearby bins correlated to it at or greater than 0.9 are taken as an approximate QTL interval and colored translucently.

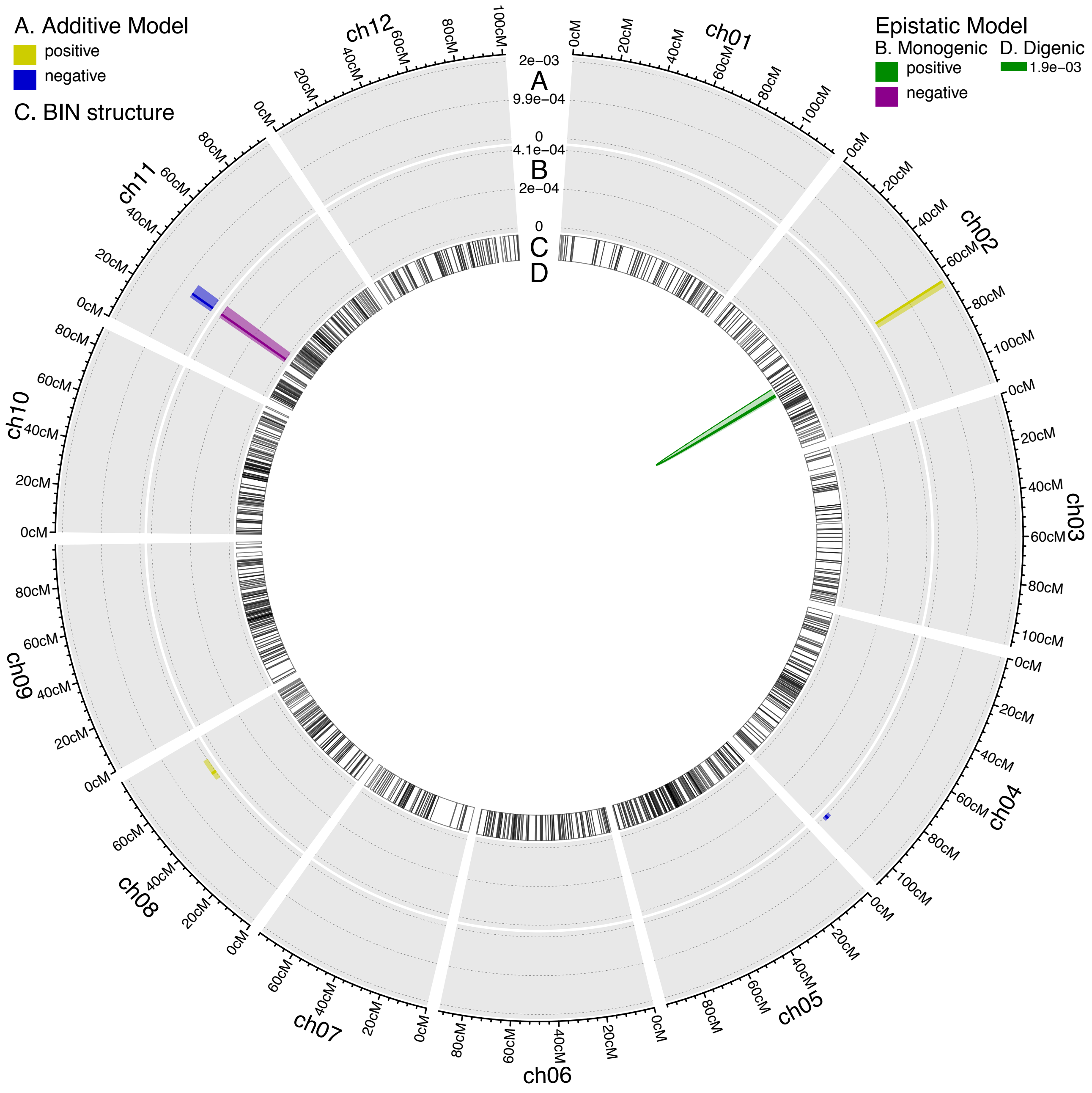

Figure S63. Leaflet asymmetric EFD PC4 SparseNet QTL mapping results. The bin selected by SparseNet as a QTL is colored opaquely, and the nearby bins correlated to it at or greater than 0.9 are taken as an approximate QTL interval and colored translucently.

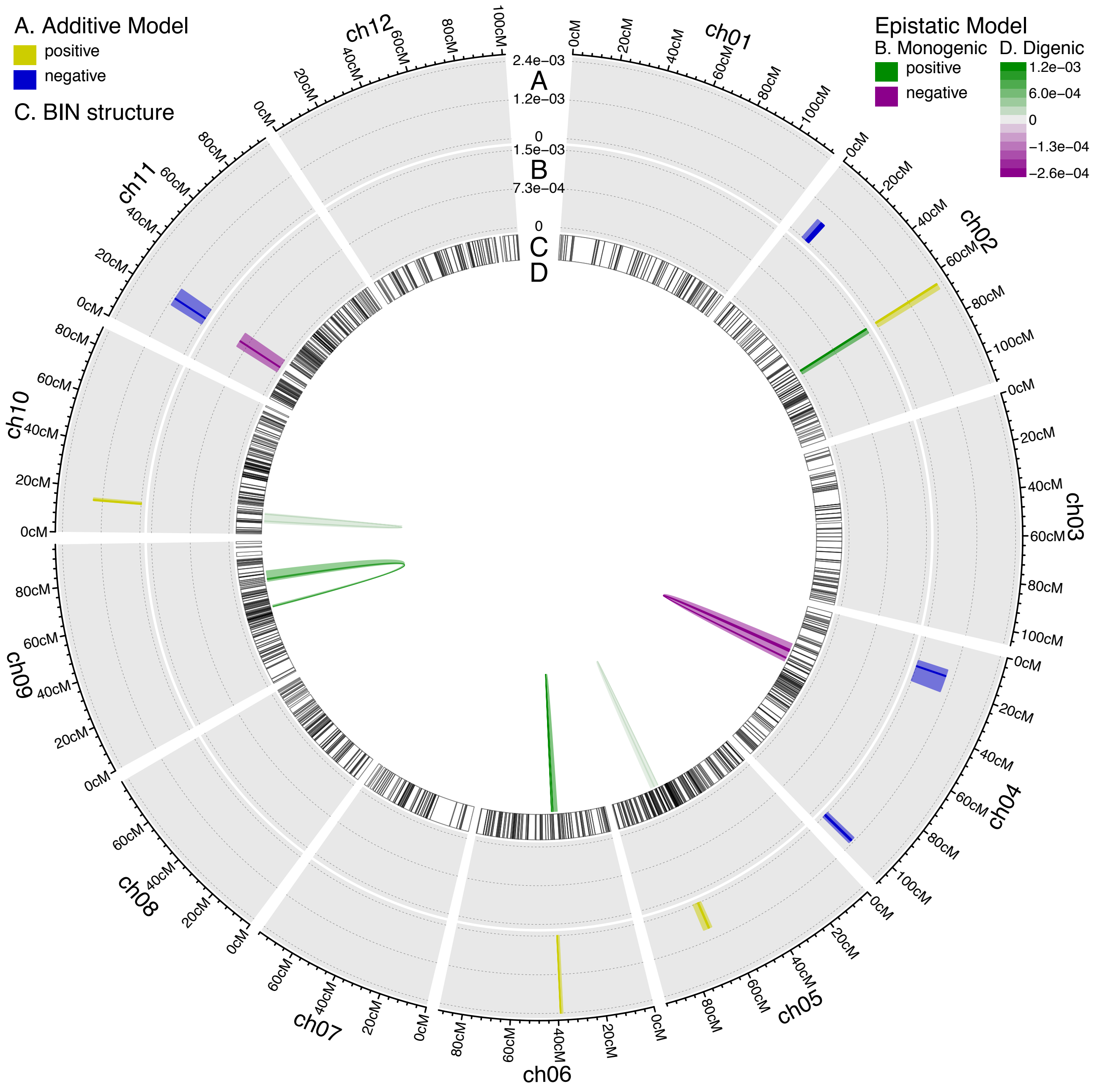

Figure S64. Leaflet asymmetric EFD PC5 SparseNet QTL mapping results. The bin selected by SparseNet as a QTL is colored opaquely, and the nearby bins correlated to it at or greater than 0.9 are taken as an approximate QTL interval and colored translucently.

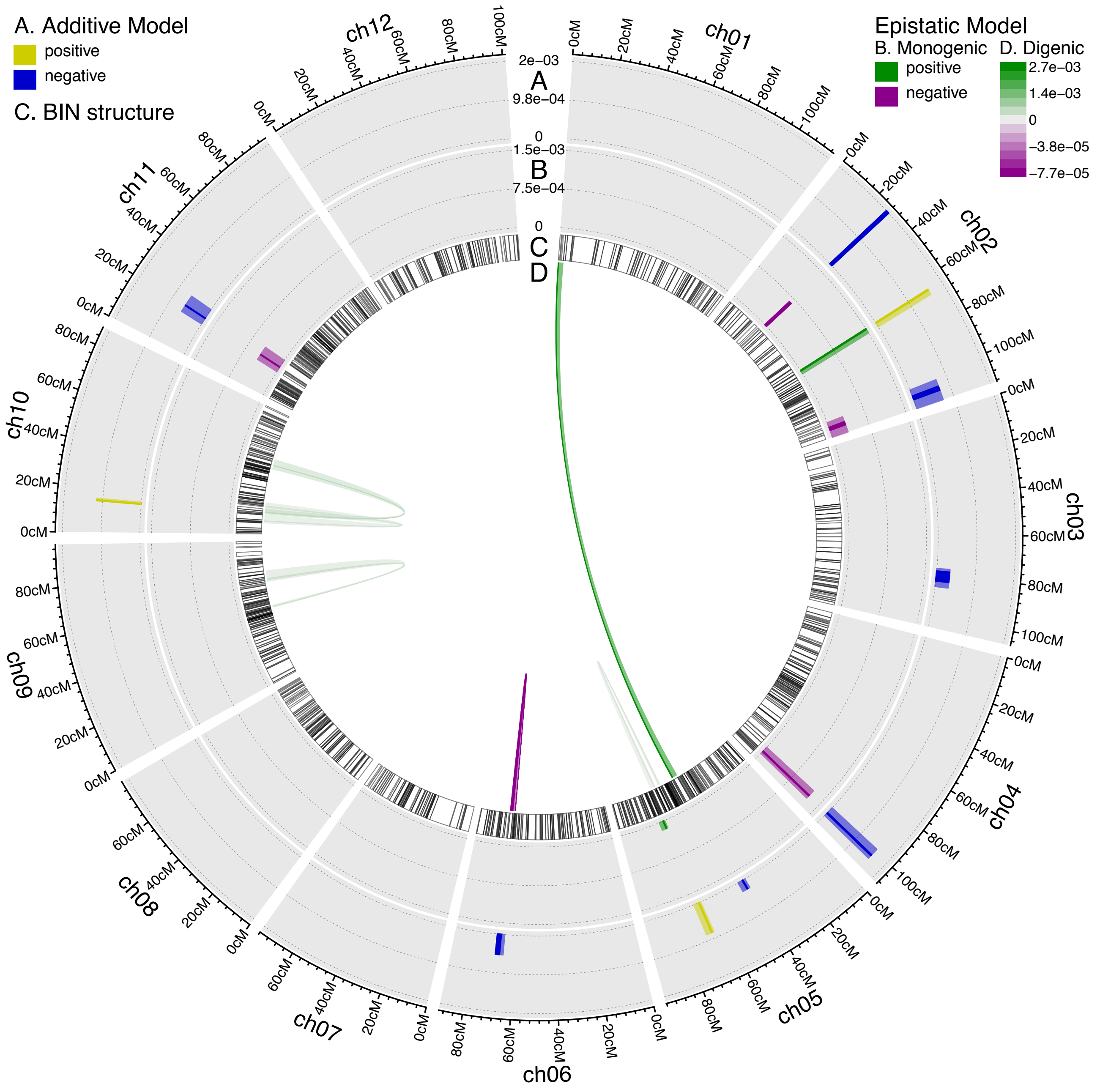

Figure S65. Leaflet asymmetric EFD PC6 SparseNet QTL mapping results. The bin selected by SparseNet as a QTL is colored opaquely, and the nearby bins correlated to it at or greater than 0.9 are taken as an approximate QTL interval and colored translucently.

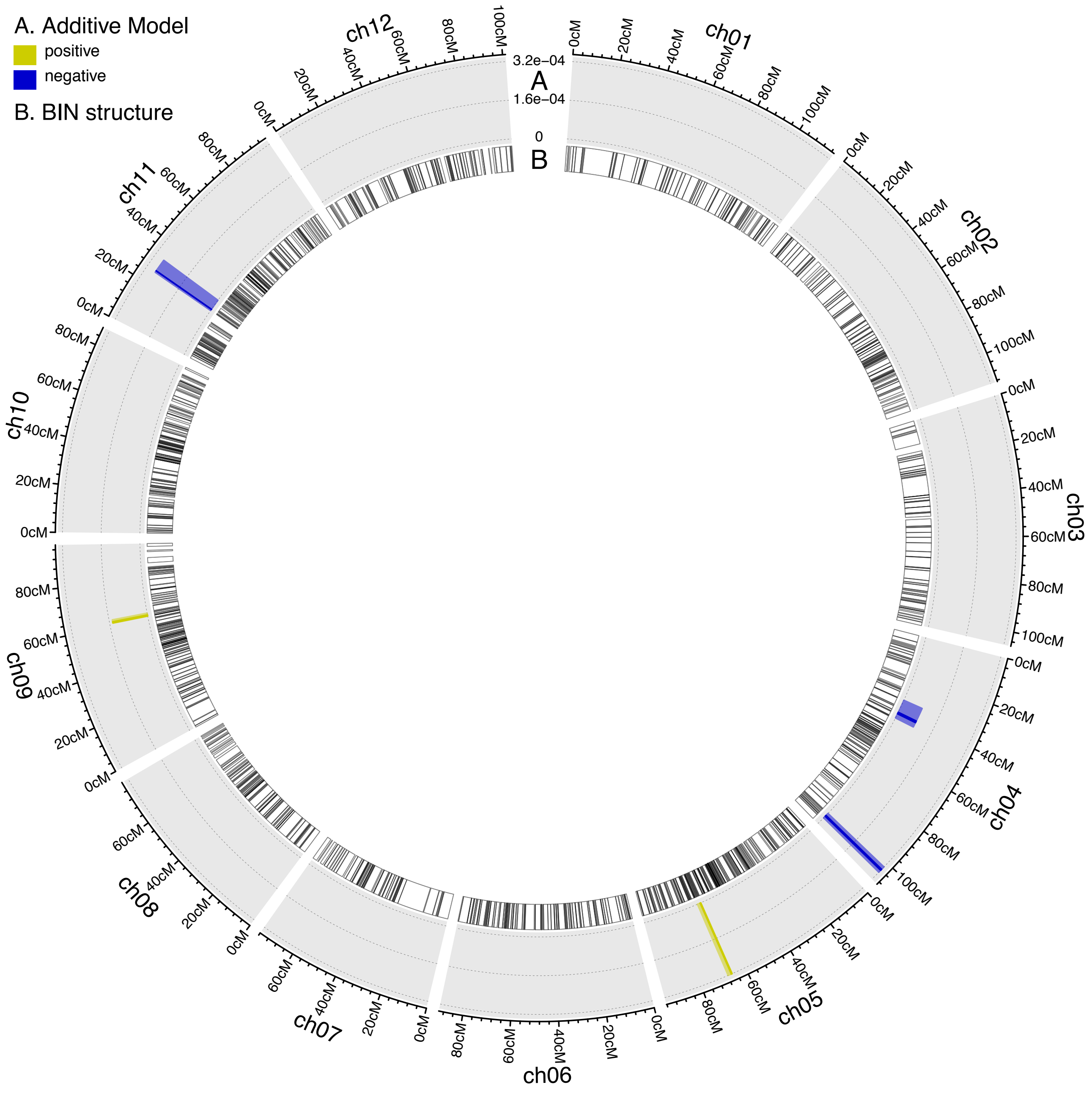

Figure S66. Leaflet asymmetric EFD PC7 SparseNet QTL mapping results. The bin selected by SparseNet as a QTL is colored opaquely, and the nearby bins correlated to it at or greater than 0.9 are taken as an approximate QTL interval and colored translucently.

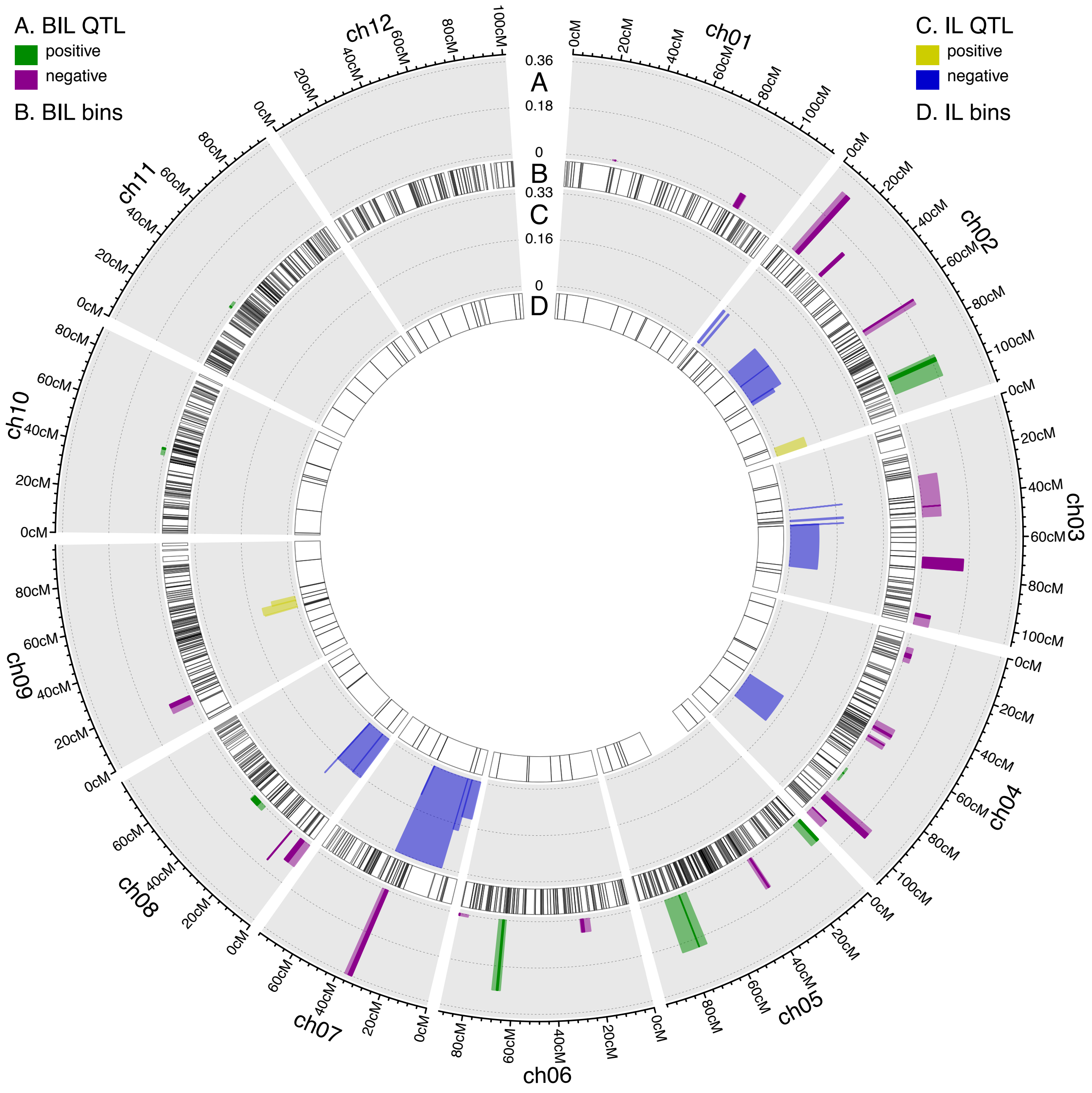

Figure S67. Fine-mapping and confirmation of leaf primary complexity QTL. The BIL bin selected by SparseNet as a QTL is colored opaquely, and the nearby bins correlated to it at or greater than 0.9 are taken as an approximate QTL interval and colored translucently.

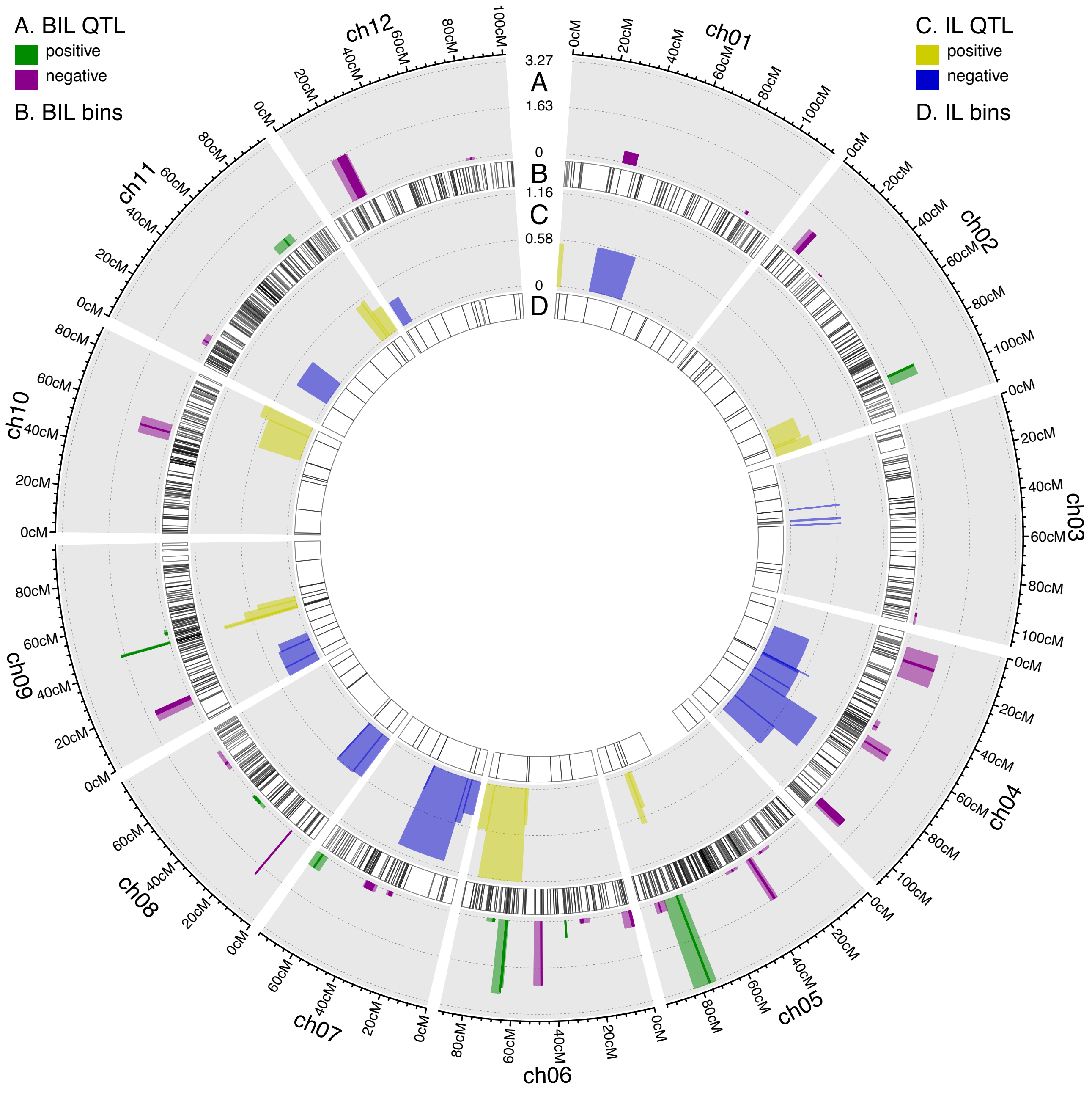

Figure S68. Fine-mapping and confirmation of leaf intercalary complexity QTL. The BIL bin selected by SparseNet as a QTL is colored opaquely, and the nearby bins correlated to it at or greater than 0.9 are taken as an approximate QTL interval and colored translucently.

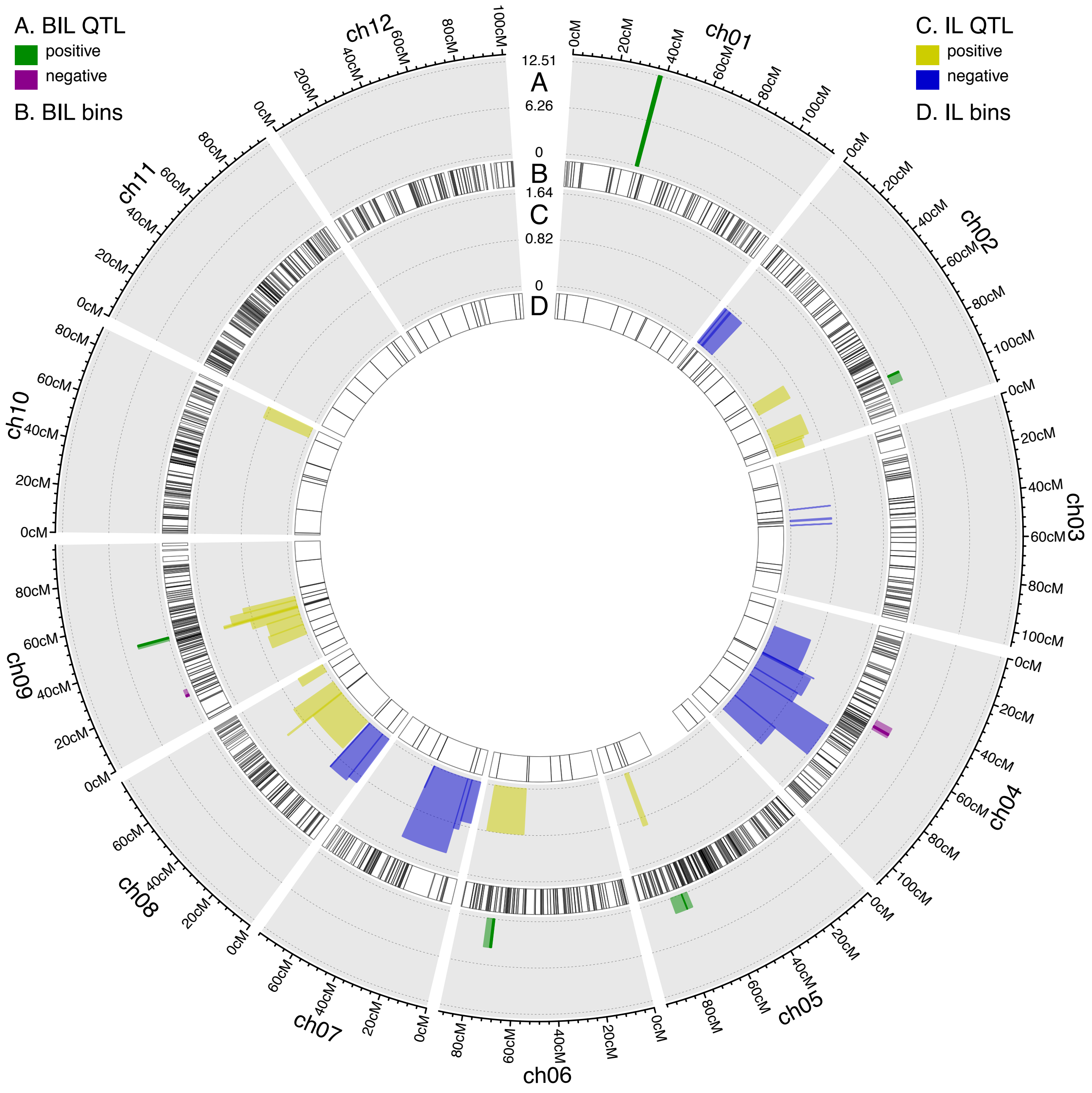

Figure S69. Fine-mapping and confirmation of leaf secondary complexity QTL. The BIL bin selected by SparseNet as a QTL is colored opaquely, and the nearby bins correlated to it at or greater than 0.9 are taken as an approximate QTL interval and colored translucently.

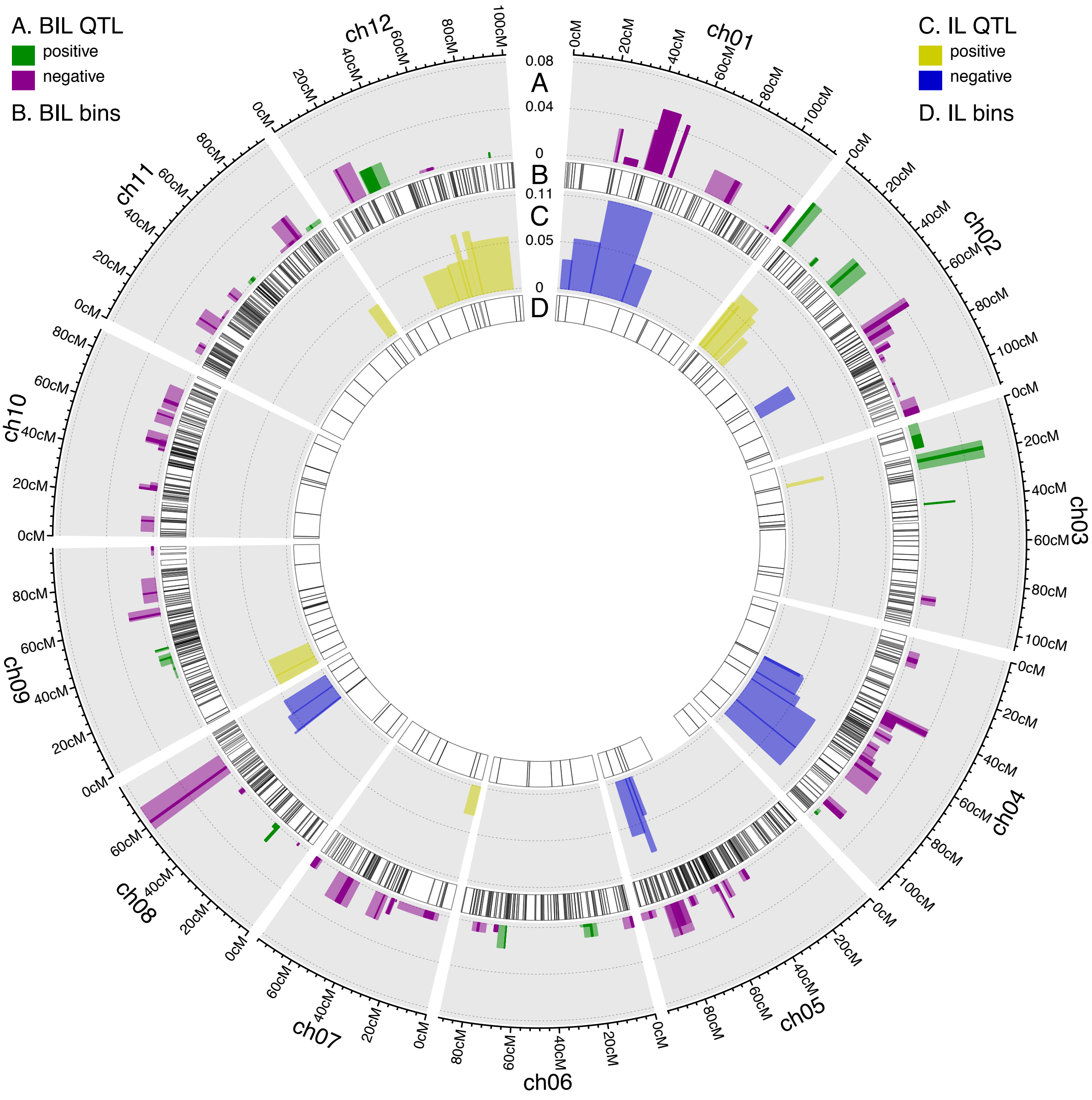

Figure S70. Fine-mapping and confirmation of leaflet aspect ratio QTL. The BIL bin selected by SparseNet as a QTL is colored opaquely, and the nearby bins correlated to it at or greater than 0.9 are taken as an approximate QTL interval and colored translucently.

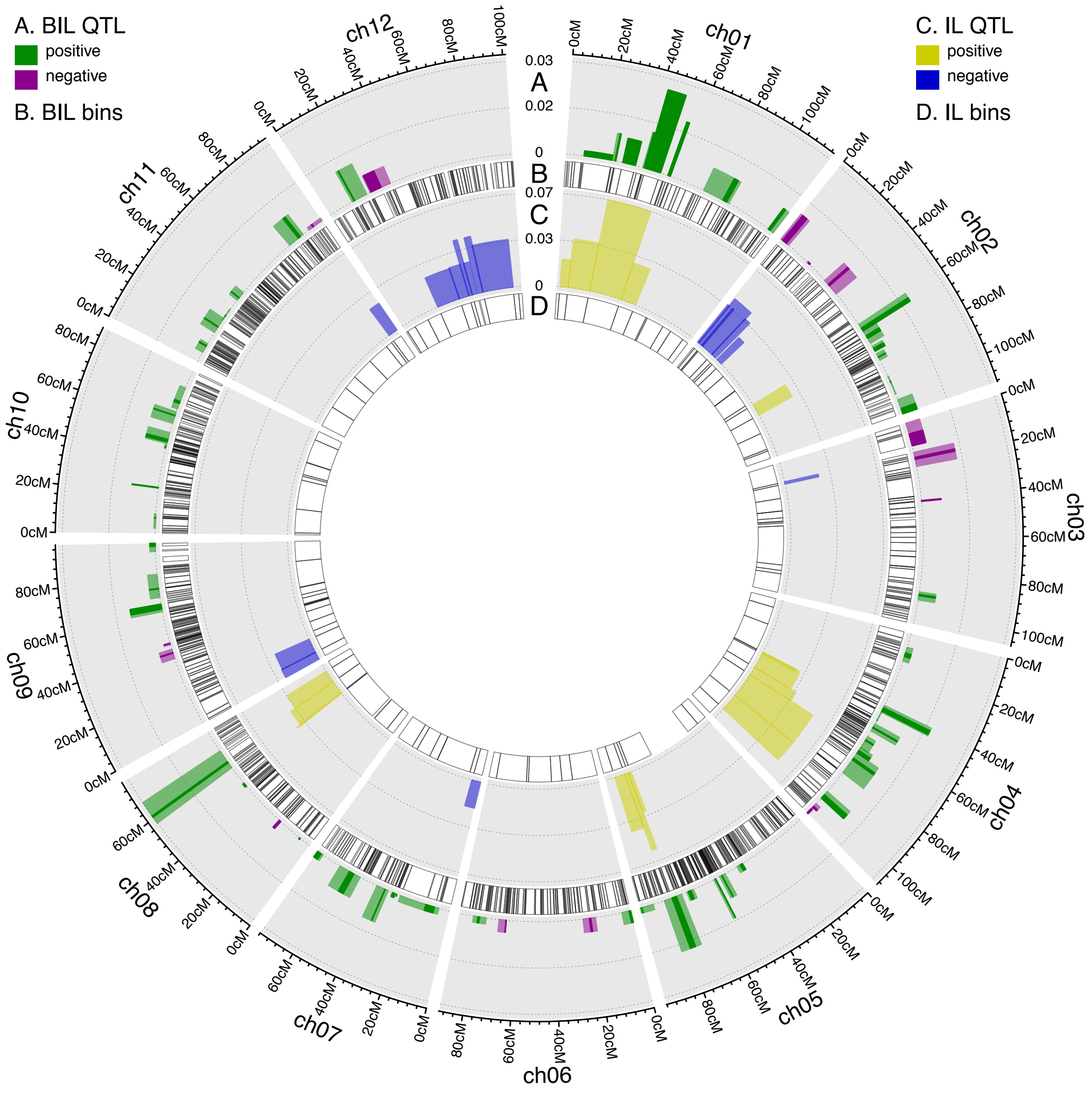

Figure S71. Fine-mapping and confirmation of leaflet roundness QTL. The BIL bin selected by SparseNet as a QTL is colored opaquely, and the nearby bins correlated to it at or greater than 0.9 are taken as an approximate QTL interval and colored translucently.

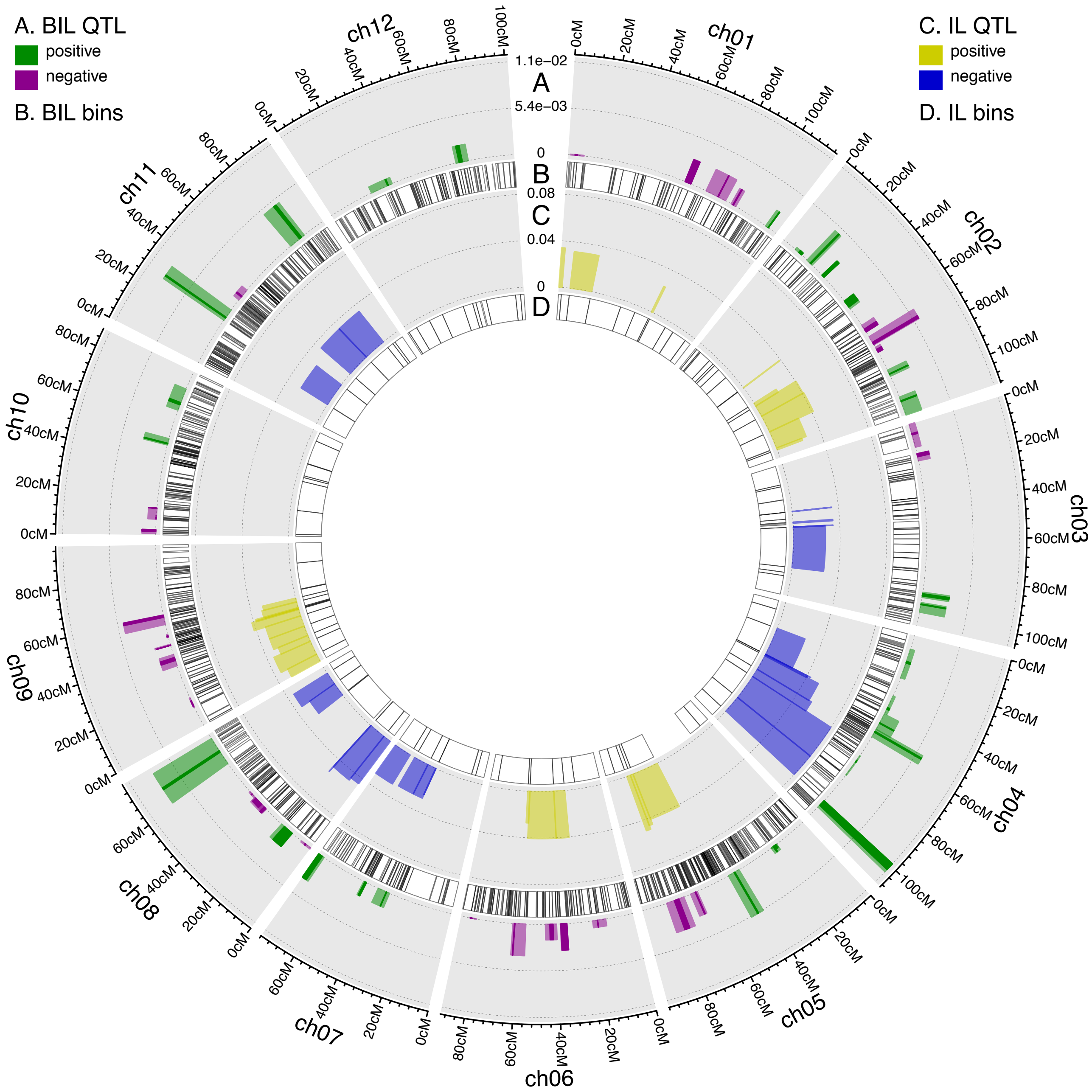

Figure S72. Fine-mapping and confirmation of leaflet solidity QTL. The BIL bin selected by SparseNet as a QTL is colored opaquely, and the nearby bins correlated to it at or greater than 0.9 are taken as an approximate QTL interval and colored translucently.

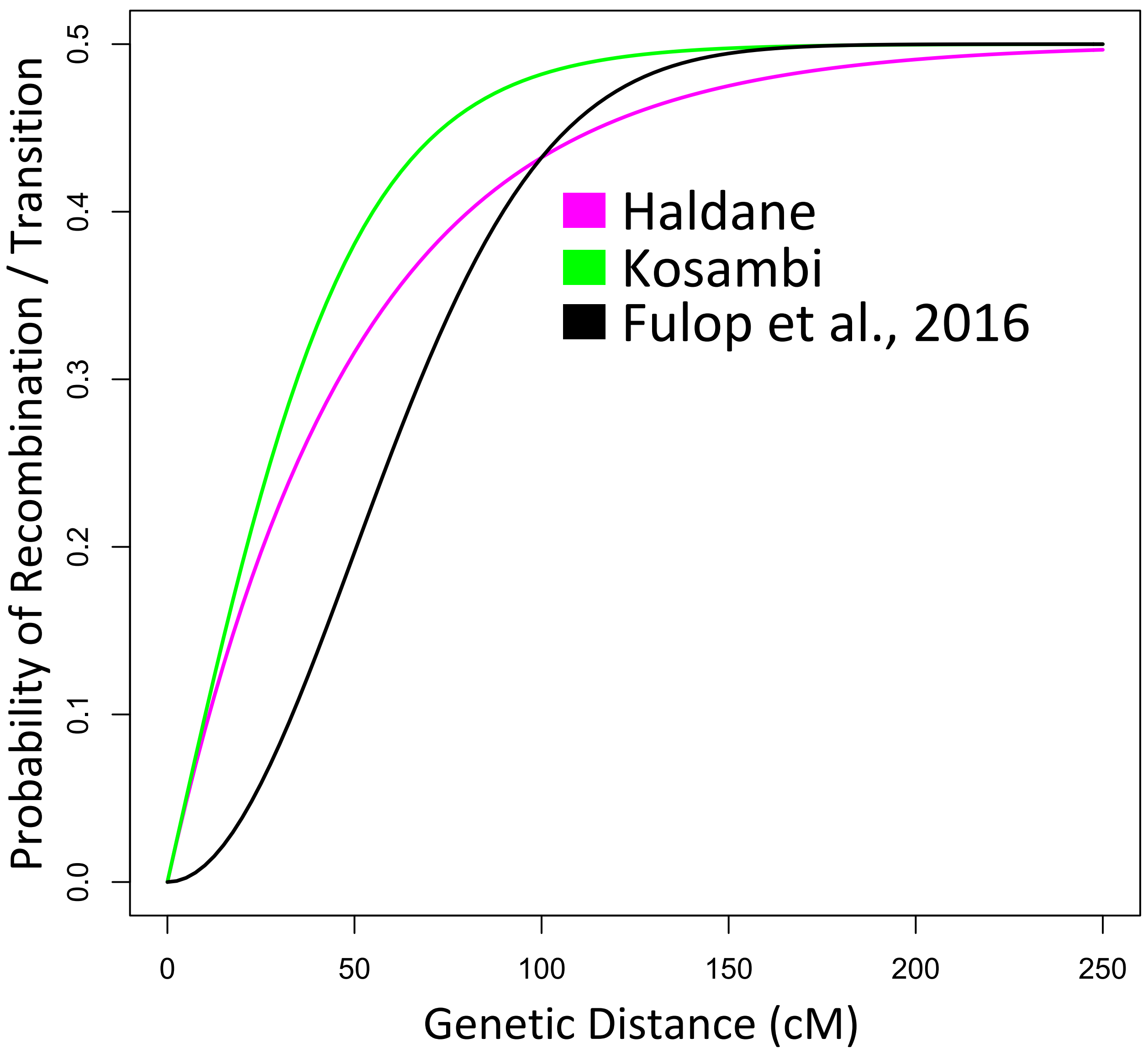

Figure S73. Heterogeneous Hidden Markov Model (HMM) hidden state transition model comparison.
