# Supplementary material for: A New Advanced Backcross Tomato Population Enables High Resolution Leaf QTL Mapping and Gene Identification
Source: G3 (Bethesda). 2016 Aug 10;6(10):3169–84. doi: 10.1534/g3.116.030536 (PMC5068939; doi:10.1534/g3.116.030536)
Supplement: Supplemental Material [file supp_g3.116.030536_TableS1.pdf]

Trait	H^2 MLE	H^2 MCMC mean	H^2 MCMC median	H^2 MCMC mode	H^2 MCMC lower CI	H^2 MCMC upper CI	R^2 MLE	R^2 MCMC mean	R^2 MCMC median	R^2 MCMC mode	R^2 MCMC lower CI	R^2 MCMC upper CI
**Primary Complexity**	22.77%	21.56%	22.11%	22.64%	14.60%	27.63%	47.05%	36.79%	37.89%	38.43%	28.73%	42.09%
**Intercalary Complexity**	41.42%	37.99%	39.77%	40.02%	22.20%	46.52%	67.98%	45.60%	47.50%	47.37%	34.07%	51.15%
**Secondary Complexity**	32.04%	27.79%	29.80%	30.07%	9.86%	37.58%	67.04%	45.89%	49.57%	50.59%	21.33%	54.20%
**Total Complexity**	39.54%	34.70%	37.22%	38.07%	13.19%	45.76%	71.83%	47.25%	50.59%	51.20%	25.16%	55.24%
**Leaflet Area**	6.02%	5.47%	5.61%	6.02%	1.97%	9.12%	65.25%	57.18%	60.89%	61.42%	29.13%	65.01%
**Leaflet Circularity**	26.98%	26.47%	26.74%	26.57%	22.45%	30.67%	44.67%	34.63%	35.08%	35.54%	32.79%	37.56%
**Leaflet Aspect Ratio**	27.07%	26.19%	26.74%	27.01%	21.42%	31.67%	41.69%	32.01%	32.64%	32.94%	28.60%	35.36%
**Leaflet Roundness**	27.96%	27.08%	27.60%	27.83%	21.93%	33.17%	42.97%	32.78%	33.41%	33.32%	29.31%	36.63%
**Leaflet Solidity**	25.57%	25.22%	25.44%	25.25%	21.25%	29.39%	39.46%	31.16%	31.45%	31.69%	29.27%	33.60%
**Symmetric EFD PC1**	5.94%	5.67%	5.76%	5.75%	3.82%	7.40%	20.66%	18.81%	19.31%	19.24%	15.09%	21.65%
**Symmetric EFD PC2**	13.66%	13.25%	13.55%	13.76%	11.00%	16.64%	25.81%	22.11%	22.67%	22.68%	20.01%	24.85%
**Symmetric EFD PC3**	15.28%	15.01%	15.17%	15.27%	12.41%	17.88%	29.37%	25.04%	25.41%	25.54%	23.42%	27.57%
**Symmetric EFD PC4**	3.44%	3.25%	3.28%	3.19%	2.23%	4.66%	15.71%	14.60%	15.05%	15.19%	11.39%	17.21%
**Symmetric EFD PC5**	4.99%	4.79%	4.88%	4.71%	3.37%	6.28%	12.07%	11.03%	11.29%	11.06%	8.21%	13.16%
**Symmetric EFD PC6**	3.32%	3.21%	3.24%	3.13%	2.30%	4.14%	5.91%	5.54%	5.61%	5.87%	4.18%	7.02%
**Symmetric EFD PC7**	3.10%	3.04%	3.07%	3.04%	2.08%	4.06%	6.61%	6.20%	6.30%	6.38%	4.74%	7.75%
**Symmetric EFD PC8**	3.65%	3.63%	3.65%	3.56%	2.66%	4.59%	8.64%	8.18%	8.25%	8.32%	6.82%	9.58%
**Symmetric EFD PC9**	2.54%	2.49%	2.51%	2.51%	1.63%	3.45%	7.75%	7.32%	7.45%	7.59%	5.69%	9.05%
**Asymmetric EFD PC1**	1.97%	1.89%	1.93%	1.92%	0.88%	2.86%	2.74%	2.53%	2.56%	2.78%	1.44%	3.87%
**Asymmetric EFD PC2**	3.48%	3.39%	3.42%	3.51%	2.14%	4.76%	6.94%	6.56%	6.65%	6.56%	5.15%	8.23%
**Asymmetric EFD PC3**	2.45%	2.38%	2.38%	2.28%	1.32%	3.46%	5.42%	5.17%	5.21%	5.27%	3.84%	6.51%
**Asymmetric EFD PC4**	3.11%	3.05%	3.05%	3.11%	2.03%	4.25%	5.50%	5.22%	5.24%	5.47%	4.09%	6.73%
**Asymmetric EFD PC5**	4.62%	4.52%	4.57%	4.56%	3.13%	5.97%	6.04%	5.57%	5.66%	5.56%	4.25%	7.19%
**Asymmetric EFD PC6**	3.16%	3.13%	3.14%	3.20%	2.05%	4.30%	5.38%	5.20%	5.21%	5.20%	4.00%	6.42%
**Asymmetric EFD PC7**	2.14%	2.04%	2.08%	2.09%	1.00%	3.16%	2.70%	2.50%	2.54%	2.40%	1.31%	3.81%

Table S1. Broad-sense heritability (H^2) and repeatability (R^2) estimates. MLE = Maximum Likelihood estimate. CI = Bayesian 95% credibility interval.
