# Supplementary material for: A New Advanced Backcross Tomato Population Enables High Resolution Leaf QTL Mapping and Gene Identification
Source: G3 (Bethesda). 2016 Aug 10;6(10):3169–84. doi: 10.1534/g3.116.030536 (PMC5068939; doi:10.1534/g3.116.030536)
Supplement: Supplemental Material [file supp_g3.116.030536_TableS2.pdf]

Traits	eQTL candidates		eQTL + PROVEAN candidates	
	No. candidate genes	p-value	No. candidate genes	p-value
**Primary comp.**	9	0.12	19	0.0776
**Secondary comp.**	0	1	4	0.419
**Intercalary comp.**	8	0.2212	18	0.1261
**Total comp.**	5	0.3297	12	0.1521
**Area**	NA	NA	NA	NA
**Aspect Ratio**	20	0.0139	29	0.6816
**Circularity**	11	0.0499	15	0.5633
**Roundness**	19	0.0099	28	0.5178
**Solidity**	16	0.0117	21	0.6244
**Symmmetric PC1**	2	0.7214	4	0.8246
**Symmmetric PC2**	9	0.1796	19	0.1417
**Symmmetric PC3**	16	0.0129	20	0.6918
**Symmmetric PC4**	4	0.2689	7	0.4184
**Asymmmetric PC1**	NA	NA	NA	NA
**Asymmmetric PC2**	1	0.6806	1	0.9277
**Asymmmetric PC3**	3	0.1236	4	0.3122
**Asymmmetric PC4**	5	1.00E-04	2	0.2244

Table S2. Permutation tests for candidate gene enrichment of BIL single locus QTL using a literature-curated leaf developmental gene list together with *cis*-eQTL and radical amino acid change (PROVEAN) data.
