# Supplementary material for: A New Advanced Backcross Tomato Population Enables High Resolution Leaf QTL Mapping and Gene Identification
Source: G3 (Bethesda). 2016 Aug 10;6(10):3169–84. doi: 10.1534/g3.116.030536 (PMC5068939; doi:10.1534/g3.116.030536)
Supplement: Supplemental Material [file supp_g3.116.030536_TableS3.pdf]

Epistatic QTL	Trait	IL gene chromosome	ITAG gene number	BIL chrom. 1	BIL interval 1 start	BIL interval 1 end	IL correlated bin's chromosome	IL correlated bin start	IL correlated bin end	IL bin	IL sub-bin	BIL chrom. 2	BIL interval 2 start	BIL interval 2 end
Leaf development gene cluster														
BIN_447_x_BIN_465	AR	ch06	Solyc06g007350	ch06	1	2490644	ch06	32169431	33752711	d.6B	d.6B	ch06	32261080	33251856
BIN_447_x_BIN_465	Round	ch06	Solyc06g007350	ch06	1	2490644	ch06	32169431	33752711	d.6B	d.6B	ch06	32261080	33251856
BIN_447_x_BIN_878	all	ch06	Solyc06g007350	ch06	1	2490644	ch11	2475481	4841505	d.11B	d.11B	ch11	2502553	3508263
BIN_447_x_BIN_878	int	ch06	Solyc06g007350	ch06	1	2490644	ch11	2475481	4841505	d.11B	d.11B	ch11	2502553	3508263
Leaf development + photosynthesis gene clusters														
BIN_69_x_BIN_74	AR	ch02	Solyc02g062610	ch02	21807683	29359068	ch02	29893130	30876102	d.2C	d.2C	ch02	30663959	31191063
BIN_447_x_BIN_465	AR	ch06	Solyc06g007350	ch06	1	2490644	ch06	32169431	33752711	d.6B	d.6B	ch06	32261080	33251856
BIN_447_x_BIN_878	int	ch06	Solyc06g007350	ch06	1	2490644	ch11	2475481	4841505	d.11B	d.11B	ch11	2502553	3508263
BIN_447_x_BIN_465	Round	ch06	Solyc06g007350	ch06	1	2490644	ch06	32169431	33752711	d.6B	d.6B	ch06	32261080	33251856
BIN_447_x_BIN_878	all	ch06	Solyc06g007350	ch06	1	2490644	ch11	2475481	4841505	d.11B	d.11B	ch11	2502553	3508263
BIN_447_x_BIN_465	AR	ch06	Solyc06g007590	ch06	1	2490644	ch06	32169431	33752711	d.6B	d.6B	ch06	32261080	33251856
BIN_447_x_BIN_465	Round	ch06	Solyc06g007590	ch06	1	2490644	ch06	32169431	33752711	d.6B	d.6B	ch06	32261080	33251856
BIN_447_x_BIN_465	Round	ch06	Solyc06g008160	ch06	1	2490644	ch06	32169431	33752711	d.6B	d.6B	ch06	32261080	33251856
BIN_447_x_BIN_465	AR	ch06	Solyc06g008160	ch06	1	2490644	ch06	32169431	33752711	d.6B	d.6B	ch06	32261080	33251856
BIN_447_x_BIN_465	Round	ch06	Solyc06g008500	ch06	1	2490644	ch06	32169431	33752711	d.6B	d.6B	ch06	32261080	33251856
BIN_447_x_BIN_465	AR	ch06	Solyc06g008500	ch06	1	2490644	ch06	32169431	33752711	d.6B	d.6B	ch06	32261080	33251856
BIN_581_x_BIN_825	AR	ch07	Solyc07g065860	ch07	64414586	65268621	ch10	62171739	64083409	d.10F	d.10F	ch10	61898239	62526575
BIN_581_x_BIN_825	Round	ch07	Solyc07g065860	ch07	64414586	65268621	ch10	62171739	64083409	d.10F	d.10F	ch10	61898239	62526575
BIN_594_x_BIN_689	sec	ch09	Solyc09g059240	ch08	48414668	48539772	ch08	3034542	50543946	d.8B	d.8B	ch09	19245879	60792747
BIN_594_x_BIN_694	int	ch09	Solyc09g059240	ch08	48414668	48539772	ch08	3034542	50543946	d.8B	d.8B	ch09	44443481	57760631

Table S3. IL *trans*-eQTL enrichment among BIL epistatic QTL.
