# Supplementary material for: A New Advanced Backcross Tomato Population Enables High Resolution Leaf QTL Mapping and Gene Identification
Source: G3 (Bethesda). 2016 Aug 10;6(10):3169–84. doi: 10.1534/g3.116.030536 (PMC5068939; doi:10.1534/g3.116.030536)
Supplement: Supplemental Material [file supp_g3.116.030536_TableS4.pdf]

Table S4. Genes in the overlapping interval of the chromosome 5 flowering QTL in ILs and BILs.

ITAG gene number	Human-readable description
**Solyc05g053680.1.1**	Pectinesterase (AHRD V1 **** B9RWI7_RICCO); contains Interpro domain(s) IPR000070 Pectinesterase, catalytic
**Solyc05g053690.1.1**	Ubiquitin carboxyl-terminal hydrolase (AHRD V1 **** B9GFW8_POPTR); contains Interpro domain(s) IPR001394 Peptidase C19, ubiquitin carboxyl-terminal hydrolase 2
**Solyc05g053700.1.1**	Xyloglucan endotransglucosylase/hydrolase 1 (AHRD V1 **** D5HRN0_FRAAN); contains Interpro domain(s) IPR016455 Xyloglucan endotransglucosylase/hydrolase
**Solyc05g053710.2.1**	TBC1 domain family member 13 (AHRD V1 ***- D3BTH5_POLPA); contains Interpro domain(s) IPR000195 RabGAP/TBC
**Solyc05g053720.2.1**	Os12g0425600 protein (Fragment) (AHRD V1 **-- Q0INN2_ORYSJ); contains Interpro domain(s) IPR004348 Protein of unknown function DUF246, plant
**Solyc05g053730.2.1**	Genomic DNA chromosome 5 TAC clone K21P3 (AHRD V1 *-*- Q9FJ19_ARATH)
**Solyc05g053740.2.1**	Alkaline ceramidase (AHRD V1 ***- Q17KM2_AEDAE); contains Interpro domain(s) IPR008901 Alkaline phytoceramidase
**Solyc05g053750.1.1**	Lipase (AHRD V1 **** Q1XBG1_RICCO); contains Interpro domain(s) IPR006693 AB-hydrolase associated lipase region
**Solyc05g053760.2.1**	Chaperone protein dnaJ 20 (AHRD V1 ***- B6U349_MAIZE); contains Interpro domain(s) IPR001623 Heat shock protein DnaJ, N-terminal
**Solyc05g053770.2.1**	At5g48610-like protein (Fragment) (AHRD V1 *-*- Q6XWA2_ARATH)
**Solyc05g053780.2.1**	RNA binding protein-like protein (AHRD V1 ***- Q3HVL3_SOLTU); contains Interpro domain(s) IPR015465 RNA recognition motif, glycine rich protein
**Solyc05g053790.1.1**	UDP-D-glucuronate 4-epimerase 4 (AHRD V1 **** D7LCH5_ARALY); contains Interpro domain(s) IPR016040 NAD(P)-binding domain
**Solyc05g053800.2.1**	DNA repair protein Rad50 (AHRD V1 ***- A8IE27_TRITU); contains Interpro domain(s) IPR004584 Recombination/repair protein Rad50
**Solyc05g053810.2.1**	Serine hydroxymethyltransferase (AHRD V1 **** D2D306_GOSHI); contains Interpro domain(s) IPR001085 Serine hydroxymethyltransferase
**Solyc05g053820.2.1**	UDP-glucosyltransferase family 1 protein (AHRD V1 **** C6KI44_CITSI); contains Interpro domain(s) IPR002213 UDP-glucuronosyl/UDP-glucosyltransferase
**Solyc05g053830.2.1**	Protein transport protein Sec23 (AHRD V1 ***- Q9LUG1_ARATH); contains Interpro domain(s) IPR006896 Sec23/Sec24 trunk region
**Solyc05g053850.2.1**	SELF PRUNING 5G
**Solyc05g053860.2.1**	Solute carrier family 22 member 5 (Predicted) (AHRD V1 ***- B3RF59_SORAR); contains Interpro domain(s) IPR016196 Major facilitator superfamily, general substrate transporter
**Solyc05g053870.2.1**	Copine I-like protein (AHRD V1 ***- Q6YYC5_ORYSJ); contains Interpro domain(s) IPR010734 Copine
**Solyc05g053880.2.1**	cDNA FLJ42396 fis clone ASTRO2001107 (AHRD V1 *-*- B3KW72_HUMAN)
**Solyc05g053890.1.1**	Glucosyltransferase-like protein (AHRD V1 *-** Q9LXV0_ARATH); contains Interpro domain(s) IPR002213 UDP-glucuronosyl/UDP-glucosyltransferase
**Solyc05g053900.2.1**	Aspartic proteinase nepenthesin-2 (AHRD V1 **-- B6TNP6_MAIZE); contains Interpro domain(s) IPR001461 Peptidase A1
**Solyc05g053910.1.1**	Lipase family protein (AHRD V1 ***- Q2RAM0_ORYSJ); contains Interpro domain(s) IPR002921 Lipase, class 3
**Solyc05g053920.1.1**	Lipase (Fragment) (AHRD V1 ***- Q9ZTW1_DIACA); contains Interpro domain(s) IPR002921 Lipase, class 3
**Solyc05g053930.2.1**	ATP binding / serine-threonine kinase (AHRD V1 **** C5DB71_VITVI); contains Interpro domain(s) IPR002290 Serine/threonine protein kinase
**Solyc05g053940.2.1**	Ras-related protein Rab-1A (AHRD V1 ***- B6RB23_HALDI); contains Interpro domain(s) IPR003579 Ras small GTPase, Rab type
**Solyc05g053950.2.1**	Plasmid partition ParA protein (AHRD V1 ***- B8KJV3_9GAMM); contains Interpro domain(s) IPR010775 Protein of unknown function DUF1365
**Solyc05g053960.2.1**	Cysteine-rich extensin-like protein-2 (AHRD V1 *-*- Q08195_TOBAC)
**Solyc05g053970.2.1**	Amino acid transporter (AHRD V1 **** B9HY05_POPTR); contains Interpro domain(s) IPR013057 Amino acid transporter, transmembrane
**Solyc05g053980.1.1**	NBS-LRR resistance protein-like protein (AHRD V1 ***- A1Y9Q9_SOLLC)
**Solyc05g053990.2.1**	Cc-nbs-lrr, resistance protein
**Solyc05g054000.2.1**	Late blight resistance protein Rpi-blb2 (AHRD V1 ***- Q38QB6_SOLBU)
**Solyc05g054010.2.1**	Cc-nbs-lrr, resistance protein
**Solyc05g054020.1.1**	Cc-nbs-lrr, resistance protein
**Solyc05g054030.2.1**	C2H2L domain class transcription factor (AHRD V1 **-* D9ZIU3_MALDO); contains Interpro domain(s) IPR007087 Zinc finger, C2H2-type
**Solyc05g054040.2.1**	Transmembrane 9 superfamily protein member 1 (AHRD V1 **** B6TZ24_MAIZE); contains Interpro domain(s) IPR004240 Nonaspanin (TM9SF)
**Solyc05g054050.2.1**	Glutamate decarboxylase (AHRD V1 **** Q8LKR4_TOBAC); contains Interpro domain(s) IPR010107 Glutamate decarboxylase
